# Comparative genomics of the wheat fungal pathogen *Pyrenophora tritici-repentis* reveals chromosomal variations and genome plasticity

**DOI:** 10.1186/s12864-018-4680-3

**Published:** 2018-04-23

**Authors:** Paula Moolhuijzen, Pao Theen See, James K. Hane, Gongjun Shi, Zhaohui Liu, Richard P. Oliver, Caroline S. Moffat

**Affiliations:** 1 Centre for Crop Disease and Management, Department of Environment and Agriculture, Curtin University, Bentley, Western Australia Australia; 2 Department of Plant Pathology, North Dakota State University, Fargo, ND USA

**Keywords:** Wheat, Fungal pathogen, Comparative genomics, ToxA, Race

## Abstract

**Background:**

*Pyrenophora tritici-repentis* (Ptr) is a necrotrophic fungal pathogen that causes the major wheat disease, tan spot. We set out to provide essential genomics-based resources in order to better understand the pathogenicity mechanisms of this important pathogen.

**Results:**

Here, we present eight new Ptr isolate genomes, assembled and annotated; representing races 1, 2 and 5, and a new race. We report a high quality Ptr reference genome, sequenced by PacBio technology with Illumina paired-end data support and optical mapping. An estimated 98% of the genome coverage was mapped to 10 chromosomal groups, using a two-enzyme hybrid approach. The final reference genome was 40.9 Mb and contained a total of 13,797 annotated genes, supported by transcriptomic and proteogenomics data sets.

**Conclusions:**

Whole genome comparative analysis revealed major chromosomal segmental rearrangements and fusions, highlighting intraspecific genome plasticity in this species. Furthermore, the Ptr race classification was not supported at the whole genome level, as phylogenetic analysis did not cluster the ToxA producing isolates. This expansion of available Ptr genomics resources will directly facilitate research aimed at controlling tan spot disease.

**Electronic supplementary material:**

The online version of this article (10.1186/s12864-018-4680-3) contains supplementary material, which is available to authorized users.

## Background

The necrotrophic fungal pathogen *Pyrenophora tritici-repentis* [(Died.) Drechs.] [anamorph: *Drechslera tritici-repentis* (Died.) Shoem.] (abbreviated to Ptr) is the causal agent of tan spot, a major disease of wheat globally. Ptr is an ascomycete fungus within the class Dothideomycetes and order Pleosporales, which also contains other important crop pathogens [1]. Disease symptoms are typically necrotic leaf lesions surrounded by a chlorotic halo, which coalesce and reduce the leaf area available for photosynthesis as the infection progresses, impacting grain fill and quality. Epidemics have increased in frequency and severity since the 1970s, and yield losses have been reported of up to 50% on susceptible varieties [2, 3]. In Australia, the value of tan spot disease control is estimated at $463 million annually, in addition to a direct yield loss of $212 million, making it the country’s most significant wheat disease [4]. It is widely distributed in North and South America, North Africa and Europe [5] and total world losses are estimated at 15 million metric tons, second only to Septoria tritici blotch [6].

Ptr utilises a number of approaches to penetrate wheat host cells, which include the secretion of plant cell wall degrading enzymes, reactive oxygen species, secondary metabolites, necrotrophic effectors and other phytotoxins [7, 8]. The two major Ptr proteinaceous effectors are Ptr ToxA and Ptr ToxB that produce necrotic and chlorotic symptoms respectively on wheat genotypes possessing the corresponding host sensitivity genes [9–13]. A third but elusive effector, Ptr ToxC, is reported to be a polar, non-ionic, low-molecular-weight molecule [14] that also causes leaf chlorosis. The combination of these three effectors have come to define the current eight race categories for Ptr [15]. Pathogenic race classifications are, race 1 (ToxA and ToxC), race 2 (ToxA), race 3 (ToxC), race 5 (ToxB), race 6 (ToxB and ToxC), race 7 (ToxA and ToxB) and race 8 (ToxA, ToxB and ToxC). Isolates without ToxA, ToxB or ToxC are placed in race 4. Race 4 isolates also have an inactive *toxb* ortholog which shares 81% amino acid identity to the active form of *ToxB* [16] and are non-pathogenic. However, the current race structure requires revision as the existence of new races and novel unidentified effectors beyond ToxA, ToxB and ToxC has been reported [17–22]. For example, a North American isolate (AR CrossB10) has ToxC, and lacks ToxA and ToxB, but is still able to cause necrosis on the Ptr ToxA differential wheat cultivar Glenlea [20–22]. This implies that AR CrossB10 is a new race that produces an unidentified effector capable of causing necrosis.

Previous work has examined the genetic diversity of isolates of different races. Based on pulse field gel electrophoresis, the haploid number of chromosomes and sizes for Ptr were found to be as variable within races as between and 47 Ptr isolates were grouped into 29 different karyotypes [23]. Earlier studies used molecular markers to evaluate Ptr isolates from diverse geographic locations, including random amplified polymorphic DNA (RAPD) [24–27], amplified length fragment polymorphism (AFLP) [28, 29] and inter-simple sequence repeats (ISSR) [30]. Most analyses found no significant relationship between DNA polymorphisms and geographic origin or race classification [23–26, 28, 30]. However, some studies did identify a correlation. For example, an AFLP study found genetic similarity was independent of race classification, with the exception of race 4 [29]. In another report, it was possible to group isolates by their geographic origin based on SSRs across eight races [31]. Clear genetic differentiation was also observed between Ptr ToxA-nonproducing (races 3, 4, 5 and 6) and Ptr ToxA-producing isolates (races 1, 2, 7 and 8), and it was concluded that races 1 and 2 were more closely related to races 7 and 8, but more distant to races 3 and 5 [31].

These early studies focused on a limited selection of molecular markers to assess genetic diversity across races, which were not representative of the entire genome. However the release of the genome sequences of three North American isolates based on Sanger and Illumina sequencing, permitted preliminary comparative genomic analysis [32]. The race 1 isolate, Pt-1C-BFP (BFP) was assembled with a total length of 38 Mb and 90% of the DNA scaffolds were mapped to 11 optical linkage groups. The other two genomes were of a race 5 isolate (DW7) and a race 4 isolate (SD20), and were released as unassembled short paired-end (PE) reads (75 bp) [32]. Genome analysis of the three races revealed a distinct *ToxA* containing region of 145 kb, which was only found in the race 1 isolate and not in the race 4 or 5 isolates. In addition, a greater genomic divergence was found for the pathogenic isolates compared to the race 4 isolate. The optically mapped BFP genome was highly scaffolded and contained approximately 700 sequence gaps, totalling over 2 Mb in length. The number and length of sequence gaps is due to the limitations of the sequencing technology available at the time. Gapped assemblies are a common limitation of shorter read technologies, however, extraordinary progress has been made in genome sequencing technologies since the release of BFP. Long read single-molecule real-time (SMRT) sequencing technologies, such as PacBio [33] have overcome the short read length limitation by producing reads with an average length of approximately 14 kb, albeit with a higher base error rate (~ 15%) [34]. The PacBio RSII read quality was improved using cyclic bell sequencing to produce a sufficiently high read coverage (>50×), such that the longest subset of reads with a relatively high rate of random errors are effectively corrected by overlapping the shorter reads [34]. These corrected long reads can then be used for contig assembly, and the assembly quality can be further improved by performing ‘genome polishing’ utilizing high quality Illumina short reads [35]. The high quality contiguous sequences produced help to resolve long genomic repetitive content, and can almost represent whole fungal chromosomes, telomere to telomere [36].

The combinations of long read and optical mapping technologies are powerful tools to understanding genome structure. Optical Mapping provided high-resolution sequence motif physical maps using restriction-nicking enzymes rather than direct sequence information. Technologies such as the NanoChannel Array (Irys System) from BioNano Genomics (BioNano) can hold linear DNA molecules up to megabases in length, to produce motif maps that can be de novo assembled into chromosomal maps to scaffold genome sequence assemblies [37].

Here, we report a high quality reference genome of an Australian race 1 isolate (M4). M4 was constructed based on PacBio long read sequence, Illumina data and BioNano two-enzyme optical mapping technologies. In addition, we present whole genome comparative analysis of 11 Ptr isolates from five different races. This comprises 8 new Ptr genomes, assembled and annotated, representing races 1, 2, 5 and a new race, as well as the assembly and annotation of raw read data from SD20 and DW7 (races 4 and 5) [32]. To identify the Ptr core and accessory gene components across diverse isolates from globally distant locations, we also re-annotated the BFP genome using consistent bioinformatics workflows to conduct comparative analyses.

The aims of this study were 1) To generate a high quality reference genome assembly of an Australian race 1 isolate (M4) in order to improve the available genome resources for this globally significant wheat pathogen, 2) To expand available genomics-based resources by sequencing new Ptr races 3) To test by whole genome comparative analyses the current Ptr race classification, and 4) To identify Ptr core and accessory gene sets to identify novel effector candidates.

## Results

The genome of Ptr isolate M4 was sequenced using long read single molecule real time (SMRT) PacBio sequencing technology error corrected at 75X coverage, and assembled de novo into 50 contiguous (ungapped) sequences with a total length of 40.9 Mb. The assembled genome base calls were corrected (termed polishing) with M4 Illumina high-quality paired-end reads, correcting a total of 11,715 bases. The final genome had a contig L50 of 6, N50 length of 2.9 Mb and maximum contig length of 5.6 Mb (Table 1).

**Table 1:** Isolate source, race and genome de novo assembly statistics

	M4^a^	BFP^b^	134	239	5213	11137	86-124	AR CrossB10	DW5	DW7^c^	SD20^c^
Sequencing Platform	PacBio	Sanger, Illumina	Illumina	Illumina	Illumina	Illumina	Illumina	Illumina	Illumina	Illumina	Illumina
PE Read length (bp)	N/A	N/A	100	100	100	100	150	150	100	75	75
Genome accession^d^	NQIK00000000	N/A	MVBF00000000	MVBH00000000	MVBI00000000	MVBG00000000	NRDI00000000	NQWZ00000000	MUXC00000000	N/A	N/A
*Isolate information*											
Collection site	Western Australia	South Dakota, USA	Queensland, Australia	Victoria, Australia	Queensland, Australia	Western Australia	Canada	Arkansas, USA	North Dakota, USA	North Dakota, USA	South Dakota, USA
Collection year	2009	1994^e^	2001	2003	1987	2001	1989	2002	1998	1998	1998^f^
Race	1	1	1	1	1	1	2	New^k^	5	5	4
Effectors	AC	AC	AC	AC	AC	AC	A	C	B	B	b
*Optical map*											
% Mapped	98	90	N/A	N/A	N/A	N/A	N/A	N/A	N/A	N/A	N/A
*Contig assembly statistics*											
Total length (Mb)	40.9	38.0	34.0	34.4	34.1	33.9	34.4	33.8	33.4	32.9	34.1
Number	50	48	3579	3309	3318	2645	3223	2867	3964	3533	7322
N50 (Mb)	2.930	1.900	0.064	0.065	0.066	0.074	0.060	0.058	0.045	0.028	0.014
L50	6	6	150	148	150	130	168	171	208	348	661
Mean (Kb)	802	778	9.5	10.4	10.3	12.8	10.3	11.3	8.4	9.3	4.6
Max (Mb)	5.60	6.70	0.29	0.53	0.53	0.38	0.26	0.34	0.22	0.12	0.08
GC %	50.73	50.89	50.86	50.84	50.85	50.87	51.35	50.90	50.90	50.89	50.79
% Repeat^h^	7.96	5.87	2.64	2.62	2.58	2.33	2.65	2.71	2.36	1.88	1.97
RNA-Seq^i^	33.55	21.92^g^	22.34	21.00	23.00	21.22	19.87	19.92	24.44	20.63^g^	16.10^g^
% RNA-Seq^j^	85.1	79.4–81.4	81.1	81.2	82.2	80.9	76.0	76.2	81.9	74.2–78.5	58.2–60.0

^a^PacBio contiguous sequence, ^b^The BFP genome was downloaded from DDBJ/ENA/GenBank. Gene predictions were made on the scaffolds sourced from GenBank, ^c^Raw reads were downloaded from NCBI SRA and assembled for gene predictions. ^d^New Genbank WGS submissions (NA = Not Applicable), ^e^Isolate was subcultured from Pt-1C [12] ^f^SD20 collection [13]. ^g^Average read alignment across six isolates. ^h^Percentage of genome masked for RepBase (“Fungi”) repeats [38]. ^i^Number of concordant aligned RNA-Seq paired reads (millions). ^j^Percentage RNA-read concordant alignment, ^k^isolate does not meet race 1–8 classification

M4 was optically mapped with two restriction enzymes (BbvCl and BspQl), at 100× average depth of molecule coverage and assembled into 10 hybrid maps/chromosomes (Additional file 1). The M4 optical map then facilitated the anchoring/structuring of M4 contigs into 10 M4 chromosomes. In total, 39.9 Mb (98%) of the sequence assembly was chromosomally placed. Optical mapping supported the assembly of M4 contigs 2 to 16 and 18 to 20, but did not resolve the largest M4 contig 1 (7 Mb) at a highly complex and long repetitive region (Additional file 1). Contig 1 was therefore provisionally split at position 4,166,914 bp into contigs 1A and B. Contig 17 was M4 mitochondrial genome and not optically mapped.

The M4 reference genome assembly is 2.9 Mb larger than BFP (38 Mb) [32], (Table 1). The extra M4 sequence resolved distal telomeric and subtelomeric regions and nearly 700 gaps in BFP, the largest being a 700 kb gap in BFP chromosome 2 (positioned at 4.25–4.95 Mb). The annotated M4 whole genome sequence has been deposited at DDBJ/ENA/GenBank under the accession NQIK00000000. The version described in this paper is version NQIK01000000. The full M4 mitochondrial sequence was also assembled into a single sequence length of 183 kb, 26 kb larger than that of BFP.

### Genome wide comparative analysis of M4 and BFP

**Fig. 1:**
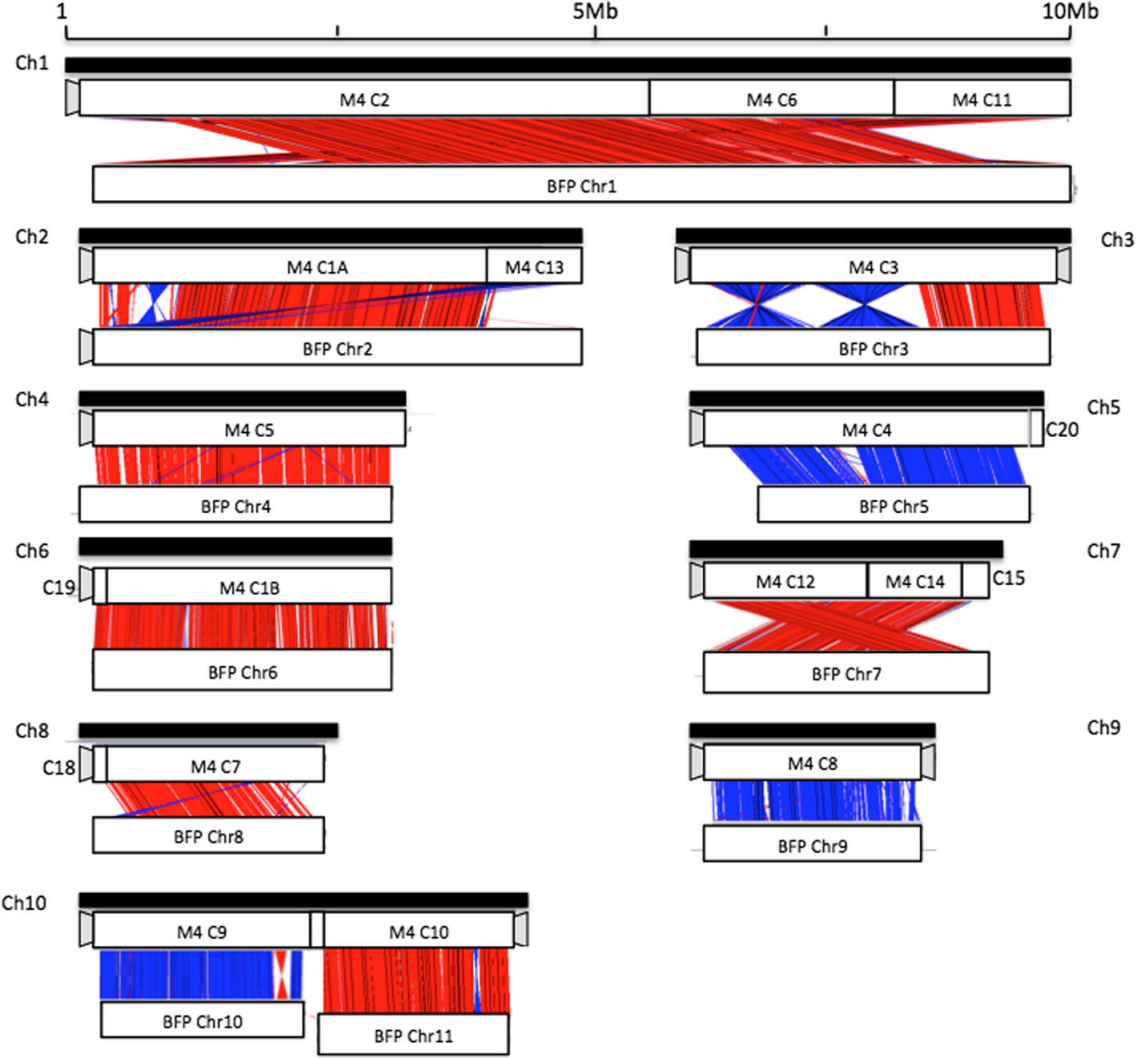
M4 Optical Map groups and genome alignment for M4 and BFP. Genome alignments of M4 contigs (M4 C) to BFP chromosomes (BFP Chr) are shown at ≥90% identity and ≥ 5 kb in length. M4 two-enzyme optical maps (black bars Ch1-10) are shown above, and M4 contig alignments to BFP chromosomes below. Red connecting lines represent sequence alignments in the same orientation between M4 and BFP, and reverse-complemented alignments are blue. Grey markers indicate distal ends of contigs with identifiable telomeric tandem repeat motifs

Whole genome comparative analysis was conducted between the M4 genome assembly and the published genome of BFP. BFP scaffolds were ordered and orientated into chromosomes using the published AGAP files available at the Broad Institute (ftp.broadinstitute.org/pub/annotation/fungi/pyrenophora/). Several types of sequence rearrangements were observed between the two assembled genomes, which included translocations, inversions and fusions (Fig. 1). The karyotypic context of these structural variations were informed by the identification of TTAGGG/CCCTAA telomeric tandem repeats at the ends of M4 contigs, which were absent from the BFP sequence. For simplicity, the M4 and BFP chromosomes are referred to as Ch and Chr respectively. The M4 two enzyme optical maps for Ch3, Ch6 and Ch10 had telomere motifs at both ends, whereas Ch1, Ch2, Ch4, Ch5, Ch7, Ch8, and Ch9 each had single telomere motifs. The optical map of M4 Ch10 appears to be the fused equivalent of BFP Chr 10 and 11. Large-scale rearrangement events between M4 and BFP also appeared in M4 Ch1, Ch7 and Ch2. There were also several instances of inverse segmental rearrangements in M4 Ch3 and Ch10, with two inversions on Ch3 comprising 60% of the chromosome length. Translocation of a distal chromosome region from one end of a chromosome to the other was also observed between M4 Ch2 (contig 13) and BFP Chr2.

To validate the M4 sequence involved in rearrangements, primers were designed to target the sequences. Primer sequences identified as unique in the M4 genome by an in silico search were tested and confirmed by PCR in M4 and DW5, and by in silico searches in BFP, as a DNA sample of BFP was unavailable. PCR confirmed M4 sequence that coincided with scaffold gaps in BFP (Additional file 2).

### Repetitive DNA in the M4 genome

To determine the level of repeat content in M4, repeats were identified against known transposable elements (TEs) deposited in RepBase (taxon “fungi”) [38], and from de novo predictions that also included large segmental duplications and long terminal repeats (LTRs) (Additional file 3). Fungal RepBase searches found M4 had 2.78 Mb (6.79%) of known interspersed repeats [38]. Retroelements comprised 74% of interspersed repeats, with the majority (46%) belonging to the LTR Copia element class, whereas DNA transposons comprised only 25% (Additional file 4).

As LTRs have been suggested to be associated with the expansion of gene families [19], a genome-wide search of M4 was conducted using LTR Harvest [39] and a total of 285 LTRs between 200 and 5000 bp lengths were counted. An LTR digest analysis showed that the majority of elements contained polypurine tracts between the LTRs, and were devoid of internal gag or pol genes.

A de novo repeat search on the RepBase masked genome identified 540 extra repeat families, covering an additional 2.8 Mb (7.04%) of repetitive DNA. The total M4 repeat element content (known and novel) was 15%. Only a fifth of de novo repeats families (155) contained a protein domain match. The majority of these were TE-related domains; a number of non-TE domains were identified with transporter and secondary metabolite related domains (Additional file 5). To estimate larger segmental duplication richness not identified as repeat families, M4 was searched for regions with greater than 90% sequence identity and greater than 5 kb in length. A total of 255 regions were identified, covering 1.9 Mb of the genome. Of these, 183 inter-segmental duplication events covered 536,793 bp (7.6%) of M4 Ch2 and Ch6. Regions of high duplication occurred mainly in telomeres and contig break points (Additional file 6).

One of the major drivers of fungal genome evolution is repeat-induced point mutation (RIP), a repeat-targeted mutagenesis mechanism that alters cytosine bases to thymine, with a bias for CpA dinucleotides [40, 41]. To identify if RIP was active in M4, de novo repeat families were searched for RIP signatures [42], and only 22 repeat families showed evidence of a CpA➔TpA bias. Only four repeat families had both a detectable RIP-like mutation bias and matched transposase-like domains. RIP indexing across the M4 genome confirmed a few identifiable genomic regions subject to RIP corresponding to repetitive regions (Additional file 7). Related to the RIP analyses, tests were performed to determine if M4 exhibited a “two speed genome” pattern of genome mutations as found in many filamentous plant fungal pathogens [43, 44]. Regions of low G:C (AT-rich) content [45], which may be associated with RIP, were not observed in high proportions in the nuclear genomes of M4 or BFP (Additional file 8).

### The mitochondrial genome

The M4 mitochondrial genome (mtDNA) was assembled into a single molecule of 183 kb. The mitochondrial contig of BFP (NCBI accession DS231662) was downloaded and annotated. As LAGLIDADG endonucleases are known to have invaded the mtDNA of many larger fungal genomes [46, 47], M4 and BFP were searched and both were found to be enriched with intronic LAGLIDADG endonuclease genes and fragments; the endonucleases alone covered 50.7 kb (28%) of M4 mtDNA. Furthermore M4 mtDNA was 26 kb larger than BFP due to large duplicated regions (Additional file 9).

Gene order and copy number for whole and partial genes also differed between M4 and BFP mtDNAs. M4 had an extra duplicated set of two small subunit rRNA genes as compared to BFP. Also M4, like BFP and the closely related Pleosporales species *Parastagonospora nodorum* [48], only contained 13 of the 15 protein-coding genes common to fungal mtDNAs, lacking ATP synthase genes *atp8* and *atp9*. The M4 *cytochrome b* (*cob*) gene spanned 12 kb with mtDNA intron sizes between 0.3 and 3.8 kb. A comparison of the *cob* in silico translated protein sequences between *Pa. nodorum* (NC_009746), *Pyrenophora teres* f. sp. *teres* (Ptt), BFP (partial) and M4, confirmed that M4 did not have the G143A fungicide resistance mutation [49, 50], or the other known mutations G137R and F129 L [51]. However, sites of sequence variation were detected between these species, in particular F129 T. Also, the M4 *cob* did not have an intron at G143 and hence would be subject to QoI resistance (Additional file 10).

### Sequencing and assembly of additional Ptr isolates

An additional set of seven pathogenic Ptr isolates, representing races 1, 2, 5 and a new race were sequenced and assembled (Table 1). These comprised four Australian race 1 isolates (134, 239, 5213 and 11,137), a Canadian race 2 isolate (86-124) and two isolates from the USA, one belonging to race 5 (DW5) and the other belonging to a new race (AR CrossB10) [20, 22]. These genomes have been deposited at DDBJ/ENA/GenBank under the accessions MVBF00000000-MVBI00000000, MUXC00000000, NRDI00000000 and NQWZ00000000.

In addition, raw Illumina paired reads from the previously published non-pathogenic race 4 isolate (SD20) and race 5 isolate (DW7) [32], were also re-assembled in this study. The genome assembly sizes for all the additional isolates ranged from 33.0 to 34.6 Mb (Table 1). As the majority of collapsed contigs (higher read assembly depth) in Illumina genome assemblies tended to be the repetitive content, all the isolates were considered acceptable for gene analysis and the Illumina assembly for M4 was included as a control in the whole genome phylogenetic analyses (Additional file 11).

### Ptr whole genome phylogenetic analysis

**Fig. 2:**
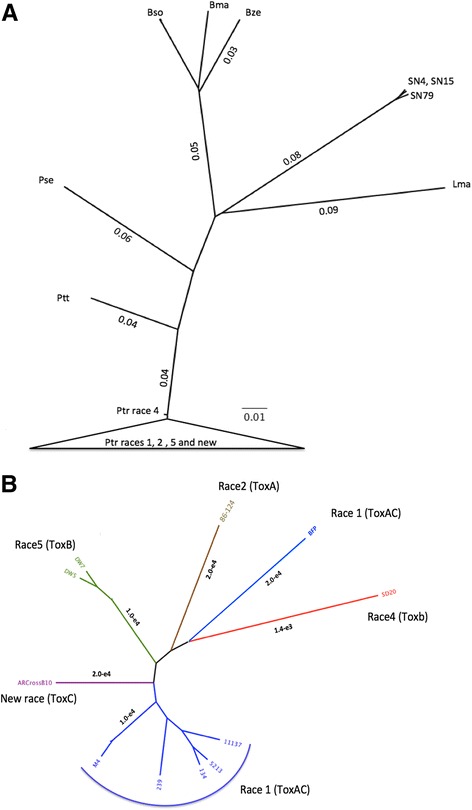
Whole genome phylogenetic analysis. **a** Whole genome phylogenetic tree shows raw branch lengths on a radial cladogram for *Pyrenophora tritici-repentis* (Ptr), *Pyrenophora teres teres* (Ptt), *Pyrenophora seminiperda* (Pse), *Bipolaris sorokiniana* (Bso), *B. maydis* (Bma), *B. zeicola* (Bze), *Parastagonospora nodorum* (ToxA isolates Sn4 and SN15 and non-pathogenic Sn79) and *Leptosphaeria maculans* (Lma). Ptr isolates are represented by race 1 (134, 239, 11,137, 5213, M4 and BFP), race 2 (86-124), race 5 (DW5 and DW7), a new race (AR CrossB10) and non-pathogenic race 4 (SD20). **b** Ptr whole genome phylogenetic tree shows raw branch lengths on a transformed radial cladogram for SD20 (race 4 red), 86-124 (race 2 brown), DW5 and DW7 (race 5 green), AR CrossB10 (new race purple), and M4, 134, 239, 5213, 11,137 and BFP (race 1 dark blue)

The assembled genomes for Pleosporales Ptt, *P. seminiperda* (Pse), *Bipolaris maydis* (Bma), *Bipolaris zeicola* (Bze), *Leptosphaeria maculans* (Lma) were downloaded from NCBI except *Pa. nodorum* (Sn4, SN15, Sn79) [52, 53]. The raw reads for *Bipolaris sorokiniana* (Bso) ToxA isolates [54] were downloaded from SRA, assembled for analysis. All the Ptr isolates formed a distinct phylogenetic clade when compared to the other Pleosporales species (Fig. 2a). A whole genome protein based comparisons of M4 with related species from the order Pleosporales showed high protein conservation between Ptt and Pse with >90% sequence identity. In comparison to distant genomes Bma, Bso, Lma and *Pa. nodorum* (Sn15), protein synteny was closer to 80% sequence identity. Regions of mesosynteny could be seen between M4 and *Pa. nodorum*, and in several regions of *Cochliobolus heterostrophus* (Additional file 11). Within the *Pyrenophora* genus, segmental rearrangements could also be seen relative to *P. seminiperda*.

To measure the genomic divergence between the Ptr isolates, whole genome Jukes-Cantor Kr distances were calculated, and all Ptr isolates were closely related with distances ≤5.3 × 10^− 3^. The greatest divergence was between the non-pathogenic isolate race 4 SD20 and the other races (Fig. 2b). The North American race 5 isolates (DW5 and DW7) formed a distinct clade, as did the Australian race 1 isolates, but no distinction was found within the Australian race 1 isolates by geographic state or sampling year. The North American isolate AR CrossB10 appeared closer to the Australian race 1 isolates, than it did to the North American race 1 isolate BFP and race 2 isolate 86-124 (Fig. 2b). The current race classification was not supported by whole genome phylogenetic analysis, as the race 1 isolates did not form a distinct clade, and nor did the ToxA-containing isolates.

### Ptr gene predictions and annotations

Genes were predicted using repeat-masked genomes for all isolates, including a re-prediction of genes in the published isolate BFP (referred to as BFP gene prediction version 2) using consistent bioinformatic methods and tools. RNA-seq isolate specific data was included for evidence based gene calls for isolates 134, 239, 5213, 11,137, DW5 and M4. For isolates without RNA-Seq data (DW7, BFP, SD20, AR CrossB10 and 86-124) RNA-seq data was pooled. Only concordant RNA-seq read alignments were used for evidence based gene calls, which ranged between 74 and 81% of the data across all isolates, except for SD20, which had a low alignment rate of 58% (Table 1). The lower rate of alignment in SD20 indicates a greater sequence divergence as compared to the other isolates. Between 7000 and 9000 gene predictions were supported over the full length by RNA-seq data, the highest number belonged to race 2 isolate 86-124 and AR CrossB10. Proteomic extracellular and intercellular data sets were also generated for DW5, M4 and 11,137 isolates (Additional file 12). Proteomic data sets supported 1568, 1868 and 1661 predicted genes for M4, 11,137 and DW5 respectively (Table 2).

**Table 2:** Gene prediction statistics for *Pyrenophora tritici-repentis* isolates

Isolate	M4 PacBio	^a^BFPV1	BFPV2	134	239	5213	11137	86-124	AR CrossB10	DW5	DW7	SD20
Protein coding genes	13,797	12,171	11,969	11,072	11,239	11,142	11,034	12,525	12,086	10,880	10,908	10,715
Total length CDS (Mb)	18.22	16.40	17.46	15.82	15.96	15.91	15.86	15.82	15.49	15.51	15.25	13.85
^b^Genes (K) Supported by RNA-seq	7.8	^e^7.8	^e^8.3	7.1	7.1	7.1	7.2	^e^9.0	^e^9.0	7.0	^e^8.6	^e^7.1
% Complete ^f^BUSCO	92.1	93.5	93.7	94.3	94	93.7	94.5	88.6	88.7	92.2	91.1	74.4
^c^Predicted effectors	224	260	179	188	190	193	184	246	227	186	191	184
^d^Supported by proteogenomics	1568	N/A	N/A	N/A	N/A	N/A	1868	N/A	N/A	1681	N/A	N/A
Predicted effectors supported by proteogenomics	79	N/A	N/A	N/A	N/A	N/A	78	N/A	N/A	97	N/A	N/A

^a^GenBank version 1.0, ^b^No. of genes with RNA-seq support (100%). ^c^No. of genes with effector prediction (EffectorP score >= 0.5). ^d^No. Genes with proteogenomics support overlap on same strand. ^e^RNA-seq data pooled. ^f^Benchmarking Universal Single-Copy Orthologs (BUSCO)

A total of 13,797 protein-coding genes were predicted in M4, whereas SD20 had only 10,715 genes predicted (Table 2). BFP version 2 had fewer gene predictions but a longer total CDS length than BFP version 1 (Table 2) due to improved overlap of RNA-seq data [55]. The gene sets of all Ptr isolates had similar codon usage biases (Additional file 13).

### Ptr orthologous genes

The nucleotide gene sets for M4 and BFP v2 were compared for reciprocal best hits at ≥90% identity and ≥ 90% sequence coverage, which resulted in 9,238 M4 genes orthologous to 9,006 BFP genes (Additional file 14). M4 has a larger core gene number due to paralog expansions. A total of 58 BFP genes were absent in the M4 genome, and 50 of these were classified as conserved hypothetical or predicted proteins. The remaining eight BFP genes contained functional domains, including PTRG_12024, which encodes a polyketide synthase.

**Fig. 3:**
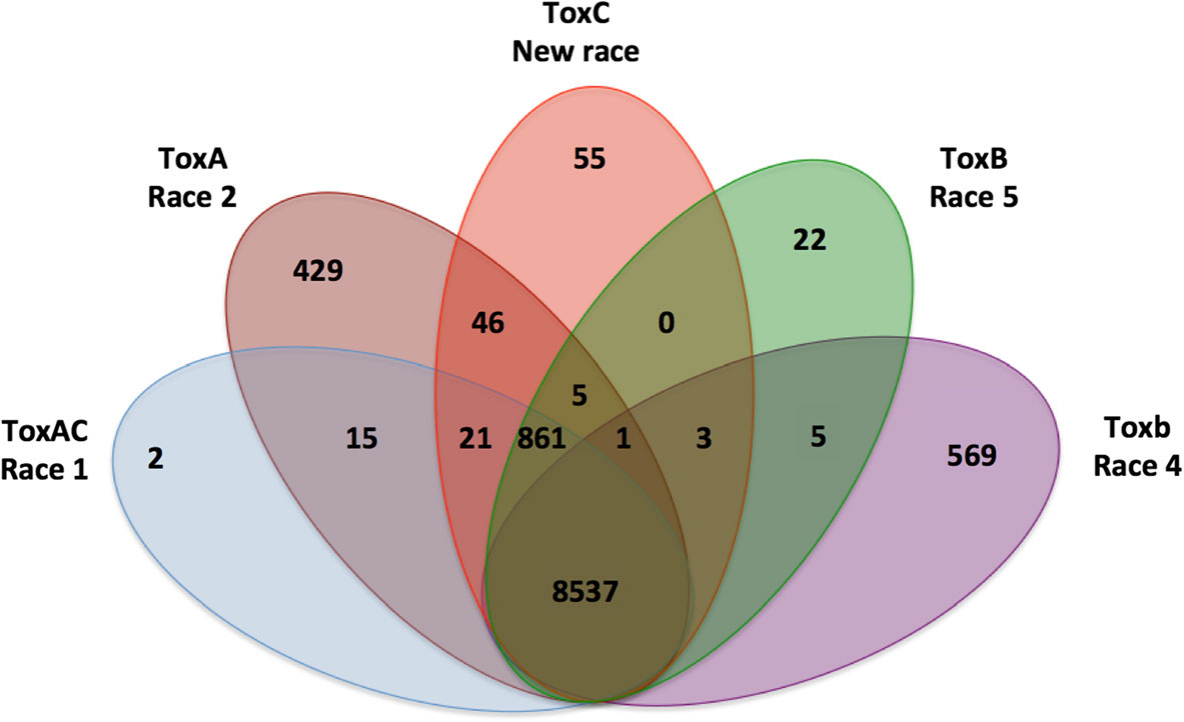
Ptr core orthologous protein groups. Venn diagram showing the number of orthologous protein groups that are core across the 11 Ptr genomes, and core to each race. Race 1 (ToxAC) is represented by six isolates (134, 239,11,137, 5213, M4 and BFP). Races 2 (ToxA), 4 (Toxb) and the new race (ToxC) are represented each by a single isolate (86-124, SD20 and AR CrossB10 respectively). Two isolates (DW5 and DW7) represent race 5 (ToxB)

A protein orthology analysis across the complete set of Ptr isolates was used to identify core and accessory isolate-specific and race-specific genes. Protein clustering assigned 98.6% of genes from all Ptr isolates to 12,476 orthologous groups (OGs) (Additional file 15). Across all isolates 8,537 groups (69%) were found in common (core) to all 11 isolates (Fig. 3). Orthologous groups unique to each race were also identified, however the number of groups core to a race decreased with the number of isolates tested.

When looking at unique core orthologous groups across races, 15 orthologous groups were found for ToxA-containing isolates (races 1 and 2) and not found in races 4, 5 and new race. These were comprised mainly of genes related to the *ToxA* horizontally transferred region [56]. However, 21 orthologous groups were core for races that produce necrosis symptoms (races 1, 2 and the ToxA-non-producing new race) and not found in races 4 and 5.

For ToxC isolates no genes were found uniquely core to just race 1 and the new race.

Of the 12,476 orthologous groups identified, 7,909 were single-copy gene orthogroups (63.4%). M4 had 468 multi-copy gene orthogroups, of which 161 groups were in common with BFP. This suggests different gene families have undergone expansion between the two isolates. The expanded BFP histone family [32] was found in all isolates and represented in 12 different orthologous groups for races 1, 2 and the new race, 11 orthogroups for race 5, and only seven orthogroups for race 4.

The core orthologous groups were screened for matching sequence lengths to calculate the pairwise rates of synonymous and non-synonymous changes relative to M4. A total of 1,941 genes had more non-synonymous changes than synonymous, and were considered to be under adaptive evolution (diversifying selection with a Ka/Ks >1). A further 2,573 genes had more synonymous changes than non-synonymous changes, and were therefore considered to be conserved genes (under purifying selection with a Ka/Ks < 1). The functional annotations of genes found under diversifying selection were enriched for the major facilitator gene superfamily (MFS) (enrichment score 26.21), as well as oxidoreductase activities involved with FAD-binding (enrichment score 7.92) and cytochrome P450 (enrichment score 7.54) (Additional file 16).

The genes of the new race AR CrossB10 that did not cluster with other Ptr isolate gene predictions, consisted mostly of unknowns and a transposase gene family (16 genes) that was closely related to Ptt.

### Effector genes

Genes with potential effector properties were predicted for all isolates (including the race 4 isolate SD20). Predicted effector gene numbers ranged between 179 and 260 based on an EffectorP score > 0.5 [57], (Table 2). Of the predicted effectors, extracellular proteins supported 42, 52 and 34% for 11,137, DW5 and M4 respectively (Table 2). Two potential novel candidate effectors unique to the new race were also identified, ARB10_4336 and ARB10_6921 with EffectorP [57] probability scores 0.962 and 0.787 respectively.

In M4, 16 candidate effector genes had effector probability scores ≥0.933, which included *M4_1392*, *M4_8650*, known effector *M4_1895* (*ToxA*) and a ‘multi-gene copy’ group *M4_1392*, *M4_5670*, and *M4_5670* (Table 3).

**Table 3:** M4 predicted effector genes with a high effector score (EffectorP score ≥ 0.933), BFP orthologues and detected in RNA-Seq data

M4 genes	^a^Equivalent BFP genes	^d^% Identity	^c^M4 RNA-Seq
M4_1392	PTRG_11346,	100	N
M4_5669	PTRG_11771,	100	N
M4_5670	PTRG_11773	100	N
M4_1895	ToxA	100	Y
M4_1920	PTRG_04901	100	N
M4_2488	^e^NA		Y
M4_3674	PTRG_01049	100	Y
M4_4087	PTRG_01437	100	Y
M4_4821	^e^NA		N
M4_8650	PTRG_01823	100	N
M4_9875	PTRG_08470	99.54	Y
M4_10891	^b^PTRG_06295	100	N
M4_11007	PTRG_07510	100	Y
M4_12104	PTRG_08870	100	Y
M4_13325	PTRG_09431	95.89	N
M4_13499	PTRG_09282	100	Y

^a^BFP Locus ID, ^b^Poor model match at locus, ^c^M4 mRNA evidence support for gene. ^d^Percentage Identity Blast global alignment between M4 and BFP genes. ^e^Gene sequence not identified in genome with greater than 90% identity and coverage (BLATX)

Ptr isolate genomes were also screened for orthologs of the 224-effector genes predicted for M4. Genes were counted as present if sequence identity was greater than 90% and covered 90% of the sequence. A total of 170 genes were found in all isolates and only 40 genes were absent in the isolate SD20 (Additional file 17). Of the homologous candidate effector gene groups tested, 23 genes were under positive selection and therefore considered under adaptive selection and 32 under negative selection under suggestive of conservation (Additional file 18).

The M4 *ToxA* gene was identified on Ch6 (M4 contig 1B: 1,564,333–1,565,556 bp), approximately 3 kb downstream of a large AT-rich region (13 kb) (Additional file 19), the result of RIP machinery [56]. As expected, the *ToxA* gene was identified in all race 1 and 2 isolates (134, 239, 11,137, 5213, BFP and 86-124). *ToxA*-like orthologs were not identified in the race 4 and 5 isolates (SD20, DW5 and DW7) or in AR CrossB10, while *ToxB* was only found in race 5 (DW5) and *toxb* in race 4 (SD20).

A comparison of the M4 and BFP *ToxA*-flanking region revealed three large deletion sites in M4 with a total size of 16 kb. A 5.4 kb Gypsy LTR deletion was identified downstream of *ToxA*, which suggests that these LTR elements are sites of recent transposon activity and potential recombination and insertion. The largest M4 deleted region (6.9 kb) occurred within the AT-rich region upstream from the *ToxA* gene. This region in BFP carried a hypothetical gene *PTRG_04888* (Additional file 19).

### Comparative analysis of the ToxA horizontally transferred region

Isolate genomes were aligned to M4 to identify regions of presence and absence across the different races (Additional file 20). The large *ToxA* containing region (LTC) found in BFP (145 kb) was also present in all race1 and race 2 isolates, but was absent from races 4, 5 and the new race (Additional file 20).

**Fig. 4:**
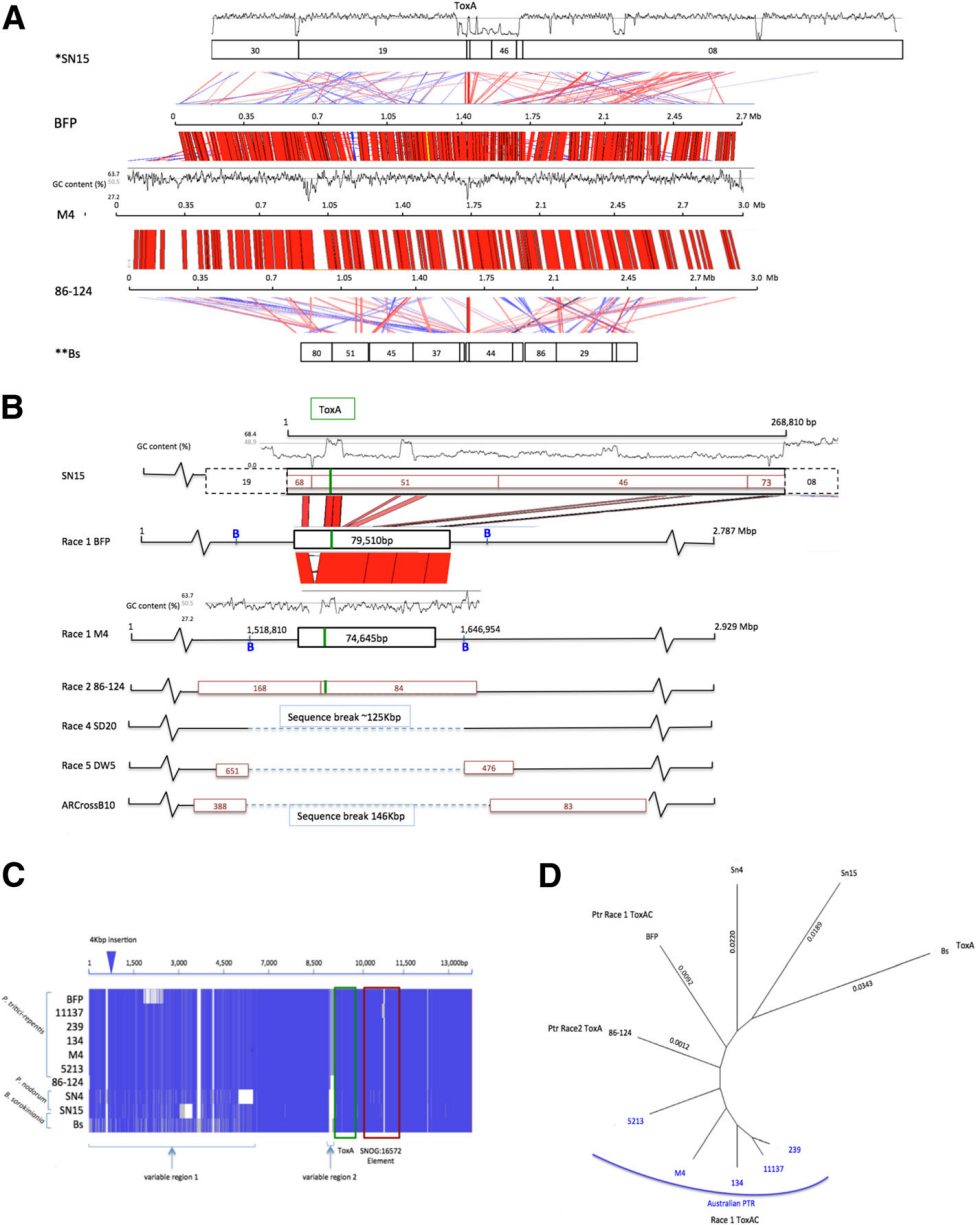
Analysis of the *ToxA* effector region of Ptr isolates and other Pleosporales species. **a** A large-scale nucleotide sequence alignment of *ToxA*-flanking regions (length ≥ 2000 bp) is plotted for (top to bottom) *Pa. nodorum* (Sn15), *P. tritici-repentis* BFP (NCBI accession DS231618), M4 (Ch6), 86-124 (C169 reversed and C85), and *B. sorokiniana* (Bs). The GC content from 0 to 100% is plotted in a 1 kb window for M4 and SN15 to show AT-rich regions. Large regions of mesosynteny flank the central co-linear region containing *ToxA*. Alignments in the same orientation are red, and reverse complemented alignments are blue. * SN15 scaffold order: 30, 19, 68 (reversed), 51, 46 (reversed), 73 (reversed), 8 (reversed). ** Bs scaffold order: 80, 51, 45, 37, 224, 278, 44, 136, 86, 29, 215, 117. **b** Mid-scale nucleotide sequence alignment of *ToxA*-flanking regions between *Pa. nodorum* (SN15), Ptr isolates (BFP, M4, 86-124, SD20, DW5 and AR CrossB10) and *B. sorokiniana* (Bs). A large deletion in Ptr races 4, 5 and the new race is represented by a blue dotted line. The LTC sequence breakpoints locations in BFP and M4 (represented by the letter B in blue) are displayed. Conserved blocks of co-linear sequence containing *ToxA* are represented by black boxes. The relative position of the *ToxA* locus is shown in green. **c** Nucleotide multiple sequence alignment of the highly conserved 14 kb co-linear region containing the *ToxA* locus in seven *P. tritici-repentis* isolates (BFP (DS231618: 1,433,261–1,435,189, 1,442,208–1,453,406), 11,137 (C11: 5087–64,267), 239 (C28: 89,064–102,896), 134 (C27: 89,083–102,915), M4 (C1B: 1,555,340–1,569,173), 5213 (C15: 50,499–64,331), 86-124 (C169: c0–8.619, C85: c84711–89800), two *Pa. nodorum* isolates Sn4 (C2085: 2559–3207, C1970: 1–3780, C1503: 1–7085, C2213: 793–2502) and Sn15 (scaf68: 3025–6288, scaf51: 4470–14,377) and *B. sorokiniania* (Bs) (C278: 1–13,886). Blue shading and white spaces show alignment identity and absence respectively. Two variable regions are shown upstream of the *ToxA* locus (yellow) and neighbouring SNOG16572 transposase element (red). **d** Neighbour joining tree of (**c**). DNA branch distances show a race 1 clade of the Australian isolates (blue)

A taxonomically wider analysis of synteny across the *ToxA* region was expanded to include other Pleosporales species known to contain *ToxA* homologs, namely, *B. sorokiniana* and *Pa. nodorum*. To better visualise synteny across the different species and Ptr isolates, whole chromosome regions were aligned in a pairwise manner (Fig. 4a). A large-scale pairwise alignment was performed between M4 Ch6 and the equivalent regions in BFP, *Pa. nodorum* SN15, *B. sorokiniana* and 86-124. Ptr isolates M4, 86-124 and BFP were found to be highly co-linear, while SN15 and *B. sorokiniana* had a small co-linear conserved *ToxA* region that was flanked by mesosyntenic regions (Fig. 4a). A large 300 kb low GC sequence region in SN15 had a high identity, co-linear alignment to a smaller 79 kb region in Ptr, the proposed result of a horizontal transfer from SN15 to Ptr. This co-linear region was flanked entirely by mesosyntenic regions of alignment [52, 58]. The large AT-rich regions upstream of *ToxA* could be observed between *Pa. nodorum* and Ptr.

The regions of co-linear alignment identified between the *ToxA*-containing species (Fig. 4a) were extracted to align to other non-*ToxA* producing Ptr isolates (Fig. 4b). In the co-linear region between Ptr and SN15, a 7 kb BFP and SN15 insertion can be observed upstream of *ToxA* (Fig. 4b). The Ptr LTC region [32] was found in all race 1 and race 2 *ToxA* isolates, but was absent from non-*ToxA* Ptr races 4, 5 and the new race (Fig. 4b). The conserved Ptr co-linear region (79 kb) between races 1 and 2 is flanked nearly 35 kb upstream and 8.5 kb downstream by the LTC region sequence breakpoints for Ptr race 4 and 5 (Fig. 4b). Only a single Bs contig of 14 kb was found collinear and used to extract the highly conserved (>95%) 14 kb co-linear sequences from Ptr and Pa for a multiple sequence alignment. This alignment showed two variable sequence regions 4 kb and 200 bp upstream of *ToxA* (Fig. 4c). Furthermore, the *ToxA* downstream neighbouring transposase element found in SN15 (SNOG16572) was also highly conserved across the different species. A neighbour joining phylogenetic tree based on this region grouped the Australian Ptr isolates closely (Fig. 4d), and the North American race 1 more distant than race 2 (Fig. 4d) which supported the whole genome phylogenetic analysis.

### Secondary metabolite gene clusters

The highly contiguous genome of M4 was searched for candidate secondary metabolite synthesis gene clusters. A total of 28 gene clusters for non-ribosomal peptide synthetases (NRPS) and polyketide synthetases (PKS) were identified in M4 across eight of the ten chromosomes. Clusters were comprised of 7 NRPS, 4 NPRS-like, 13 PKS and 4 PKS-like (Additional file 21). Four had a predicted core metabolite structure and a single type1 PKS gene cluster had ≥80% gene similarity to an *alternapyrone* biosynthetic gene cluster (MIBiG number BGC0000012). The gene cluster RNA-seq support and the presence of clusters in non-ToxC races 4 and 5 isolate genomes are shown in Additional file 21, to identify potential candidate gene clusters for ToxC.

## Discussion

### A high quality chromosome-level reference genome

This study presents the first PacBio-based, two enzyme optically mapped high quality reference genome assembly for a Ptr isolate. The M4 assembly resolved over 700 gaps when compared to the published genome of BFP [32]. The advent of single-molecule real-time (SMRT) sequencing technologies, such as PacBio [33], have transformed the assembly of whole fungal genomes, where near complete sequenced linkage groups for chromosomes up to 3 Mb in length were produced for M4. This is a significant improvement over the fragmented assemblies produced from earlier sequencing technologies. The M4 PacBio assembly contigs were essentially the same size as the scaffolds produced for BFP. The M4 assembly traversed genomic repetitive content and produce contiguous sequences that better represented whole chromosomes. Indeed, the majority of currently available filamentous fungal genomes are fragmented scaffolded assemblies that could be improved to better identify genomic variations.

The M4 contiguous assembly includes previously unresolved sequence regions in Ptr. These regions included long complex features such as telomeres, centromeres, repetitive regions, co-regulated genes clusters, structural rearrangements, and high AT/GC compartments. Furthermore, by incorporating the BioNanno two-enzyme hybrid mapping approach, 98% of M4 sequence was allocated to chromosomal positions. These new resolved regions serve to improve our understanding of potential sources of pathogenicity loci and the prediction of novel effectors that would not have been previously possible. Prior to this study the only Ptr genomes available were from North America. The M4 high quality genome is a new and relevant global resource representing the southern hemisphere, supported by transcriptomics and proteogenomics data. Furthermore, M4 is an important research resource, also supported by an extensive catalogue of specific phenotypic, detection data [18, 59] and mutants incapacitated in *ToxA* [17].

### Ptr chromosome structural rearrangements includes a whole chromosome fusion

The genomes of filamentous ascomycete fungi are generally highly plastic in both sequence and order [58]. Comparative genomics between M4 and BFP revealed major structural variations, which included insertions, deletions, inversions, segmental rearrangements and chromosome fusion. This was supported by a previous study on Ptr population genetics that observed significant differences between North American and Australian isolates [60].

Many of the large genome structural variations observed in M4 coincided with scaffold breaks in the BFP equivalent regions. A proposed mechanism for the structural rearrangements observed between nuclear genomes are telomere-centromere associated breakage-fusion-bridge cycles, which together with large beds of LTR insertions have been proposed to contribute to a rapid evolution of accessory chromosomes and other regions of genome innovation [61]. This extensive chromosomal reshuffling is a mechanism that can drive evolution of virulence in pathogens [62] and over time produces a mesosyntenic conservation pattern [41]. Our analysis indicated that the segmental duplications and LTR repeats in M4 were observed at telomeres, and LTR digest analysis showed that the majority of elements only contained polypurine tracts and were devoid of internal gag or pol genes suggesting a long period of LTR inactivity [63]. The large LTR regions associated with genome wide segmental duplications in the telomeres and centromere regions support a telomere-associated chromosome breakage and translocation model, as well as the potential re-integration or fusion of chromosomes as seen for M4 Ch10. The large repeats in the distal telomeres, as seen in M4 Ch1, are potential chromosomal breakage points. Further potential break points in M4 Ch3 also coincided with beds of LTRs and repeats. It has been has been previously suggested in *L. maculans* that peri-centromeric clusters of retrotransposons are potential fusion points for chromosomes [39, 64], it is therefore possible that the RIP degenerated LTRs observed in M4 are possibly points of fusion and breakage. Furthermore, structural volatility was also seen in the mitochondrial genome (mtDNA) of M4. M4 mtDNA displayed variations with BFP in length, structure, and gene order/number. A high variability of mitochondrial gene order among fungi has been previously reported [65]. In this study the observed structural rearrangements and increase in gene copy numbers of M4 mtDNA are the likely result of the invasive disruptions by intergenic and intronic endonucleases.

Rapid mitochondria mutational changes are known to occur in response to fungicides, one such mutation occurs in the *cytochrome b* (*cob*) gene [66], whereby a G143 glycine substitution to an alanine imparts resistance to strobilurin fungicides [49, 50, 67]. Although strobilurin resistance in Ptr is present in Europe [50] the mutation was not found in our isolates, although they remain at risk, as a variation at the mutable F129 site was detected.

### Genome-level phylogeny of Ptr does not conform to the race concept

The concept of race or pathotype in plant pathology is widely used. Prior to the genomic era, such classifications were based on phenotypic variation on a set of differential host cultivars. The value of a race classification was that it identified the minimum number of isolates that encompassed the virulence spectrum of the pathogen species. With the advent of widespread genomic analyses of pathogen isolates, it has become possible to test whether races correspond to phylogenetic groups/clades.

The race structure of Ptr is based on the presence of ToxA (inducing necrosis), ToxB and ToxC (both inducing chlorosis) and hence eight races are defined. However, AR CrossB10 does not fit the eight-race classification as it has ToxC and lacks ToxA and ToxB, yet produces necrosis on the ToxA differential wheat variety Glenlea [21]. Also, the Race 1 isolates did not cluster (Fig. 2).

A new race classification based on ToxA, ToxB and ToxC combined with broad symptoms of chlorosis and necrosis symptoms is proposed. We suggest that the current Ptr race system be expanded to include symptoms of necrosis (Nec) and chlorosis (Chl) observed on any wheat line (not just limited to differentials) in addition to presence/absence of the three Ptr effectors (Table 4). Accordingly, AR CrossB10 would be categorised as a new race (race 9), due to observable symptoms of necrosis and chlorosis (Nec^+^Chl^+^) coupled with the presence of ToxC (and absence of ToxA and ToxB). This system is flexible enough to encompass additional unclassified isolates as they are discovered, and the table can be simply extended to include new effectors once identified. Confounding the race concept is the limited set of tan spot differential wheat cultivars defined by Lamari and Bernier [68]. These lines are not typical of the variety of germplasm grown commercially around the world, and indeed some may be restricting the discovery of new effectors and races. Thus, there is a need to accordingly replace and expand the differential wheat lines to include more representative cultivars.

**Table 4:** A proposed new Ptr race classification system incorporates the presence/absence of disease symptoms in conjunction with presence/absence of known Ptr effectors

Effectors	Disease Symptoms			
	Nec+Chl+	Nec−Chl−	Nec+Chl−	Nec−Chl+
AC	Race 1			
A			Race 2	
C	AR CrossB10			Race 3
–		Race 4		
B				Race 5
BC				Race 6
AB	Race 7			
ABC	Race 8			

A = ToxA, B = ToxB, C = ToxC, Nec = Necrosis, Chl = Chlorosis, − = absence of symptom, + = presence of symptom

The effector *ToxA* gene is ubiquitously found in the Australian Ptr population [69]. The suggested horizontal transfer of *ToxA* from *Pa. nodorum* to Ptr is proposed to have occurred in the 1940s, so it is a relatively young disease of wheat [56]. Tan spot was first documented in Australia in the 1950s, when leaf lesions on wheat were reported in New South Wales and Queensland [70]. In this study, five race 1 Australian isolates were collected between 1987 and 2009 and sequenced to identify if genetic variations or genomic drift under differing environmental pressures had occurred since Ptr was introduced to Australia. These were also compared to isolates from Canada and the USA in order to test the current Ptr race classification.

Our whole genome phylogenetic analysis showed that the *ToxA*-containing isolates from different countries did not cluster together. Greater genomic variation was observed between the Australian race 1 isolates and the North American race 1 isolate, than between the Australian race 1 isolates and races 2, 5 and the new race. An apparent closer distance between the non-ToxA producing AR CrossB10 to the Australian race 1 isolates indicates that there are additional underlying genomic features beyond effectors that may contribute to race distance. This is in agreement with previous population studies, which were unable to find a correlation between race and geographic origin [23, 24, 28, 29].

The Australian race 1 isolates formed a distinct clade, which is consistent with arrival of a single race 1 isolate and low diversification after arriving in Australia. Likewise, this close relationship between the Australian race 1 isolates was also evident in the *ToxA* region phylogenetic analysis.

Traditionally, the term ‘race’ encompasses regional divergence and adaption to local conditions. Given the young age of tan spot disease, the traditional measure of genetic change may not apply yet. Moreover, Ptr is able to undergo sexual reproduction on wheat stubble, which coupled with the adoption of minimal tillage practises and limited crop rotations, may contribute to the occurrence of genetic variability independent of race or geographic origin. Altogether, these results showed that a more extensive phylogenetic analysis encompassing genomes of all races is required.

### Ptr core and accessory gene contents

The prediction and annotation of Ptr gene sets for each isolate was conducted using consistent methods to ensure biases were not introduced to the analysis based on differing approaches. In this study, RNA-Seq data was integrated into our high throughput gene predictions, as evidence-based support for each isolate. The final gene numbers supported by RNA-seq were consistent for most of the isolates except for race 2 and the new race (AR CrossB10), which had a slightly higher number of genes supported. This may be caused by the expansion of specific gene families compared the other isolates. Based on this finding the RNA-seq data generated in this study is a valuable resource to support gene predictions for this species. Furthermore, proteogenomics data also aided in the validation of specific gene models and is a useful resource to support the identification of extracelluar and intracellular proteins in Ptr.

Orthologous gene inference is a fundamental step in comparative genomics, in our protein orthologous analysis a significant proportion of genes were variable across this species. Further, for genes found core to all isolates a larger proportion were found to be under diversifying selection and enriched for functions related to a major membrane transporter.

### Identifying effector genes

Ptr may have many effectors that are yet to be characterised and the prediction of such effectors can be often missed in genome annotations. The prediction of novel effector genes can be difficult, as small-secreted effector proteins appear to be unrelated by sequence similarity, but can be grouped into structurally conserved families. This makes identifying new putative candidates based only on sequence similarity difficult [71]. Previous search methods for fungal effectors were based on small cysteine-rich secreted peptides with signal peptides. However, methods have more recently emerged utilising properties other than homology for their prediction [57]. The molecular function of these effector genes and the evolutionary mechanisms that generated them are mostly unknown [71], but at least some are likely to be capable of lipid membrane association and/or translocation [72–75]. In our study we found that candidate effectors could be predicted in both pathogenic and non-pathogenic isolates and a core Ptr candidate gene set was identified. Furthermore, the presence and absence of accessory candidate effectors varied between isolates. In our search for novel necrosis genes, two candidates were identified in AR CrossB10 unique to this new race. There are many potential sites of variation between pathogenic and non-pathogenic isolates to help identify effector genes (such as ToxC). In this study a number of secondary metabolite clusters were identified, but when screened against non-ToxC producing isolates none of these appeared to be candidates. The ToxC candidates are therefore still elusive and reliable metabolomics-based methods of detection are required to facilitate identification.

### The ToxA horizontal transfer event(s)

Previous studies have revealed insights into the potential evolutionary history of *ToxA* and the surrounding region. *ToxA* was initially thought to be present only in Ptr and the wheat pathogen *Pa. nodorum*. This was based on a 11 kb homologous ToxA-containing region that was suggested to have been horizontally transferred from *Pa. nodorum* to Ptr [56]. This was later redefined as a much larger 300 kb region in *Pa. nodorum* based on a comparison with BFP [52]. More recently, the barley and wheat pathogen *B. sorokiniana* was reported to contain a 12 kb region homologous to the *ToxA* locus and flanking sequences, which is nearly identical to the 11 kb region common to both Ptr and *Pa. nodorum*. This *ToxA* region was found in one third of the Australian *B. sorokiniana* isolate population examined [54]. In our study of the *ToxA* region, a 79 kb highly conserved homologous co-linear region, horizontal transfer region (HTR), was found common to Ptr and *Pa. nodorum*. In Ptr, the HTR was further contained in a larger 145 kb *ToxA*-containing region (LTC) in all Ptr race 1 and 2 isolates. The LTC recombination breakpoints detected between the Ptr races may be the boundaries of a genetic region introduced by a horizontal transfer event [76, 77], however such an event is likely to be associated with transposable elements (TEs). Based on our genomic analysis of the region no TEs were observed to be significantly associated with the LTC sequence breakpoints. Nevertheless, flanking Gypsy LTR elements did exhibit presence/absence variation at flanking sites, indicating elements at such sites can be deleted (Additional file 19: Figure A). In Ptr, the maintenance of sequence continuity at both breakpoints and the lack of insertion elements at these sites suggest that the integration of foreign DNA may have occurred by homologous recombination at sites that are no longer in the genome. In Ptr races 1 and 2, the *ToxA* locus and associated transposase element were highly conserved (Fig. 4c), however additional copies of the associated element were identified not associated with any predicted effector genes but in regions of high repeats, that appear to be ancient sites of genome invasion.

The single horizontal *ToxA* region transfer to Ptr appears, based on our set of genomes, to be stably integrated. Although it is possible that frequent horizontal transfer events could undermine the concept of Ptr genomic lineage and result in incoherent phylogenetic histories [78] the HTR phylogeny supported the whole genome phylogenetic analysis. Indeed repeated genomic transfers between co-circulating populations may eventually drive divergence [79]. Population genetics/genomics coupled with phenotype data will provide an opportunity to identify trends in genetic diversity that may represent increased pathogenicity.

## Conclusions

This study contributes eight new Ptr isolate genomes, integrated with transcriptomics and proteogenomics data, which has supported the reliable prediction of novel effector candidates. Our key findings are: 1) Ptr genome structure displays large scale rearrangements and chromosome fusions between isolates; 2) Whole genome phylogeny did not support Ptr race classification; 3) Race 1 Australian isolates displayed low genomic divergence; 4) A significant portion of genes are variable between races; 5) Orthologous protein clustering identified many candidates of potential novel function, which included potential novel effectors; 6) The phylogenetic analysis of the Ptr *ToxA*-containing region supported the whole genome phylogeny; 7) Ptr acquisition of *ToxA* involved potentially two genomic events, a 79 kb horizontal transfer region (HTR) and a larger region ranging between 129 and 145 kb in size (LTC), as supported by race 1 and 2 isolates. Uncovering new contributing effectors is important in understanding pathogenicity, which is expected to impact the agriculture industry, particularly through the future isolation of new effectors and commercial adoption of effector-resistant cultivars.

Representing a genomic ‘snapshot’ of tan spot in Australian wheat-growing regions and a new pathogen type to the US, we anticipate that these genomic resources will be invaluable to understanding host-pathogen co-evolution and the genetic basis of pathogenicity.

## Methods

### Isolate collection and sequencing

The pathogenic race 1 isolate M4 was isolated from tan spot infected wheat leaves collected from Meckering, Western Australia in 2009. Australian isolate 134 was obtained from Biloela, Queensland in 2001; 239 from Sealake, Victoria in 2003; 5213 from Billa Billa, Queensland in 2001; 11,137 from Yuna, Western Australia in 2001.

All Australian isolate genomic DNA was extracted from mycelia grown in vitro. Genomic DNA of M4 was extracted using the BioSprint 15 DNA Plant Kit (Qiagen, Hilden, Germany) with some modifications [80]. Briefly, DNA was extracted from 3-day old mycelia grown in Fries three medium using the BioSprint 15 automated workstation according to the manufacturer’s instruction. DNA was further treated with 50 μg/ml of RNase enzyme (Qiagen, Hilden, Germany) for 1 h followed by phenol/chloroform extraction. DNA was then precipitated with sodium acetate and ethanol, and resuspended in TE buffer.

The M4 genome was sequenced using PacBio SMRTcell technology at 75X and quality checked by the NGS service at McGill University, Canada. The collection protocol was MagBead OneCellPerWell v1 and the run concentration was 100 pM. Six Pacbio SMRT cells yielded approximately 3.5 Gbp of long reads, with an average read length of 15 kb.

For M4, 134, 239 and 5213, the Australian Genome Research Facility (AGRF) generated Illumina paired-end reads of 100 bp. The complete isolate DNA extraction, library preparation and sequencing information can be found in the BPA metadata store (https://downloads.bioplatforms.com/wheat_pathogens/). Isolates 11,137 (WAC11137) [81] and DW5 were sequenced using Illumina paired-end reads of 100 bp via the Australia National University and Macrogen Inc. (Korea), respectively, as per submission guidelines (https://dna.macrogen.com/eng/support/ngs/guide/ngs_sample_submission.jsp). These six isolate bio-samples are registered under NCBI BioProject, PRJNA315205.

For isolates AR Cross B10 and 86-124, Novogene (https://en.novogene.com) sequenced Illumina HiSeq paired-end 150 bp reads. Fresh mycelia tissue was sent directly to Novogene for DNA extraction and sequencing.

Additional sequence reads were downloaded from NIH SRA for DW7 (SRR4026621 and SRR4026622) and SD20 (SRR06572) for assembly and annotation.

RNA extraction, library preparation and sequencing information are available at https://downloads.bioplatforms.com/wheat_pathogens_transcript/.

### Whole-genome assembly

The PacBio SMRT sequence data for the M4 isolate was error corrected using the long filtered read (LFR) and subread cyclic consensus sequences (CCS) data sets using PacBio’s HGAP error correction [82] and filtering with a minimum read quality of 0.75, minimum subread length of 500 bp and coverage cutoff of 30× (https://github.com/PacificBiosciences/pbdagcon). Alignments were performed using Blasr version (1.3.1.142244) [83] with minimum read length of 200 bp and reads skipped that had a length less than a maximum score of − 1000 across their full length.

Over 750 Mb (18× genome coverage) error-corrected long reads were then used for assembly. Sequence data was checked with FASTQC [84] to check length distribution. The corrected reads were assembled using Canu-1.0 [85] on a Nectar NC Linux-amd64 machine resource with default parameters. M4 genomic Illumina short pair end reads were quality checked and trimmed for high quality sequence with Trimmomatic v0.22 [86], head crop 6 bp and minimum length 50 bp. The paired-end reads were aligned to the PacBio assembly with Bowtie2 [87] and paired-end reads with concordant alignments were selected with SAMtools view 0.1.19-96b5f2294a [88] for error correction. A final genome assembly error correction (polish) was conducted using Pilon [35] version 1.16 (Additional file 22).

Whole genome assembly sequence and gene annotation was publicly available for Ptr isolate BFP (Genbank accession AAXI01000000). Sequence reads for DW7 (SRR4026621 and SRR4026622) and SD20 (SRR06572) were downloaded from the NCBI SRA for assembly. Illumina genomic sequence data for isolates was quality checked with FASTQC [84], trimmed for poor quality, ambiguous bases, and adapters using CutAdapt v1.8.1 (-q 20 -u 12 -m 50 -n 2) and Trimmomatic v0.22 [86] head crop 6 bp and minimum length 50 bp. De novo assembly was completed with Velvet Optimiser v2.2.4 (-s 59 -e 65 -× 2 -f ' -fastq -shortPaired -k 'n50' -c 'Lbp' -o ' -exp_cov 100 -cov_cutoff 10 -max_coverage 100 ' -a yes -z 0.4) [89] and SPAdes (version v3.10.0).

### Optical mapping

High-molecular-weight (HMW) DNA was extracted from M4 protoplast as fungal protoplast agarose plugs were prepared as described by Ellwood et al. [90] with some modifications. Briefly, mycelia were harvested from 7-day V8PDA agar culture and inoculated into 250 ml Erlenmeyer flask containing 80 ml of Fries medium [91]. A total of 480 ml fungal cultures were grown at room temperature with constant shaking at 100 rpm. After 3 days, fungal cultures were blended in a Waring blender and an equal volume of Fries three medium was added to the cultures, followed by further incubation for 18 h under the same growth conditions. Protoplasts were prepared as described previously [17]. Protoplasts were then collected by filtering through five layers of miracloth, washed two times in mycelial wash solution (MWS) and resuspended to a final concentration of 2 × 10^9^ protoplasts/ml. Protoplasts were embedded in agarose by adding 45 μl of 2% clean cut agarose (Bio-Rad Laboratories, Hercules, CA, USA) to 75 μl of protoplasts suspension. Solidified agarose plugs were incubated with 2 mg/ml of proteinase K enzyme (Qiagen, Hilden, Germany) in 2.5 ml lysis buffer (10 mM Tris pH 8.0, 1% N-lauroyl sarcosine, 0.2% sodium deoxycholate and 100 mM EDTA pH 8.0) at 50 °C with gently agitation for 18 h. The plugs were washed four times in wash buffer (20 mM Tris pH 8.0 and 500 mM EDTA pH 8.0) and treated with 80 μg/ml RNase enzyme (Qiagen, Hilden, Germany) for 2 h. Plugs were then washed four times in wash buffer and stored in TE buffer.

In silico nicking density was estimated for nicking enzymes Nb.BbcCl and Nt.BspQI using Bionano Label Density Calculator™, target label density was 15/100 and 18/100 kb respectively. Bionano Irys™ tiff files were converted to BNX files using Autodect. AssembleIrysCluster™ with in-silico maps and adjusted BNX files were de novo assembled to give 100× molecule coverage. Consensus genome maps were then aligned to the *in silco* maps using RefAligner and stitched using XMAPSs [92]. Maps were visualized in the Bionano Access™ (Additional file 1).

In silico maps for nicking enzymes Nb.BbcCl were also produced for BFP (NCBI genome accession AAXI01000000) and aligned using RefAligner to the M4 Nb.BbcCl optical map for comparative analysis (Additional file 23).

### Transcriptome sequencing and alignment

Wheat cultivar Mace was infected with M4 (*in planta*), RNA collected from wheat leaves samples three and 4 days post infection, were sequenced by Ramaciotti Centre for Genomics (https://www.ramaciotti.unsw.edu.au/). The *complete* isolate RNA extraction, library preparation and sequencing information can be found in the BPA metadata store (https://downloads.bioplatforms.com/wheat_pathogens_transcript/).

In vivo RNA samples were sequenced at the Australian Genome Research Facility (AGRF). Stranded Illumina RNA-Seq 100 bp pair-end reads were generated for each isolate: 134, 239, 11,137, 5213, DW5 and M4, to provide evidence for gene predictions. Spliced alignment to genomes were conducted with Bowtie2-build v2.1.0.0 [93] and TopHat v2.0.12 (-N 0 -i 10 -I 5000 -p 16 --no-discordant --no-mixed --report-secondary-alignments --microexon-search --library-type fr-firststrand) [94] respectively, and transcripts assembled with Cufflinks (-p 16 --library-type fr-firststrand --overlap-radius 10 --min-intron-length 10 --max-intron-length 5000) [95].

### Proteogenomics

Fungal in vitro protein samples of M4, 11,137 and DW5 were sourced from in vitro cultures, and processed by the Queensland Institute of Medical Research (QIMR) Protein Discovery Centre (QPDC).

Extracellular proteins were obtained from Fries three liquid culture filtrates. Culture filtrate was sequentially filtered through gauze, miracloth and Whatman filter paper using vacuum filtration followed by passing through a 0.2 μm filter and was kept on ice until further processing [17]. The culture filtrate (CF) was dialysed for 48 h using SnakeSkin® pleated dialysis tubing (BioRad) with a molecular weight cut-off (MWCO) of 3.5 kDa in deionised water at 4 °C. One part of culture filtrate was mixed with four parts of chilled TCA/acetone solution and incubated for 3 h at − 20 °C, prior to centrifugation for 15 mins at 4 °C and 3220 *rcf*. The supernatant was discarded and the pellets were resuspended in a total of 10 ml of chilled 100% acetone. The suspension was incubated for 48 h at − 20 °C followed by three washes of acetone/Tris. The washed pellet was air-dried at room temperature and resuspended in 20 mM Tris pH 7. Residual TCA was removed by dialysis using D-Tube™ Dialyzer Maxi, MWCO 3.5 kDa (Novagen, Merck KGaA, Darmstadt, Germany) in 20 mM Tris pH 7 at 4 °C for 48 h, followed by centrifugation of the dialysed suspension for 15 mins at 4 °C and 20,238 *rcf*.

Intracellular samples were obtained from 3-day in vitro cultures grown in minimal media at 100 rpm and 27 °C. Mycelia were snap-frozen in liquid nitrogen and lyophilized overnight in a freeze-drier. The freeze-dried mycelia were ground into a fine powder in liquid nitrogen using a chilled mortar and pestle and proteins were solubilised in 10 mM Tris-Cl pH 7, before centrifugation at 20238 *rcf* and 4 °C for 15 mins. The supernatant containing soluble proteins was desalted using a PD10 chromatography column (GE healthcare) according to the manufacturer’s protocol.

All protein samples were BCA quantified and run on a 16.5% tris/tricine gel to check for integrity.

Intracellular protein samples were concentrated via TCA/acetone protein precipitation.

All samples were separated into 24 fractions by OFFGEL electrophoresis (IEF). Proteins of each fraction were alkylated (iodoacetamide) and trypsin-digested. Peptides of each fraction were separated on a C18 column in an LC run and analysed by high-resolution mass spectrometry (LTQ Orbitrap Velos or XL).

Spectra were searched against database of six-frame translated genome, a reverse decoy peptide database and contaminant sequences of keratin and trypsin using Peppy™ (2015) [96]: maximumFDR 0.01; precursorTolerance 2000; fragmentTolerance 300; Digestion rules - cleavageAcid R, cleavageAcid K, numberOfMissedCleavages 1; static mods -modC 57.021464, variable modifications Ox(M), Deamidation (N,Q), fixed modifications Carbamidomethyl (C), peptide tolerance of 20 ppm, MSMS tolerance of 0.8 Da, trypsin, one missed cleavages and maximum FDR threshold 0.01.

### Repetitive DNA prediction and masking

Ptr isolate genomes were masked for low complexity, as well as known transposable elements using RepeatMasker (RM) [97] version open-4.0.6 with rmblastn version 2.2.27+ on RepBase [38] RM database version 20,150,807 (taxon = fungi). De novo repeat families were identified using RepeatScout version 1.0.5 [98]. LTR regions were identified using GenomeTools, Suffixerator, LTRHarvest (GenomeTools) v1.5.1 (minimum distance 200 bp and maximum distance 5000) and LTRDigest (GenomeTools) v1.5.1 [39].

De novo repeat families were searched for RIP-like SNP mutations [42] via RIPCAL v2.0 [42, 99]. Repeat family mutation sites were plotted for the highest mutation rates CpA- > TpA per kb. Segmental duplication in M4 was determined based on Nucmer alignments greater than 90% identity and greater than 5000 bp; segmental duplication genome coverage was determined using BEDTools coverage v2.17.0.

Repeat coordinates were plotted to the M4 genome using CIRCOS version 0.69-3 [100].

### Gene prediction and functional annotation

Final gene calls were performed for all isolates by the same workflow method (Additional file 24) for comparative analysis. Ab-initio gene predictions were made with GeneMark-ES v 4.33 (--ES --fungus --cores 16) [101] and Codon Quarry v1.2 [55] assisted by RNA-Seq alignments. In order to capture highly polymorphic genes, Ptr BFP reference proteins [32] were aligned using Exonerate v2.2.0 (--showvulgar no --showalignment no --minintron 10 --maxintron 3000) mode protein2genome [102]. The final gene prediction sets were predicted via EvidenceModeller v1.1.1 [103] using a combination of supporting protein and transcript alignments and ab initio predictions on a repeat masked genome, with minimum intron length of 10 bp and weights for ab initio prediction CodingQuarry:1, GeneMark.hmm:1, BFP protein exonerate:2.

Gene predictions for the M4 and BFP mitochondrial genomes were made using Mitos webserver [104] and refined based on blast versus http://mitofun.biol.uoa.gr/ protein sequences. Circular visualisation of mitochondrial features was generated using GenomeVx web application (March 2016) [105]. The *dothideomyceta* mitochondrial apt8 and apt9 Mycosphaerella graminicola genes were retrieved from Genbank NC_010222.1:c34402-34256 and NC_010222.1:c23405-23181 and searched using Blat [106] at 30% nucleotide identity against all *Pleosporaceae* genomes (NCBI *Taxonomy ID*: 28556).

The cob sequences were detected in genomes using Exonerate minimum intron 10 bp, maximum intron 5000 bp and protein2genome model. Protein sequences were extracted with EMBOSS 6.6.0.0 extractseq, and transeq code table 3. Protein sequences were then aligned using Muscle [107, 108]. Sequences downloaded from GenBank were *Zymoseptoria tritici* (GenBank accessionAAP81933), Ptt (DQ919067.1: <1–18, DQ919067.1: 3150–3326, DQ919067.1: 4485–4499, DQ919067.1: 5818–>5853), Ptr (GenBank DQ919068.1: <1–12, DQ919068.1: 2648–2692, DQ919068.1: 5575– >5607), SN15 (NC_009746: 7571–8072, 9093–9817), BFP (DS231662.1: 12,445–12,644, 14,994–15,178, 19,016–19,066, 2147–21,999), Ptt (NW_00352501: c1382–1559, 4712–4911 and NW_00356055: 1418–1737, 2045–2393). Other sequences included, 11137_00499, 134 _00534, 239_00547, 5213_00542, 86-124_00253, AR CrossB10_00129, DW5_00657, M4_00017: c26166-26485, 26,793–27,141, 29,386–29,438, 32,324–32,374, 36,212–36,396, 38,746–38,945), sn4_contig_1698, sn79_contig_2277.

Gene annotations were assigned from BLASTX (v2.2.26) [109] searches against KEGG (August 21, 2007), Uniref90 (Jan 28, 2016), NCBI Refseq (taxon = Ascomycota) (Jan 27, 2016) and InterProScan version 5.17-56 [110]. Sequence domains were assigned by RPS-BLAST (v2.2.26) against Pfam (Oct 31, 2012), Smart (Oct 31, 2012) and CDD (Oct 31, 2012). The blast protein and domain searches were then summarised using AutoFACT version 3.4 [111]. Gene Ontology (GO) functional assignment was conducted using DAVID [112] to level 1 and tests for enrichment were performed based on an ease score threshold 0.1, against a background of *Pyrenophora tritici-repentis* BFP proteins. Functional annotations and potential interactions were also searched using StringDB [113].

The M4 genome was searched for secondary metabolite clusters using Smurf [114] and Fungi-SMASH, antibiotics and Secondary Metabolite Analysis Shell, version 4.0 [115].

### Phylogenetic analysis

Mulitple sequence alignments of Protein/DNA were made with ClustalW v2.1 [116]. Neighbour joining (NJ) trees were built from sequences using Phylip 1:3.695-1 [117] with 100 times bootstrapping for distances and consensus tree using Phylip (seqboot, protdist/dnadist and consense). Phylogenetic trees were viewed in JalView [118] and FigTree v1.4.3 (https://github.com/rambaut/figtree).

The *ToxA* co-linear regions were extracted using EMBOSS v6.6.0.0 extractseq, aligned with ClustalW v2.1 [116]. A neighbour joining (NJ) tree was built using Phylip 1:3.695-1 [117] with 1000 times bootstrapping for distances. Phylogeny trees were rooted mid-point and cladogram transformed using FigTree v1.4.3 (https://github.com/rambaut/figtree). Raw branch lengths are displayed. Alignment between the maize-infecting *B. maydis* and M4 was highly mesosyntenic, and the *ToxA* locus had a low translated peptide identity of 38% and was not included in this analysis.

### Comparative genomics

Whole genome alignments were based on NUCmer v3.1 (--maxmatch –coords) and displayed using Artemis Comparison Tool (ACT) v13.0.0 with a GC window size 1000 bp and alignments filtered by score length of 2000 bp for visualisation.

Orthologous protein clustering of predicted protein sequences was conducted with OrthoFinder version 1.1.4 [119] at an expected value 0.001. A Venn diagram of protein orthologous groups was constructed with JVenn [120].

Gene functional annotation (e.g. GO and InterPro terms) and enrichment was found for orthologous groups using StringDB [113] and DAVID Bioinformatics resource [112, 121, 122].

Genome data for BFP (https://www.ncbi.nlm.nih.gov/nuccore/AAXI00000000.1) was downloaded from GenBank [32]. The BFP scaffold sequences were assembled into chromosomes from the AGP file available at the Broad Institute http://www.broadinstitute.org/ftp/pub/annotation/fungi/pyrenophora/genomes/pyrenophora_tritici-repentis_1/. Other Pleosporales (*Taxonomy ID*: 92860) genomes were downloaded from NCBI GenBank Genome division for comparative analysis, *Bipolaris maydis* ATCC 48331 (https://www.ncbi.nlm.nih.gov/genome/2586), *Bipolaris zeicola* (https://www.ncbi.nlm.nih.gov/genome/13436), *Pyrenophora seminiperda* (https://www.ncbi.nlm.nih.gov/genome/16916), *Pyrenophora teres f. teres* 0–1 (https://www.ncbi.nlm.nih.gov/genome/2995). The *Parastagonospora nodorum* Sn4, SN15 and Sn79 genomes were obtained through https://github.com/robsyme/Parastagonospora_nodorum_SN4, https://github.com/robsyme/Parastagonospora_nodorum_SN15 and https://github.com/robsyme/Parastagonospora_nodorum_SN79 [53].

*Bipolaris sorokiniana* genome SRR4434051, SRR4434052 and SRR4434053 were downloaded from the NCBI SRA; quality processed using Skewer (version 0.2.2) and assembled with SPAdes (version v3.10.0) for analysis. The DW7 and SD20 gene sequences are available in Additional file 25.

Genome alignments were based on NUCmer v3.1 (--maxmatch –coords) and displayed using Artemis Comparison Tool (ACT) v13.0.0 with a GC window size 1000 bp and alignments filtered by score length of 2000 bp for visualisation.

Sequences alignments between M4 and BFP chromosomes greater than 5 kb were also visualised with SyntenyMiner version 0.001 https://sourceforge.net/projects/syntenyminer/.

Illumina reads were aligned to the M4 genome using BWA version 0.7.5a-r405 [123], and PacBio reads with BWA-SW v0.7.14-r1138 long query read, mismatch penalty 5, gap open penalty 2, gap extension penalty 1, Z-best 10. Alignments were viewed in IGV [124], Tablet version 1.16.09.06 [125] and SAMtools Tview version 0.1.19-96b5f2294a [88].

Genome nucleotide pairwise distance was calculated with GenomeTools version 1.5.8 genomediff [126]. Andi [127], with Kimura modelling and anchor pair significance of 0.05 was also used for calculating genomic distances and 1000-time bootstrap. Whole genome phylogenetic trees were constructed using Phylip 1:3.695-1 [117] Consensus tree program v3.695 on 1000 Fitch-Magoliash (F-M) v3.695 trees with power 2.0. Trees were transformed cladogram with using FigTree v1.4.3 (https://github.com/rambaut/figtree). Raw branch lengths are displayed.

Alignments of whole genome assemblies of all isolates were made versus the M4 reference with MUMmer (maximal matches,) using both NUCmer v3.1 and PROmer v3.07 (BLOSUM45) [128]. Multiple Isolate genome alignments were visualised in Mauve version snapshot 2015-02025 and Progressive Mauve alignment [129]. Genome features and read alignments were visualised with the Integrative Genome Viewer (IGV) version 2.3.55 [124]. Alignment coordinates were visualised in CIRCOS version 0.69-3 [100] M4 genome GC content was calculated for a window size of 1000 bp using BEDTools v2.17.0 makewindows and nucBed [130]. Isolate average read coverage was calculated over a window size of 1000 bp using SAMTools bedcov v0.1.19 [88] and BEDTools genomecov v2.17.0 [130].

The AT richness of genomes was assessed using Occultercut –v1.1 [45].

Isolated gene predictions, transcripts and reference GFF3 file comparisons were made with BEDTools version 2.17.0 intersect [130, 131].

Core orthologous gene groups from OrthoFinder analysis were identified across 11 Ptr isolates. The pairwise rates of synonymous and non-synonymous changes were then calculated for each isolate compared to the reference M4 isolate with the KaKs calculator v2.0 [132] (MA = Model Averaging), and with a Fisher’s exact test *p*-value ≤0.05 [132]. Functional annotation cluster analysis was conducted on GO terms and InterPro terms for genes with a dN/dS ratio >1, using DAVID version 6.7 [112] with default settings and *Pyrenophora tritici-repentis* BFP as the background.

The SN15 SNOG_16572 (XM_001806616.1) transposase gene (associated with *ToxA*) was searched against unmasked genome data sets using BLATX [106] and minimum protein identity of 30%. Alignments were then filtered at 70% protein identity for higher identity reporting.

## Additional files

Additional file 1: Optical mapping supporting data. Page 1. M4 two enzyme optical mapping (Bionano Irys System) workflow, with second round of de novo assembly with adjusted values and hybrid scaffolding. Page 2. Tables of M4 restriction enzyme nicking densities and enzyme molecule fragment statistics. Page 3. An example image of nicked and fluorescently labeled long DNA strand molecules for restriction enzyme Nt.BbvC1. Pages 4–13. Final M4 Optical Map showing M4 in silico digested contig and two enzyme optical map alignments. Page 14. Optical Map resolution of M4 contig1: Figure shows M4 contig1 (C1 7 Mb) in silico two enzyme digest map (centre orange) and restriction site alignment to enzyme Nt.BbvC1 optical maps (top purple) and Nt.BspQ1 enzyme optical maps (bottom yellow). The contig1 assembly site not confirmed by the two enzyme optical maps is displayed at the 4 Mb region. (PDF 2614 kb)

Additional file 2: PCR validation of M4 PacBio pre-optical map assembly. A) Table of PCR results to validate M4 PacBio genome regions. B) Three PCR gel results show primer results for Ptr isolates M4 (M), DW5 (D) and negative no template control (C). The amplified product bands are shown for M4 contig 1, 3, 6, 9 and 17. C) Pre-optical mapM4 contig alignments to BFP chromosomes are shown at ≥90% identity and ≥ 5 Kbps in length. M4 contigs are displayed above alignments and BFP chromosomes below. Red connecting lines represent sequence alignments in the same orientation between M4 and BFP sequences, and reverse-complemented alignments are blue. Grey markers indicate distal ends of contigs with identifiable telomere motifs. Regions validated by PCR in M4 are indicated in green on contig 1, contig 3, contig 6 and contig 9. (PDF 631 kb)

Additional file 3: Repeat content plot for M4 genome. Circos plot displays repeat and gene content for M4 genome (contigs 1–15). Displayed in order is a heat map of GC content (red is high AT content), gene frequency over a 100Kbp window, repeat frequency (100Kbp window), and positions of LTR, segmental duplications and histones genes. Major repeat regions are found in contig distal locations and associated with high LTR content. (PDF 322 kb)

Additional file 4: M4 and BFP RepBase known repeat element summary. (XLSX 36 kb)

Additional file 5: List of M4 de novo repeats and domains. (XLSX 29 kb)

Additional file 6: M4 plot of large segmental duplications. Circos plot displays M4 genome LTR positions and segmental duplications (SD) greater than 5 kb and 90% nucleotide identity between contig 1 and the rest of the genome (contigs 1–15), inter-contig (blue links) and intra-contig (red links). Intra-contig links are shown mainly between the telomeres and centromere of contig 1. (PDF 394 kb)

Additional file 7: RIPCAL2 summary for M4 RIP analysis. (XLSX 216 kb)

Additional file 8: *Pyrenophora* genome AT/GC composition plots. *Pyrenophora* genome AT/GC composition plots, minus the mitochondrial genome. Plotted genomes are *Pyrenophora tritici-repentis* M4 and Pt-1CBFP, *Pyrenophora semeniperda* (Psem) and *Pyrenophora teres f. teres* (Ptt). Only Psem displays a bimodal plot of GC composition (blue). (PDF 40 kb)

Additional file 9: M4 and BFP Mitochondrial analysis. A) M4 Mitochondrial contig 17 self-plot shows two events of inverted duplication. The first 13 kb of the mitochondrial contig has an inverted duplication at 50–63 kb and the last 13 kb has an inverted duplication at 80–93 kb (resulting in an extra two copies of small ribosomal RNAs). This is not a typical pattern for confirming circularisation. B) Dotplot of M4 versus BFP mitochondrial contigs. C) M4 Mitochondrial genome (183Kb) and D) BFP (157Kb) are shown left and right respectively. Mapped to the outer ring are protein-coding genes and ribosomal RNA, the inner ring shows the positions of the endonucleases and transfer RNAs. (PDF 608 kb)

Additional file 10: Mitochondrial *cob* gene analysis. A) M4 mitochondrial *cob* gene spans 12 kb (26–39 kb) with intron spans greater than 2 kb. B) Mitochondrial cytochrome b protein multiple sequence alignment for *Zymoseptoria tritici* (AAP81933), Ptt (DQ919067.1), Ptr (GenBank DQ919068), SN79_contig_2277, SN4_contig_1698, SN15 (NC_009746), 11,137_00499, 134 _00534, 5213_00542, 86-124_00253, AR CrossB10_00129, DW5_00657, M4_00017, Ptt (NW_00352501 and 00356055), and BFP (DS231662.1) shows three known mutation sites for fungicide resistance. (PDF 102 kb)

Additional file 11: Genome sequence plots of M4 compared to other isolate fungi. Page 1. Protein sequence plots of M4 (vertical axis) against necrotrophic fungi *Parastagonospora nodorum* (Sn15), *L. maculans*, *P. teres f. teres*, *P. seminiperda*, *B.maydis* isolates ATCC48331 and C5 (Teleomorph *Cochliobolus heterostrophus*) *B. zeicola* and *B. sororkiniania (*Bsoro*)* scaffolds (horizontal axes) show good alignment protein conservation to M4. Page 2. Genome sequence plots of M4 compared to other Ptr isolate contigs. Page 3. Whole genome phylogeny of Ptr isolates including M4 Illumina assembly. (PDF 1243 kb)

Additional file 12: Proteogenomics extracellular and intracellular data for Ptr race 1 isolates 11,137, DW5 and M4. (TXT 3140 kb)

Additional file 13: Ptr isolates codon usage radar plot. (PDF 601 kb)

Additional file 14: M4 and BFP highly conserved genes. M4 and BFP gene overlap at 90% sequence coverage and identity. M4 has a larger core gene number due to increase copy number of orthologous genes compared to BFP. (PDF 87 kb)

Additional file 15: Protein clustering of genes from all Ptr isolates orthologous groups. (TXT 3252 kb)

Additional file 16: Enrichment scores for functional annotations of orthologous groups under diversifying selection. (XLSX 62 kb)

Additional file 17: Predicted effector homolog gene counts in Ptr. Heatmap shows M4 predicted effectors (Effectorp probability score >= 0.5) homologue counts. Gene sequences were searched at 90% identity and coverage (BLATX) against all Ptr isolates genomes. (PDF 41 kb)

Additional file 18: Predicted effector homologous genes non-synonymous and synonymous changes. (XLSX 47 kb)

Additional file 19: M4 and BFP alignment of the *ToxA* region. A) Sequence plot for M4 contig1 *ToxA* 170Kb region (5.65-58 Mb) on the horizontal axis and BFP DS231618 170Kb region (1.36–1.55 Mb) on the vertical axis show a number of sequence variations and features in common. B) Sequence plot shows three major deletion sites in M4 and the AT-rich region upstream of the *ToxA* gene. C) Alignment between M4 and Pt-1C-BFP isolates show an alternate view of the three large insertions/deletions in the *ToxA* race 1 specific region (blue arrowed region above and below Fig) and distal flanking homologous sections (blue boxes connected by blue dotted line). The deletion positions in M4 contig1 are 5,685,572 bp, 5,724,456 bp, 5,799,713 bp which correspond to Pt-1C-BFP DS231618 4, 6.9 and 5.4 kb insertions respectively. The total length of M4 *ToxA* race 1 region is ~129Kb and Pt-1C-BFP is ~145bpkb (regions in common are shaded in pink). M4 genes are plotted between the two sequence similarity plots (light pink). (PDF 647 kb)

Additional file 20: Whole-genome overview of the gene and repeat features of the Australian M4 reference isolate, and comparisons to alternate Ptr isolate genomes. Whole-genome overview of the gene and repeat features of the Australian M4 reference isolate, and comparisons to alternate Ptr isolate genomes. A) The six outer labelled rings illustrate: M4 genome contigs 1–15; a heat map of M4 local GC content within 10 kb windows (low (AT-rich) = red); M4 repeat density within 100 kb windows (red); M4 gene density within 100 kb windows (blue); LTR content (red); and genome RIP indexed regions (purple). The eight inner rings show coverage of M4 genome contigs 1–15 by alignments of alternate Ptr isolates in windows of 100 kb. Australian race 1 isolates 134, 239, 111,137, and 5213 and North American race 1 BFP are indicated in blue. Race 2 86-124 and the new race AR CrossB10 are shown in brown and purple respectively. Race 5 DW5, DW7 are red and orange. Race 4 SD20 is green. The *ToxA* position is marked in contig 1. B) A higher resolution view of the *ToxA* region on M4 contig 1, labelled as per part A, showing presence of this region in race 1 isolates and absence of this region in race 4 and 5 isolates. (PDF 770 kb)

Additional file 21: M4 Predicted Secondary Metabolite PKS and NRPS gene clusters. (XLSX 39 kb)

Additional file 22: PacBio assembly methods and data. A) PacBio SMRT cell genome assembly flow chart overview showing input data (disc shapes) and tasks implemented (rectangles) from long read error correction and assembly through to final genome polishing (base error correction). B) PaBio SMRT cell M4 sample and sequencing statistics. (PDF 269 kb)

Additional file 23: M4 Optical map compared to BFP in silico maps. (PDF 5715 kb)

Additional file 24: Workflow and tools utilised for all isolate genome annotation. Workflow and tools utilised for all isolate genome annotation. Genome annotation flow chart overview shows input genome data (disc shape) and tasks implemented (rectangles) for protein and RNA-Seq alignments, three ab initio gene predictions for protein coding genes and predictions for non-coding rRNA and tRNA. (PDF 238 kb)

Additional file 25: Supporting sequence data for DW7 and SD20. Sequence data for DW7 (SRR4026621-2) and SD20 (SRR06572) assembled for analysis in this study. (TXT 9982 kb)
