# Correction to: Comparative genomics of the wheat fungal pathogen *Pyrenophora tritici-repentis* reveals chromosomal variations and genome plasticity

**DOI:** 10.1186/s12864-018-5059-1

**Published:** 2018-09-13

**Authors:** Paula Moolhuijzen, Pao Theen See, James K. Hane, Gongjun Shi, Zhaohui Liu, Richard P. Oliver, Caroline S. Moffat

**Affiliations:** 1 Centre for Crop Disease and Management, Department of Environment and Agriculture, Curtin University, Bentley, Western Australia Australia; 2 Department of Plant Pathology, North Dakota State University, Fargo, ND USA

## Correction

Following publication of the original article [1], the author flagged the following errors:

1. All occurrences of “11,137” should be “11137”. This is the name of an isolate.
2. URL link “https://downloads.bioplatforms.com/wheat_pathogens/” on page 15 is not working, please use “https://data.bioplatforms.com/organization/bpa-wheat-pathogens-genomes”
3. URL link “https://downloads.bioplatforms.com/wheat_pathogens_transcript” on page 16 is not working, please use “https://data.bioplatforms.com/organization/about/bpa-wheat-pathogens-transcript”
4. One occurrence of “In vivo” on page 16 should be “In vitro” and italicized.
5. One occurrence of “Codon Quarry v1.2” on page 17 should be “Coding Quarry v1.2”
6. One occurrence of “F129 L” has a space inserted on page 6 and should be “F129L”
7. One occurrence of “F129 T” has a space inserted on page 6 and should be “F129T”
