# Supplementary material for: Comparative genomics of the wheat fungal pathogen Pyrenophora tritici-repentis reveals chromosomal variations and genome plasticity
Source: BMC Genomics. 2018 Apr 23;19:279. doi: 10.1186/s12864-018-4680-3 (PMC5913888; doi:10.1186/s12864-018-4680-3)
Supplement: Supplementary file 1 — Optical mapping supporting data. Page 1. M4 two enzyme optical mapping (Bionano Irys System) workflow, with second round of de novo assembly with adjusted values and hybrid scaffolding. Page 2. Tables of M4 restriction enzyme nicking densities and enzyme molecule fragment statistics. Page 3. An example image of nicked and fluorescently labeled long DNA strand molecules for restriction enzyme Nt.BbvC1. Pages 4–13. Final M4 Optical Map showing M4 in silico digested contig and two enzyme optical map alignments. Page 14. Optical Map resolution of M4 contig1: Figure shows M4 contig1 (C1 7 Mb) in silico two enzyme digest map (centre orange) and restriction site alignment to enzyme Nt.BbvC1 optical maps (top purple) and Nt.BspQ1 enzyme optical maps (bottom yellow). The contig1 assembly site not confirmed by the two enzyme optical maps is displayed at the 4 Mb region. (PDF 2614 kb) [file 12864_2018_4680_MOESM1_ESM.pdf]

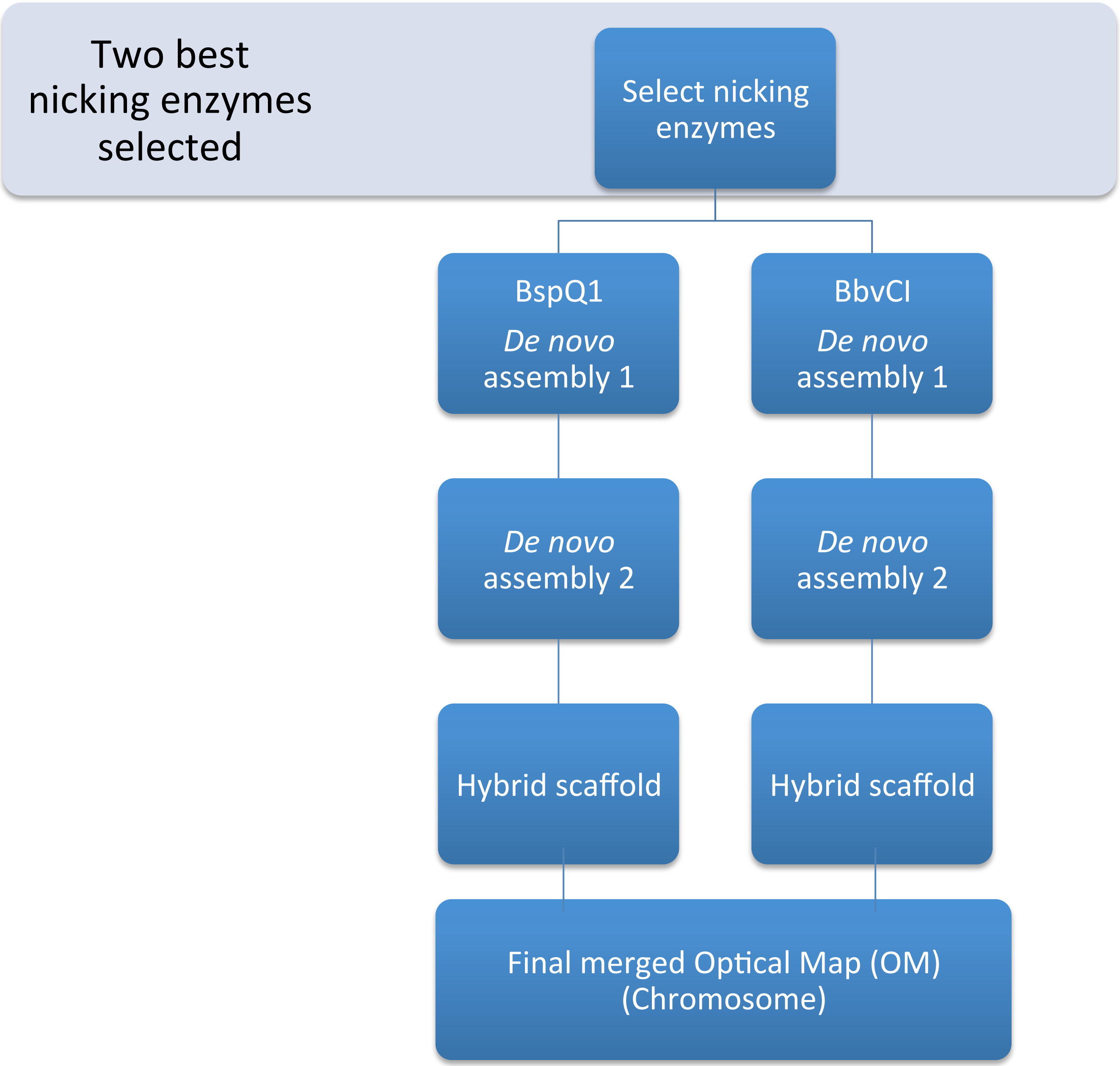

S1 Fig. Optical Map assembly (1 of 14 slides)

Table M4 restriction enzyme nicking density and labels per 100kb

Enzyme	NickDensity (Nicks/ 100kb)	LabelDensity (Labels/ 100kb)
Nt.BspQ1	26.17856	17.95897
Nb.Bsml	50.15073	27.85473
NbBbvCI	20.38771	14.86319
Nb.BsrD1	66.08656	32.1038
Nb.BssSI	43.95672	25.75829

Table M4 Restriction enzyme molecule fragment statistics

Enzyme	BbvC1	BspQ1
Min molecule length for assembly (kb)	150	150
Mapped Molecule Quantity (Mb)	9,949.13	76,661.32
Mapped Avg Size (Kb)	199	220
Avg Label Density (per 100 Kb)	13.9	14.9
Number of Consensus Genome Maps	44	76
Consensus Genome Maps Size (Mb)	41.332	68.999
Consensus Genome Maps N50 (Mb)	1.352	1.052
Avg Depth of Molecule Coverage	100.8	99.6

S1 Fig. Optical Mapping Statistics (2 of 14 slides)

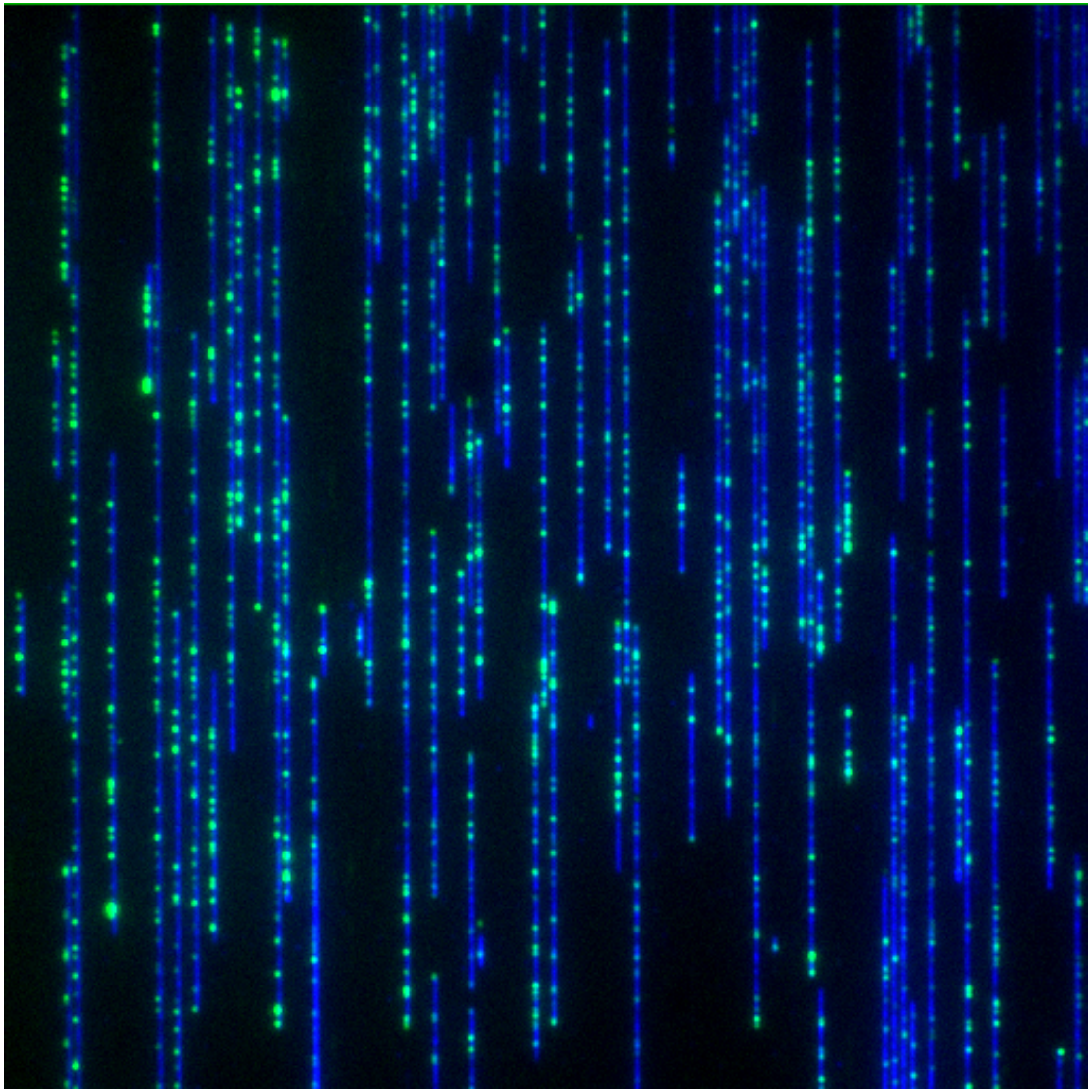

S1 Fig. Image of M4 Optical mapping (3 of 14 slides)

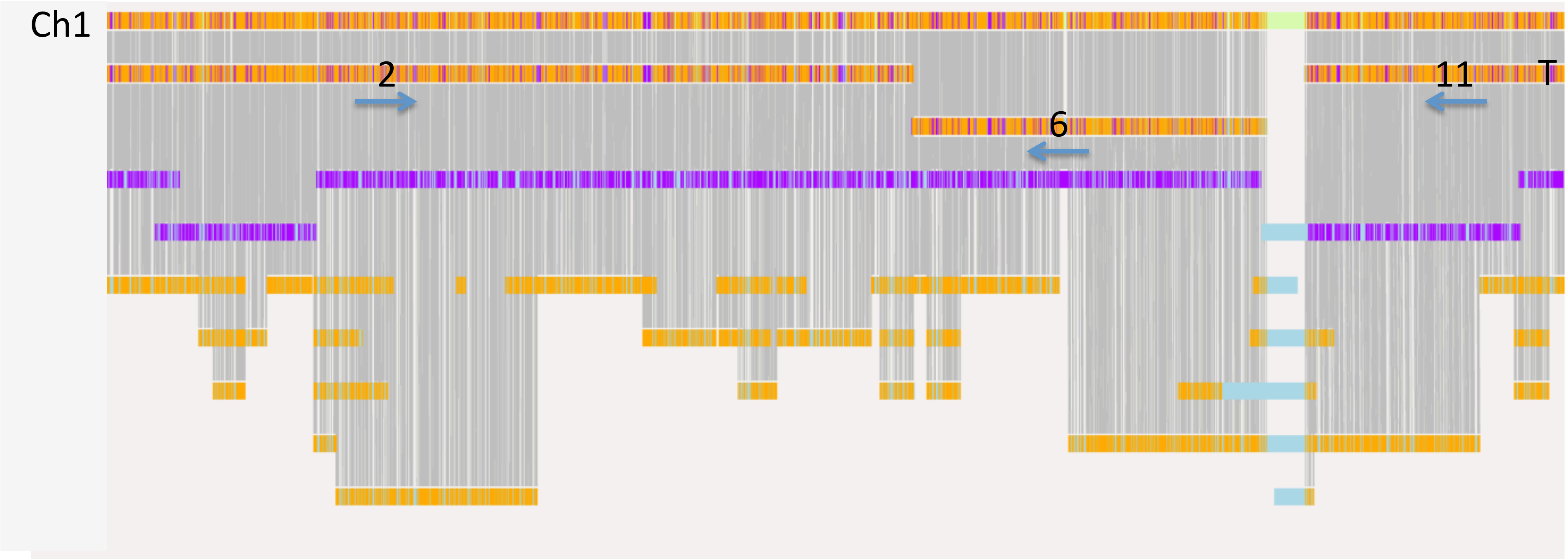

Figure shows the Final Optical Map of M4 Chr1 (H4) (10Mb) (top) and the alignment of 1) *in silico* digested M4 contigs (2, 6 & 11) (middle orange bars 2) M4 enzyme Nt.BbvC1 optical maps (purple) and 3) M4 enzyme Nt.BspQ1 optical maps (bottom yellow). Telomeres are also shown (T).

S1 Fig. M4 Optical Map for Chromosome 1 (4 of 14 slides)

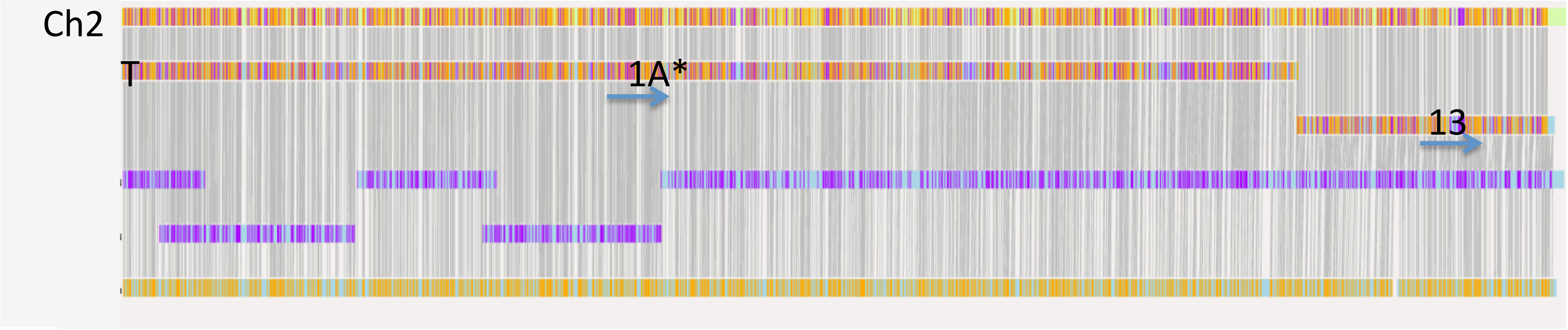

Figure shows the Final Optical Map of M4 Chr2 (H2) (5.1Mb) (top) and the alignment of 1) *in silico* digested M4 contigs 1A* and 13 (middle orange bars 2) M4 enzyme Nt.BbvC1 optical maps (purple) and 3) M4 enzyme Nt.BspQ1 optical maps (bottom yellow). Telomeres are also shown (T).

*Refer to slide 14

S1 Fig. M4 Optical Map for Chromosome 2 (5 of 14 slides)

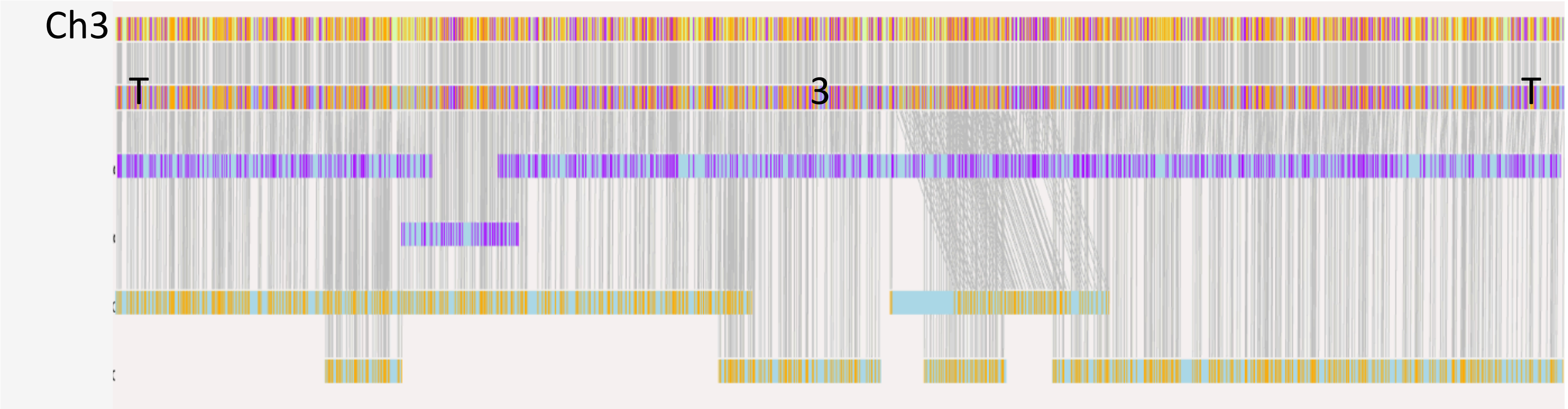

Figure shows the Final Optical Map of M4 Chr3 (H7) (3.6Mb) (top) and the alignment of 1) *in silico* digested M4 contig 3 (middle orange bars 2) M4 enzyme Nt.BbvC1 optical maps (purple) and 3) M4 enzyme Nt.BspQ1 optical maps (bottom yellow). Telomeres are also shown (T).

S1 Fig. M4 Optical Map for Chromosome 3 (6 of 14 slides)

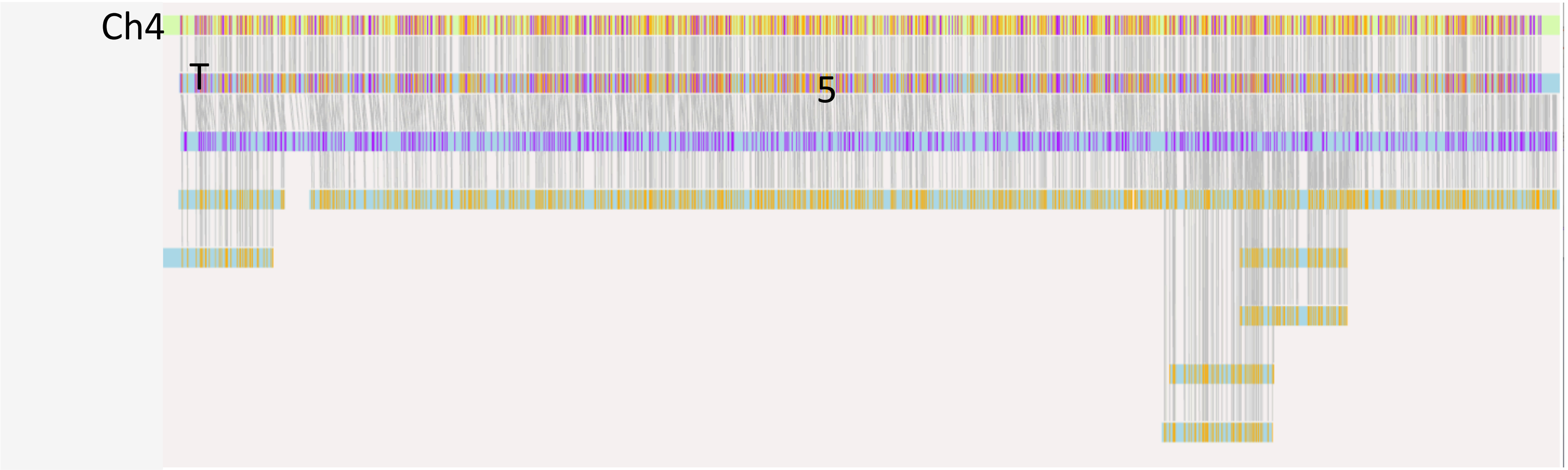

Figure shows the Final Optical Map of M4 Chr4 (H9) (3.1Mb) (top) and the alignment of 1) *in silico* digested M4 contig 5 (middle orange bars 2) M4 enzyme Nt.BbvC1 optical maps (purple) and 3) M4 enzyme Nt.BspQ1 optical maps (bottom yellow). Telomeres are also shown (T).

S1 Fig. M4 Optical Map for Chromosome 4 (7 of 14 slides)

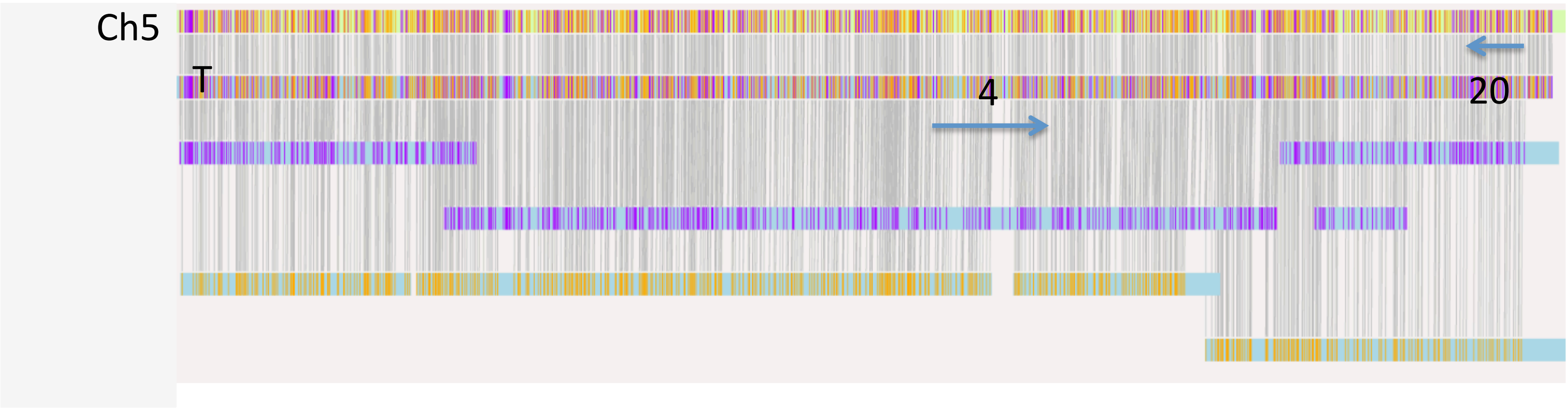

Figure shows the Final Optical Map of M4 Chr5 (H8) (3.4Mb) (top) and the alignment of 1) *in silico* digested M4 contigs 4 and 20 (middle orange bars 2) M4 enzyme Nt.BbvC1 optical maps (purple) and 3) M4 enzyme Nt.BspQ1 optical maps (bottom yellow). Telomeres are also shown (T).

S1 Fig. M4 Optical Map for Chromosome 5 (8 of 14 slides)

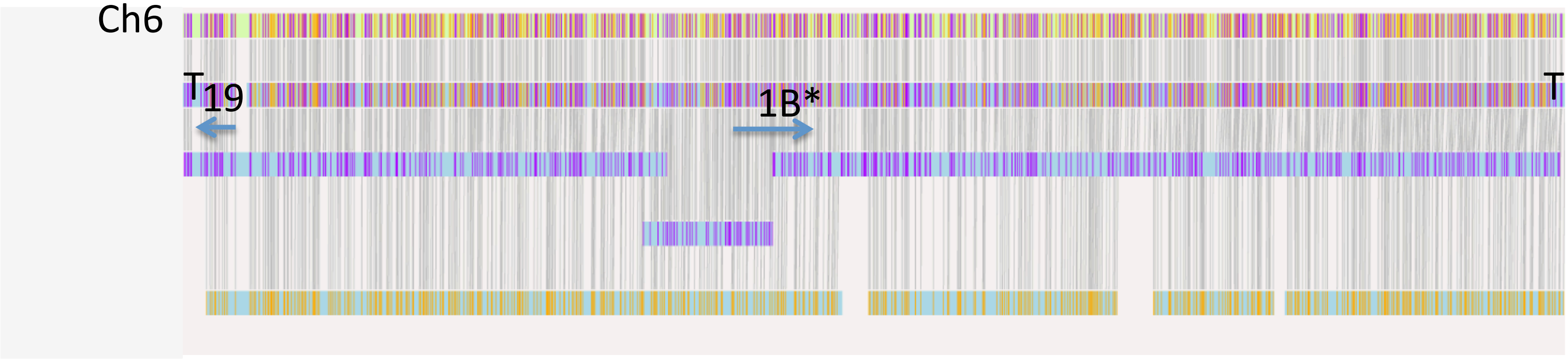

Figure shows the Final Optical Map of M4 Chr6 (H6) (3.0Mb) (top) and the alignment of 1) *in silico* digested M4 contigs 19 and 1B* (middle orange bars 2) M4 enzyme Nt.BbvC1 optical maps (purple) and 3) M4 enzyme Nt.BspQ1 optical maps (bottom yellow). Telomeres are also shown (T).

*Refer to slide 14

S1 Fig. M4 Optical Map for Chromosome 6 (9 of 14 slides)

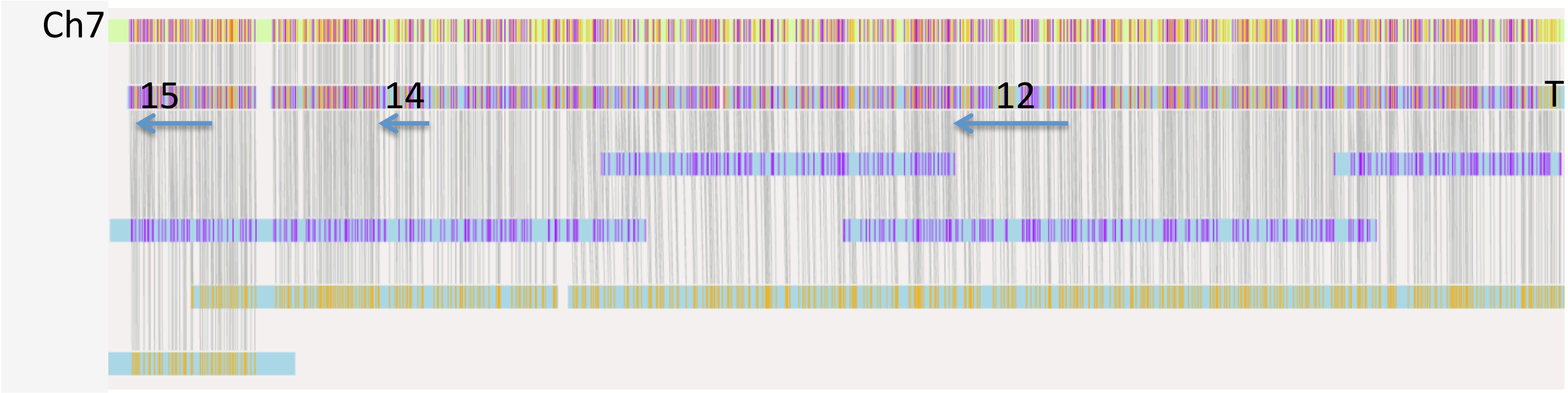

Figure shows the Final Optical Map of M4 Chr7 (H1) (2.8Mb) (top) and the alignment of 1) *in silico* digested M4 contigs 15, 14 & 12 (middle orange bars), 2) M4 enzyme Nt.BbvC1 optical maps (purple) and 3) M4 enzyme Nt.BspQ1 optical maps (bottom yellow). Telomeres are also shown (T).

S1 Fig. M4 Optical Map for Chromosome 7 (10 of 14 slides)

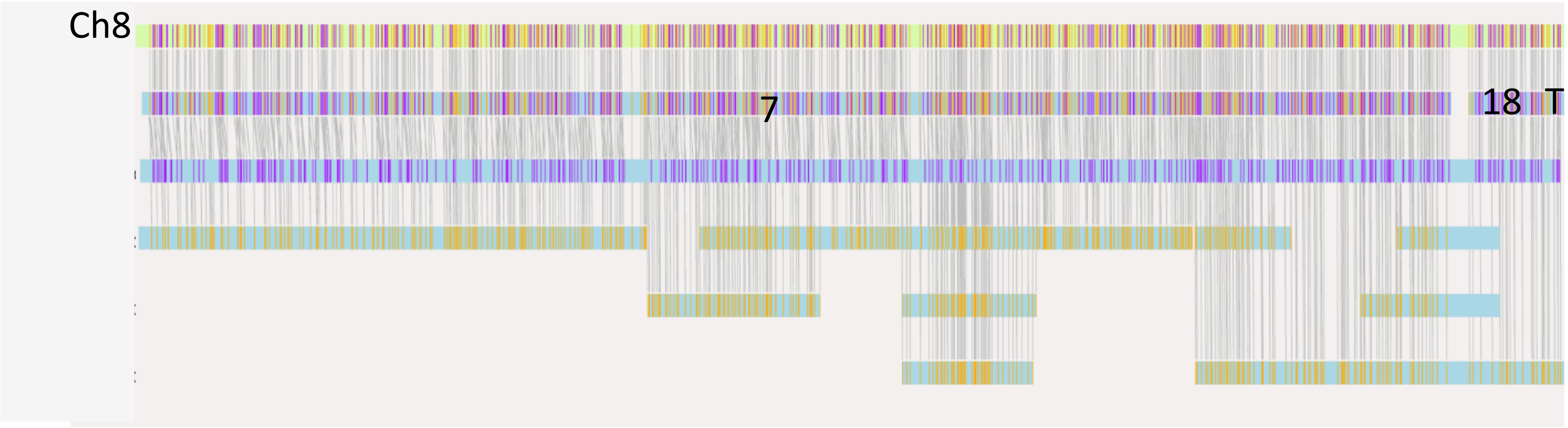

Figure shows the Final Optical Map of M4 Chr8 (H5) (2.4Mb) (top) and the alignment of 1) *in silico* digested M4 contigs 7 & 18 (middle orange bars), 2) M4 enzyme Nt.BbvC1 optical maps (purple) and 3) M4 enzyme Nt.BspQ1 optical maps (bottom yellow). Telomeres are also shown (T).

S1 Fig. M4 Optical Map for Chromosome 8 (11 of 14 slides)

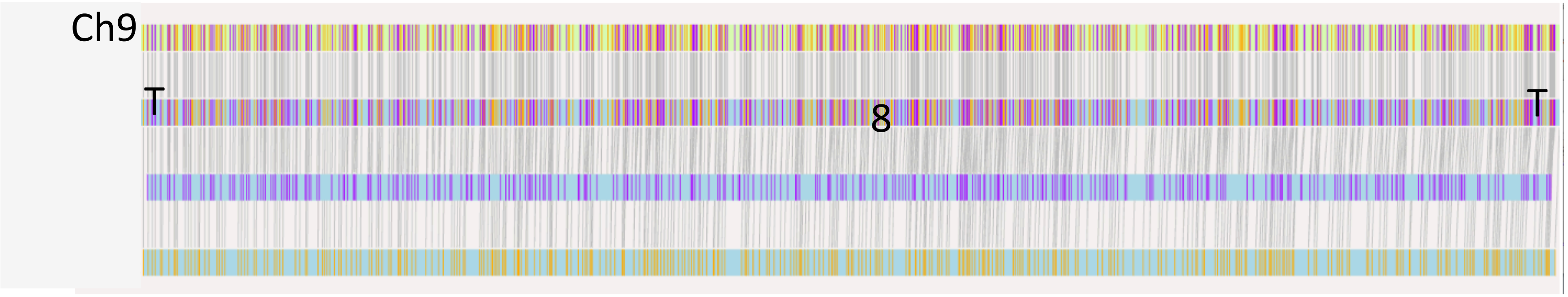

Figure shows the Final Optical Map of M4 Chr9 (H10) (1.5Mb) (top) and the alignment of 1) *in silico* digested M4 contig 8 (middle orange bars), 2) M4 enzyme Nt.BbvC1 optical map (purple) and 3) M4 enzyme Nt.BspQ1 optical map (bottom yellow). Telomeres are also shown (T).

S1 Fig. M4 Optical Map for Chromosome 9 (12 of 14 slides)

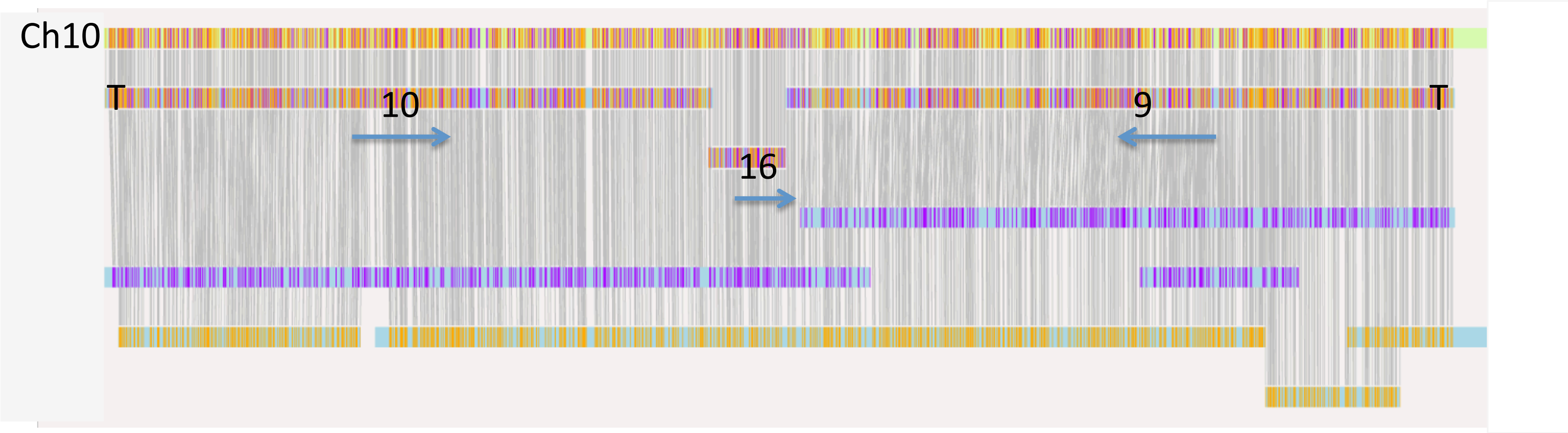

Figure shows the Final Optical Map of M4 Chr10 (H10) (4.3Mb) (top) and the alignment of 1) *in silico* digested M4 contig 10, 16 & 9 (middle orange bars), 2) M4 enzyme Nt.BbvC1 optical map (purple) and 3) M4 enzyme Nt.BspQ1 optical map (bottom yellow). Telomeres are also shown (T).

S1 Fig. M4 Optical Map for Chromosome 10 (13 of 14 slides)

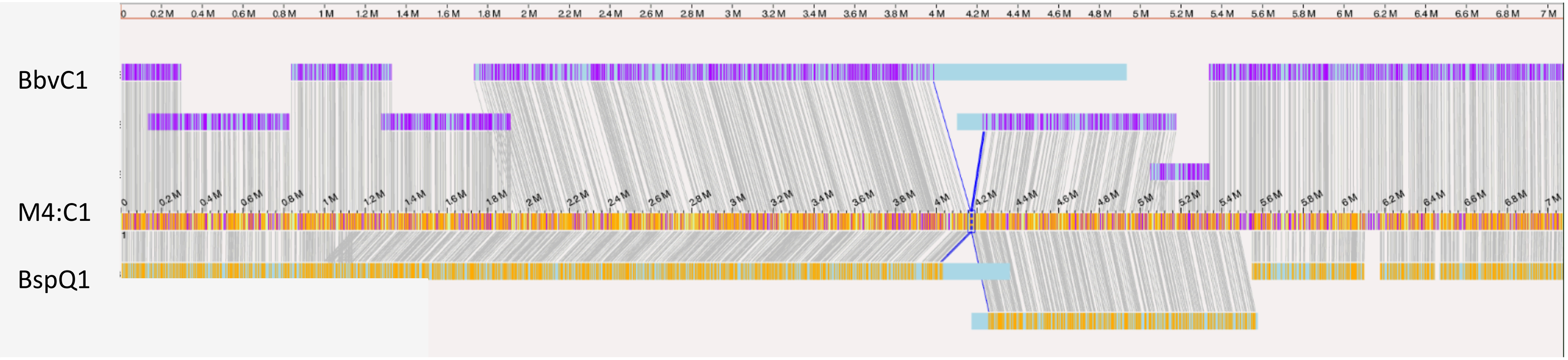

Figure shows the alignment of the M4 enzyme Nt.BbvC1 optical map (purple) and M4 enzyme Nt.BspQ1 optical map (bottom yellow) to the *in silico* digested M4 contig 1 (7Mb) (middle orange bar), and lack of support the contig at the ~4Mb region.

S1 Fig. M4 contig1 two enzyme optical map alignments
