# Supplementary material for: Comparative genomics of the wheat fungal pathogen Pyrenophora tritici-repentis reveals chromosomal variations and genome plasticity
Source: BMC Genomics. 2018 Apr 23;19:279. doi: 10.1186/s12864-018-4680-3 (PMC5913888; doi:10.1186/s12864-018-4680-3)
Supplement: Supplementary file 2 — PCR validation of M4 PacBio pre-optical map assembly. A) Table of PCR results to validate M4 PacBio genome regions. B) Three PCR gel results show primer results for Ptr isolates M4 (M), DW5 (D) and negative no template control (C). The amplified product bands are shown for M4 contig 1, 3, 6, 9 and 17. C) Pre-optical mapM4 contig alignments to BFP chromosomes are shown at ≥90% identity and ≥ 5 Kbps in length. M4 contigs are displayed above alignments and BFP chromosomes below. Red connecting lines represent sequence alignments in the same orientation between M4 and BFP sequences, and reverse-complemented alignments are blue. Grey markers indicate distal ends of contigs with identifiable telomere motifs. Regions validated by PCR in M4 are indicated in green on contig 1, contig 3, contig 6 and contig 9. (PDF 631 kb) [file 12864_2018_4680_MOESM2_ESM.pdf]

A

Contig	Gel lane	Left primer position (bp)	Right primer position (bp)	Product	Left GC%	Right GC%	M4	DW5	Pt-C-BFP *in silico*
**Contig 3**	A	2102043	2102988	945	50	50	+	-	-
	B	2101652	2102487	835	54.55	45.45	+	-	-
**Contig 6**	A	488429	489442	1013	50	50	+	-	-
	B	488378	489442	1064	50	50	+	-	-
**Contig 9**	A	548609	549759	1150	50	50	+	+	-
	B	548613	549759	1146	50	50	+	+	-
**Contig 1**	A	4109952	4113126	3174	50	50	+	+	-
	B	4111821	4115104	3283	45.45	54.55	+	-	-
	C	4118742	4121830	3088	50	50	+	-	-
	D	4113998	4118229	4231	50	50	+	-	-
	E	4105316	4107227	1912	50	50	+	-	-
	F	4105316	4107095	1780	50	50	+	-	-
	G	4315899	4317853	1955	50	45.45	+	+	-
	H	4316084	4318048	1965	45.45	50	+	+	-
	I	5798243	5800132	1890	50	45.45	+	-	-
	J	5798150	5800132	1983	50	45.45	+	-	-
**Contig 17**	A	48325	51692	3367	43.48	40.91	+	+	-
	B	93516	95494	1978	40.91	50	+	+	-

B

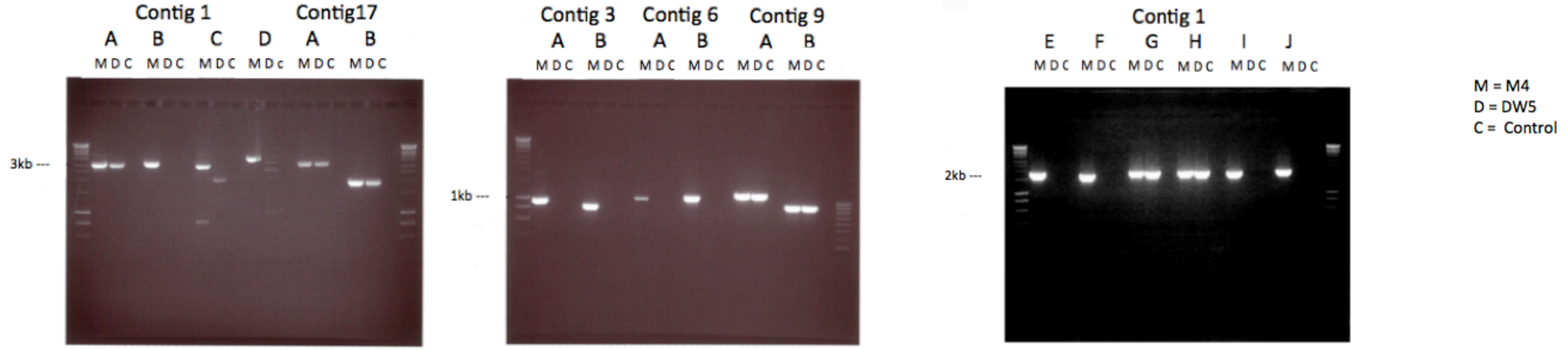

S2A & B Fig. PCR validation of M4 PacBio assembly (1 of 2 slides)

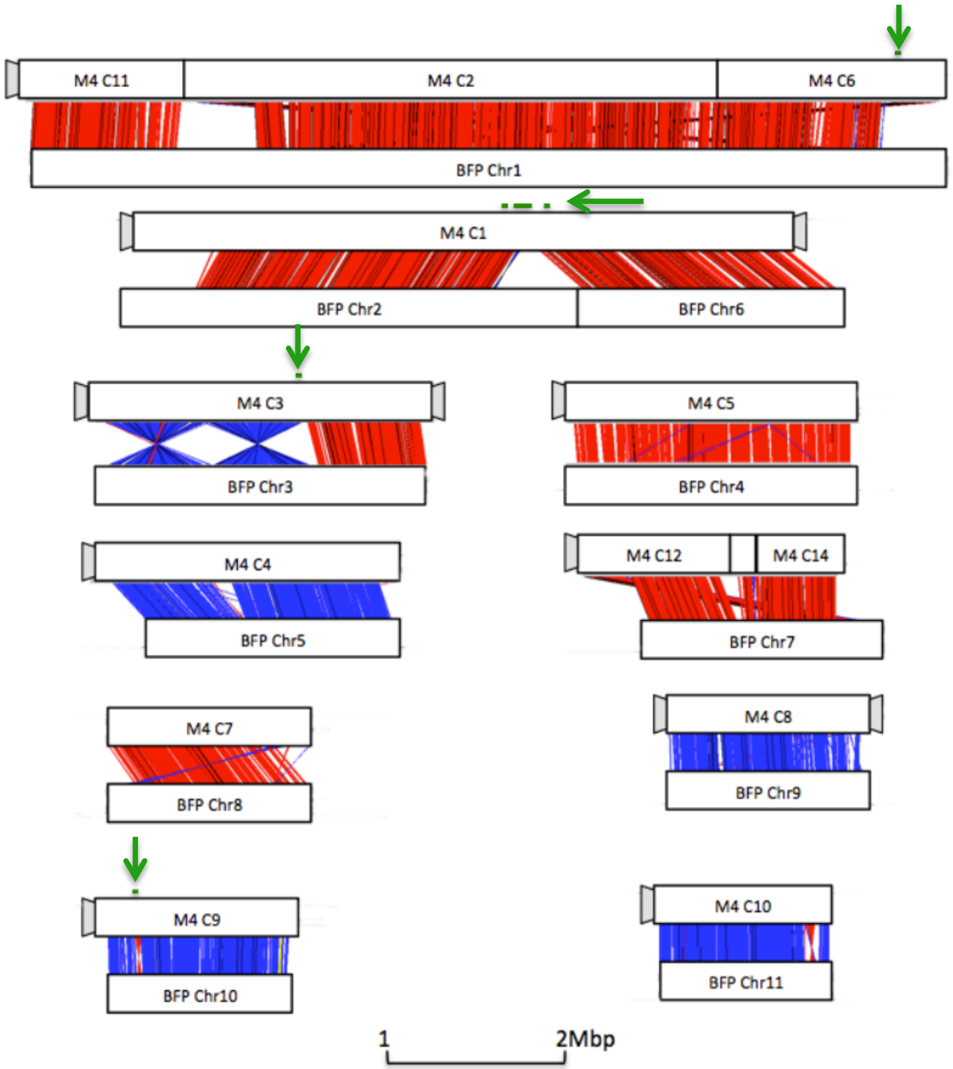

C

S2C Fig. PCR validation of pre optical map M4 PacBio contig sequence (2 of 2 slides)

Sequence alignment is shown between M4 PacBio assembly and BFP. The regions confirmed by PCR are indicated (green arrow) above M4 contig 1, 3, 6, 9 and 17.
