# Supplementary figures and images for: Comparative genomics of the wheat fungal pathogen Pyrenophora tritici-repentis reveals chromosomal variations and genome plasticity
Source: BMC Genomics. 2018 Apr 23;19:279. doi: 10.1186/s12864-018-4680-3 (PMC5913888; doi:10.1186/s12864-018-4680-3)

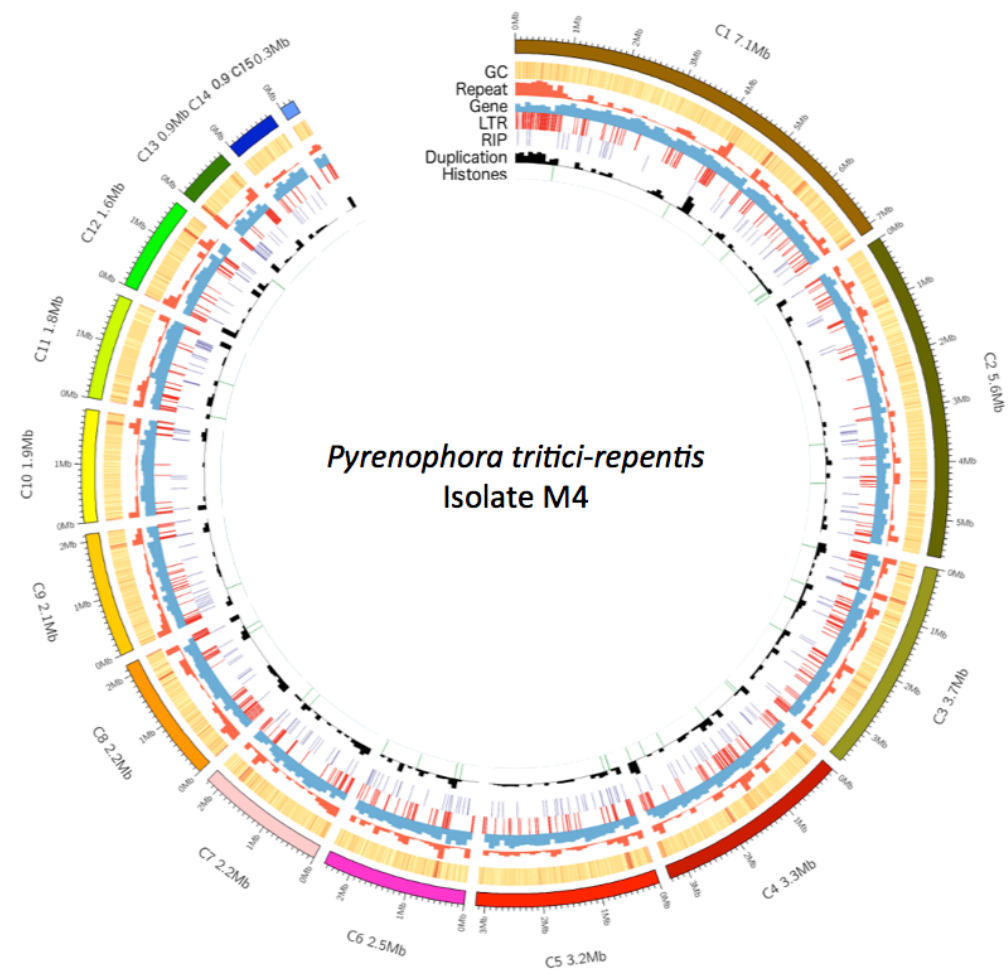

**S3 Fig. Repeat content plot for M4 genome**

Supplement: Supplementary file 3 — Repeat content plot for M4 genome. Circos plot displays repeat and gene content for M4 genome (contigs 1–15). Displayed in order is a heat map of GC content (red is high AT content), gene frequency over a 100Kbp window, repeat frequency (100Kbp window), and positions of LTR, segmental duplications and histones genes. Major repeat regions are found in contig distal locations and associated with high LTR content. (PDF 322 kb) [file 12864_2018_4680_MOESM3_ESM.pdf]

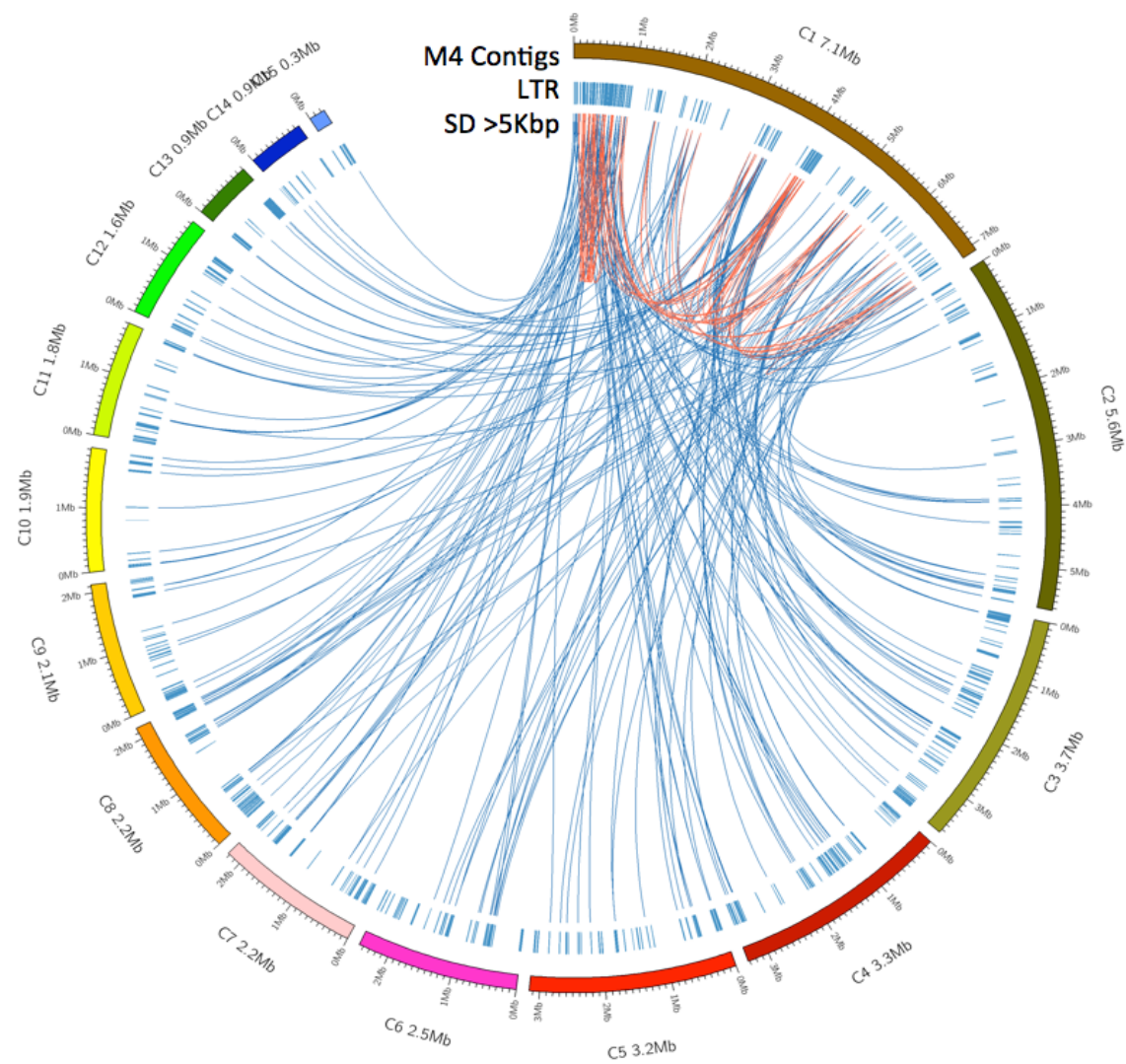

**S4 Fig. M4 plot of large segmental duplications.**

Supplement: Supplementary file 6 — M4 plot of large segmental duplications. Circos plot displays M4 genome LTR positions and segmental duplications (SD) greater than 5 kb and 90% nucleotide identity between contig 1 and the rest of the genome (contigs 1–15), inter-contig (blue links) and intra-contig (red links). Intra-contig links are shown mainly between the telomeres and centromere of contig 1. (PDF 394 kb) [file 12864_2018_4680_MOESM6_ESM.pdf]

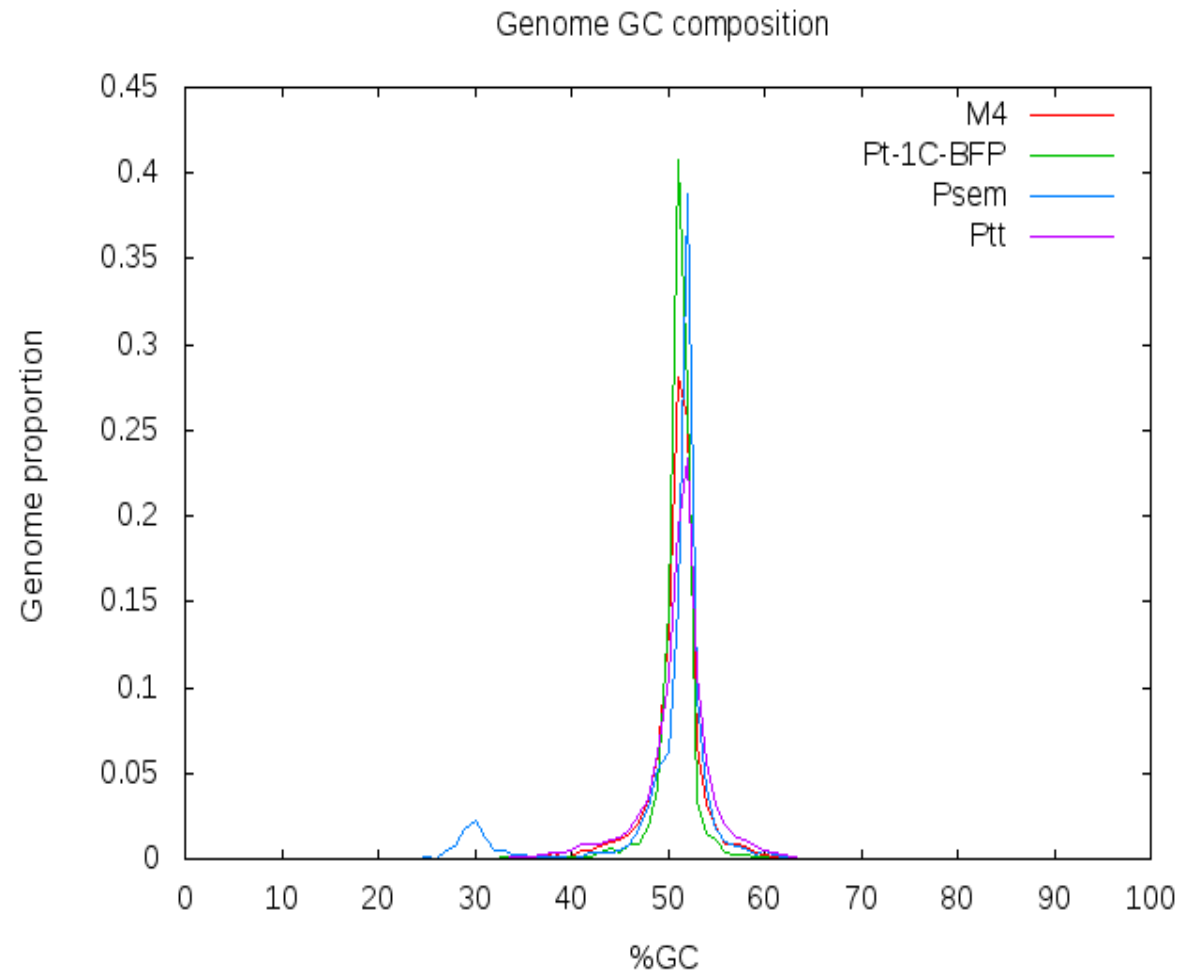

**S8 Fig. *Pyrenophora* genome AT/GC composition plots**

Supplement: Supplementary file 8 — Pyrenophora genome AT/GC composition plots. Pyrenophora genome AT/GC composition plots, minus the mitochondrial genome. Plotted genomes are Pyrenophora tritici-repentis M4 and Pt-1CBFP, Pyrenophora semeniperda (Psem) and Pyrenophora teres f. teres (Ptt). Only Psem displays a bimodal plot of GC composition (blue). (PDF 40 kb) [file 12864_2018_4680_MOESM8_ESM.pdf]

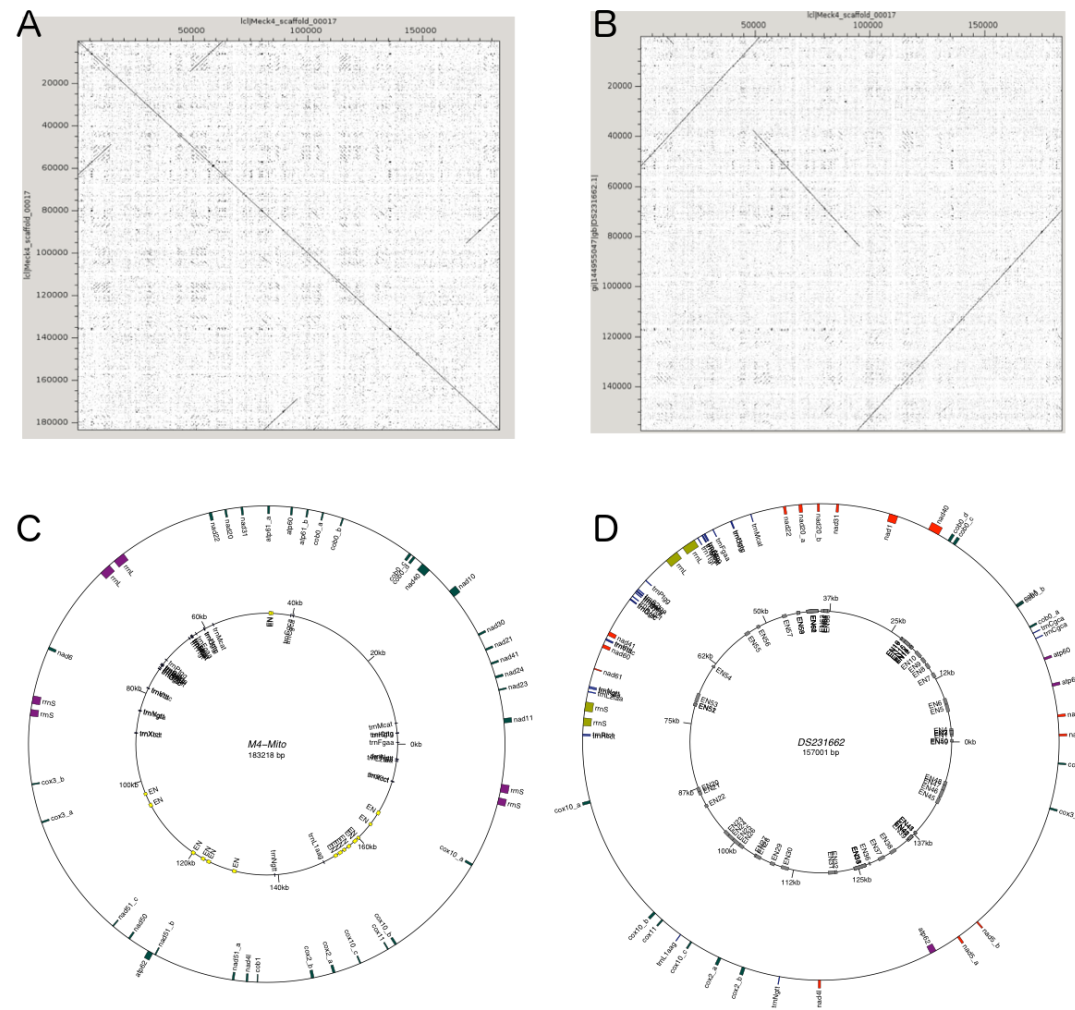

**S9 Fig. M4 and BFP Mitochondrial analysis**

Supplement: Supplementary file 9 — M4 and BFP Mitochondrial analysis. A) M4 Mitochondrial contig 17 self-plot shows two events of inverted duplication. The first 13 kb of the mitochondrial contig has an inverted duplication at 50–63 kb and the last 13 kb has an inverted duplication at 80–93 kb (resulting in an extra two copies of small ribosomal RNAs). This is not a typical pattern for confirming circularisation. B) Dotplot of M4 versus BFP mitochondrial contigs. C) M4 Mitochondrial genome (183Kb) and D) BFP (157Kb) are shown left and right respectively. Mapped to the outer ring are protein-coding genes and ribosomal RNA, the inner ring shows the positions of the endonucleases and transfer RNAs. (PDF 608 kb) [file 12864_2018_4680_MOESM9_ESM.pdf]

### *Pyrenophora tritici-repentis* isolate codon usage

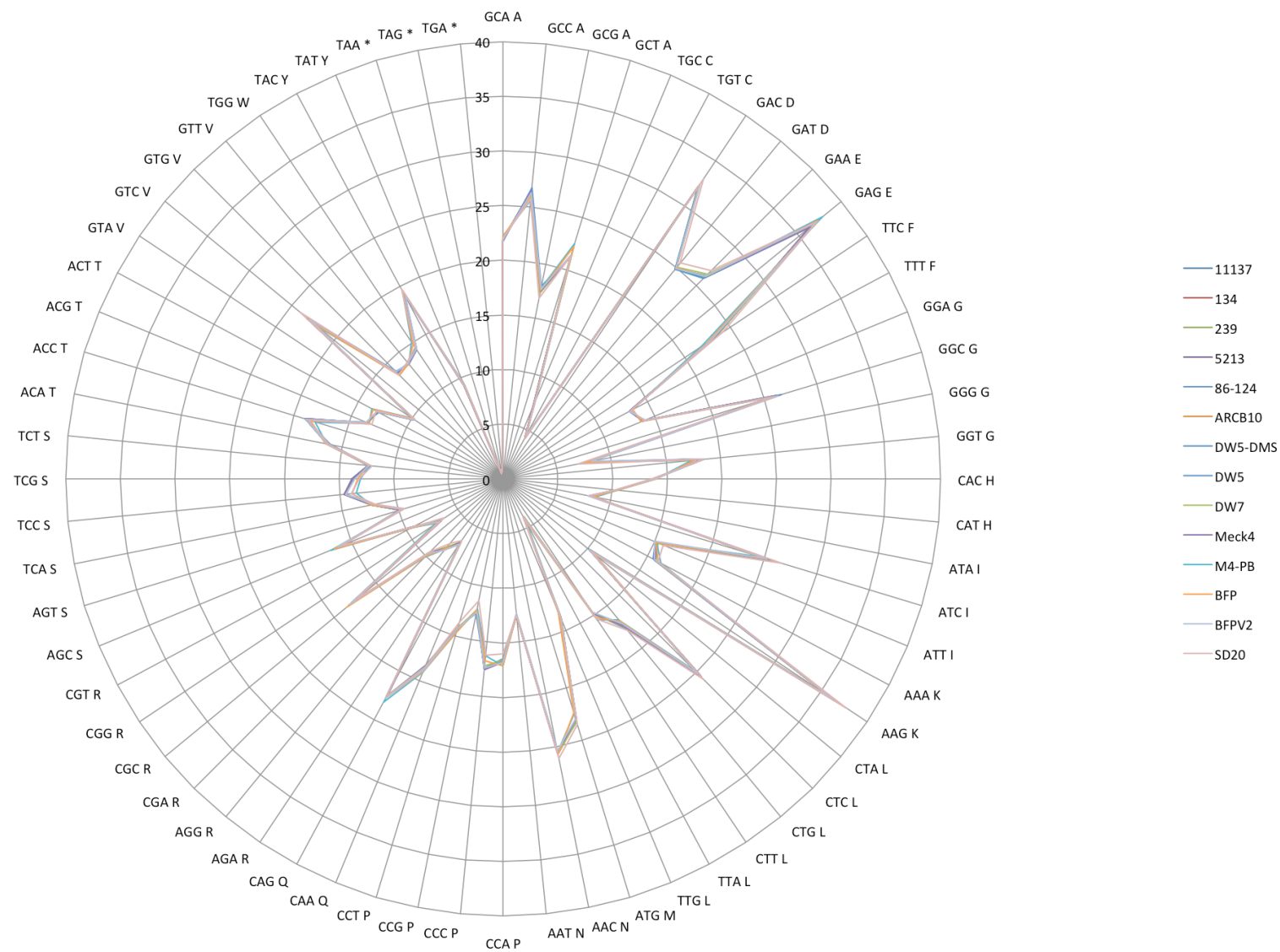

**S13 Fig. Ptr isolates codon usage radar plot**

Supplement: Supplementary file 13 — Ptr isolates codon usage radar plot. (PDF 601 kb) [file 12864_2018_4680_MOESM13_ESM.pdf]

Reciprocal sequence identity  $\geq 90$  and coverage  $\geq 90\%$

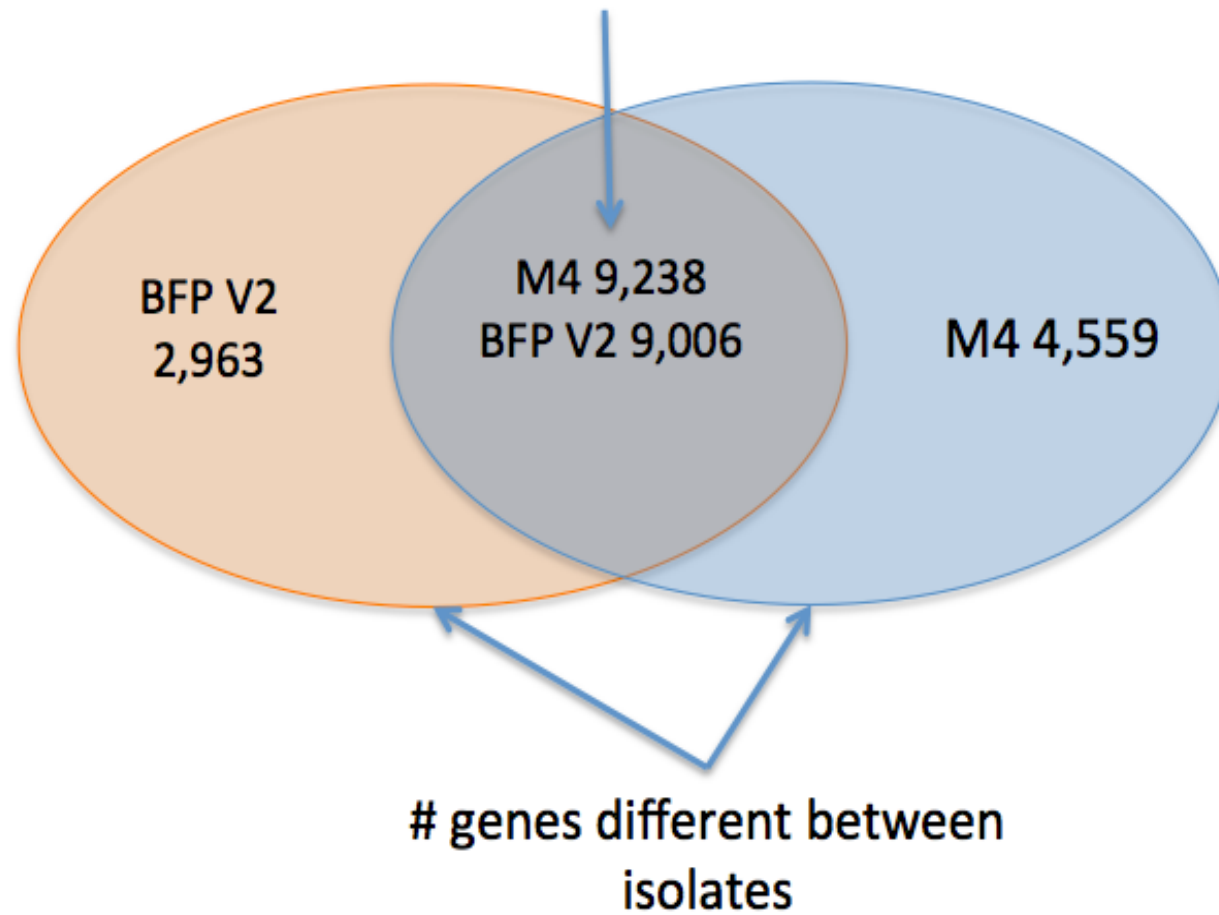

**S14 Fig. M4 and BFP highly conserved genes.**

Supplement: Supplementary file 14 — M4 and BFP highly conserved genes. M4 and BFP gene overlap at 90% sequence coverage and identity. M4 has a larger core gene number due to increase copy number of orthologous genes compared to BFP. (PDF 87 kb) [file 12864_2018_4680_MOESM14_ESM.pdf]

| Age Group | Number of People |
|-----------|------------------|
| 0-10      | 100              |
| 11-20     | 85               |
| 21-30     | 75               |
| 31-40     | 65               |
| 41-50     | 55               |
| 51-60     | 45               |
| 61-70     | 35               |
| 71-80     | 25               |
| 81-90     | 15               |
| 91-100    | 10               |

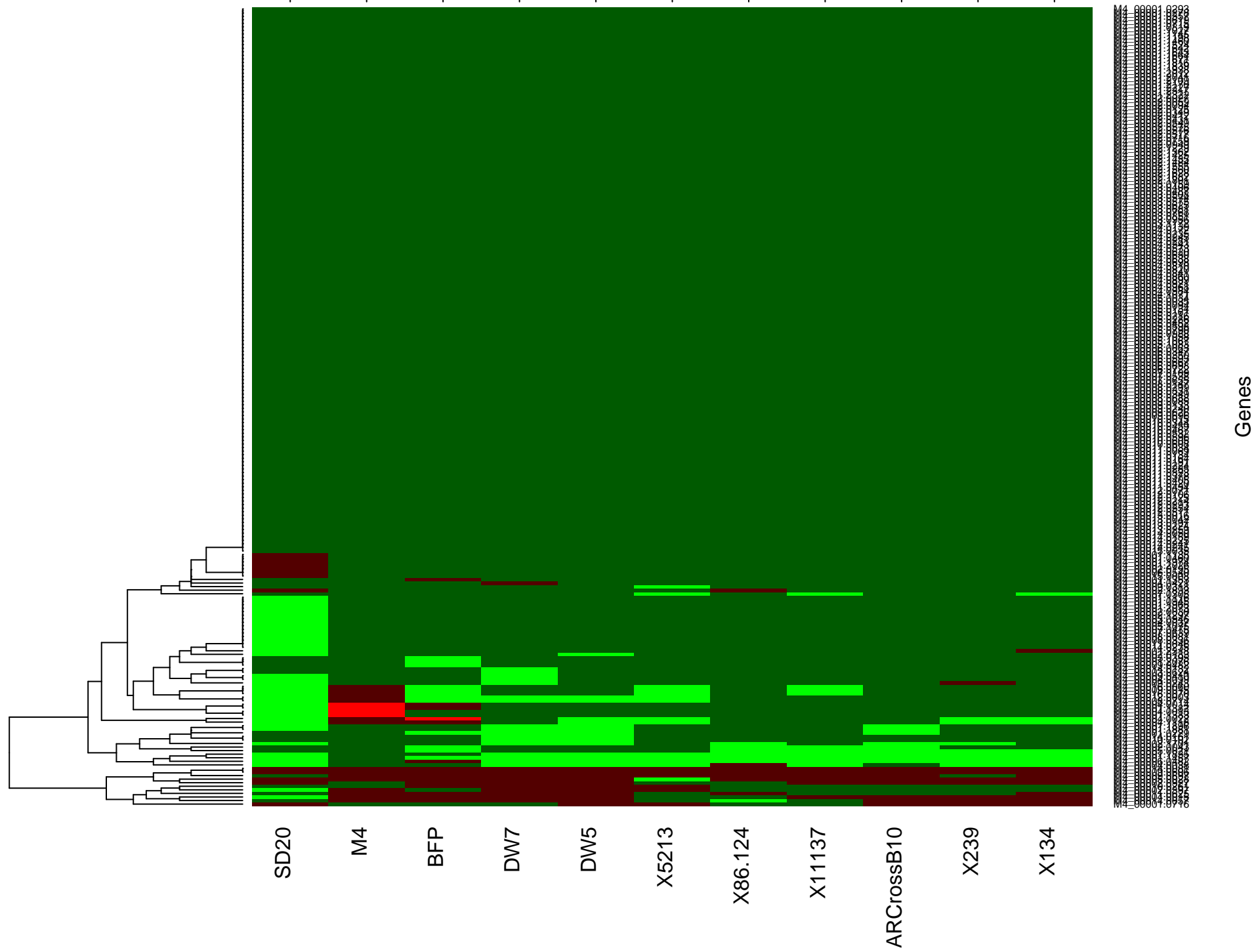

Supplement: Supplementary file 17 — Predicted effector homolog gene counts in Ptr. Heatmap shows M4 predicted effectors (Effectorp probability score >= 0.5) homologue counts. Gene sequences were searched at 90% identity and coverage (BLATX) against all Ptr isolates genomes. (PDF 41 kb) [file 12864_2018_4680_MOESM17_ESM.pdf]

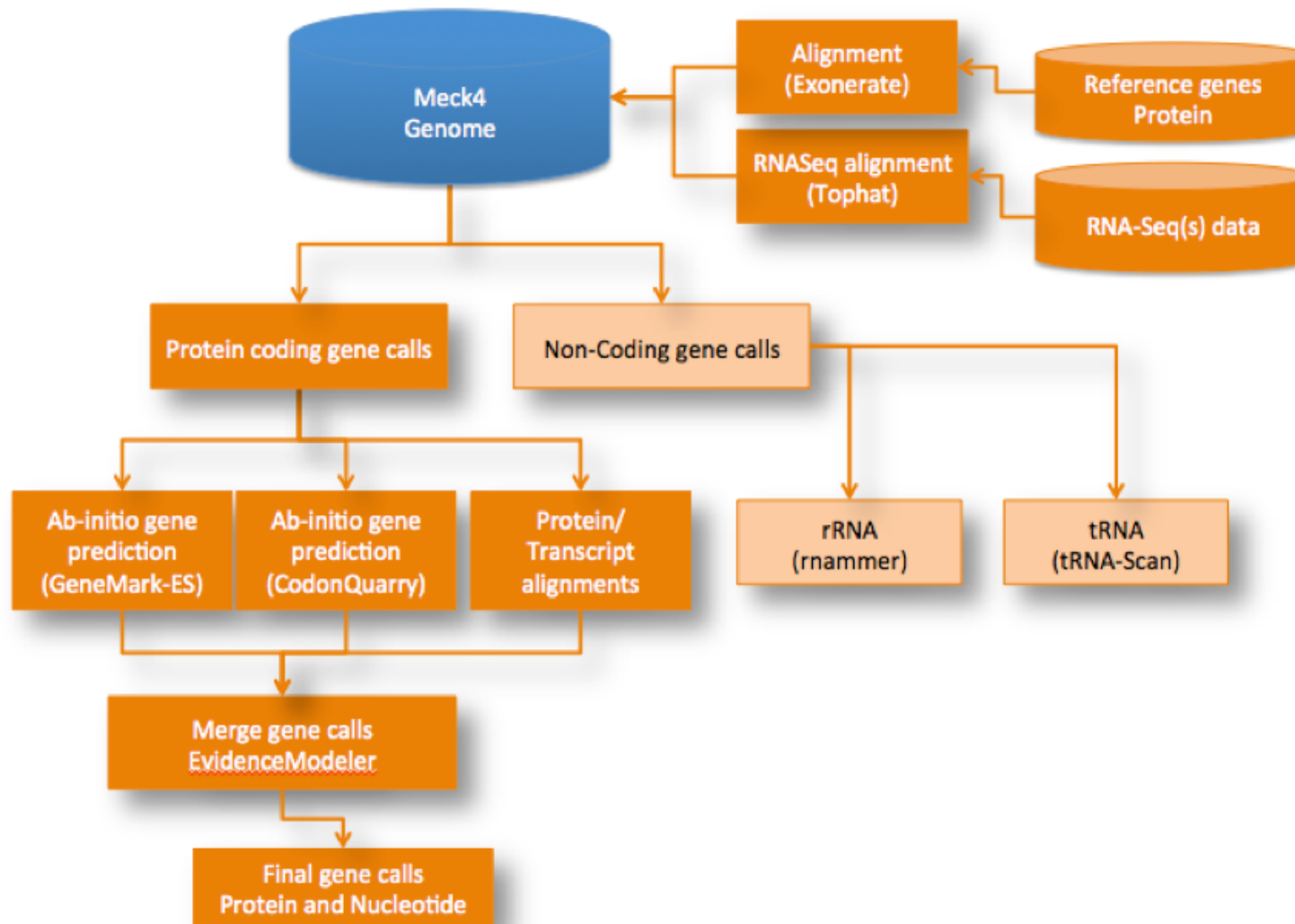

**S24 Fig. Workflow and tools utilised for all isolate genome annotation**

Supplement: Supplementary file 24 — Workflow and tools utilised for all isolate genome annotation. Workflow and tools utilised for all isolate genome annotation. Genome annotation flow chart overview shows input genome data (disc shape) and tasks implemented (rectangles) for protein and RNA-Seq alignments, three ab initio gene predictions for protein coding genes and predictions for non-coding rRNA and tRNA. (PDF 238 kb) [file 12864_2018_4680_MOESM24_ESM.pdf]
