# Supplementary material for: Comparative genomics of the wheat fungal pathogen Pyrenophora tritici-repentis reveals chromosomal variations and genome plasticity
Source: BMC Genomics. 2018 Apr 23;19:279. doi: 10.1186/s12864-018-4680-3 (PMC5913888; doi:10.1186/s12864-018-4680-3)
Supplement: Supplementary file 10 — Mitochondrial cob gene analysis. A) M4 mitochondrial cob gene spans 12 kb (26–39 kb) with intron spans greater than 2 kb. B) Mitochondrial cytochrome b protein multiple sequence alignment for Zymoseptoria tritici (AAP81933), Ptt (DQ919067.1), Ptr (GenBank DQ919068), SN79_contig_2277, SN4_contig_1698, SN15 (NC_009746), 11,137_00499, 134 _00534, 5213_00542, 86-124_00253, AR CrossB10_00129, DW5_00657, M4_00017, Ptt (NW_00352501 and 00356055), and BFP (DS231662.1) shows three known mutation sites for fungicide resistance. (PDF 102 kb) [file 12864_2018_4680_MOESM10_ESM.pdf]

A. M4 Mitochondrial cob gene (26-39 kbp)

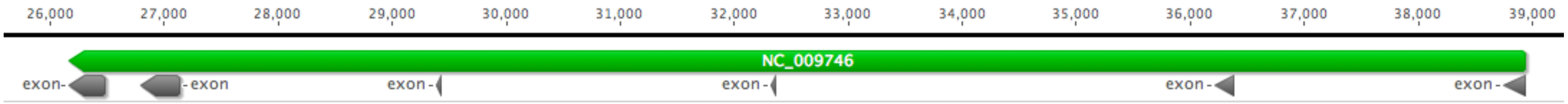

B. Mitochondrial cob protein alignments

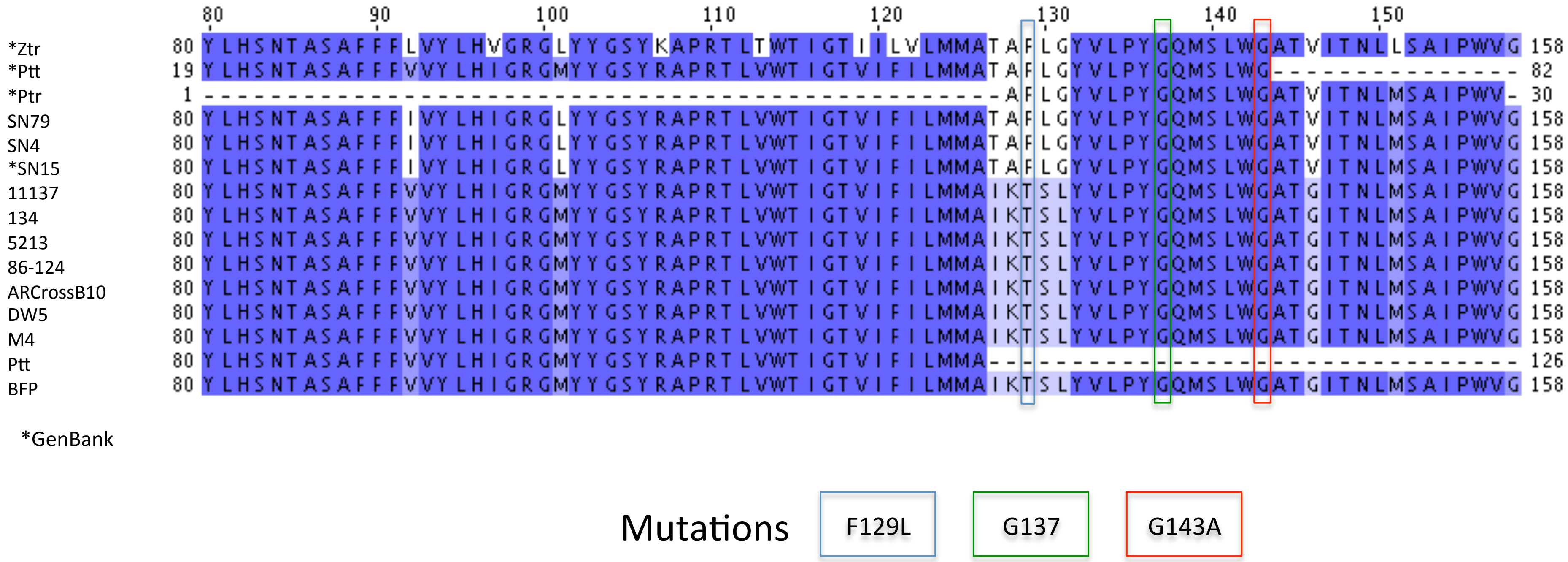

S10 Fig. Mitochondrial cob protein analysis
