# Supplementary material for: Comparative genomics of the wheat fungal pathogen Pyrenophora tritici-repentis reveals chromosomal variations and genome plasticity
Source: BMC Genomics. 2018 Apr 23;19:279. doi: 10.1186/s12864-018-4680-3 (PMC5913888; doi:10.1186/s12864-018-4680-3)
Supplement: Supplementary file 11 — Genome sequence plots of M4 compared to other isolate fungi. Page 1. Protein sequence plots of M4 (vertical axis) against necrotrophic fungi Parastagonospora nodorum (Sn15), L. maculans, P. teres f. teres, P. seminiperda, B.maydis isolates ATCC48331 and C5 (Teleomorph Cochliobolus heterostrophus) B. zeicola and B. sororkiniania (Bsoro) scaffolds (horizontal axes) show good alignment protein conservation to M4. Page 2. Genome sequence plots of M4 compared to other Ptr isolate contigs. Page 3. Whole genome phylogeny of Ptr isolates including M4 Illumina assembly. (PDF 1243 kb) [file 12864_2018_4680_MOESM11_ESM.pdf]

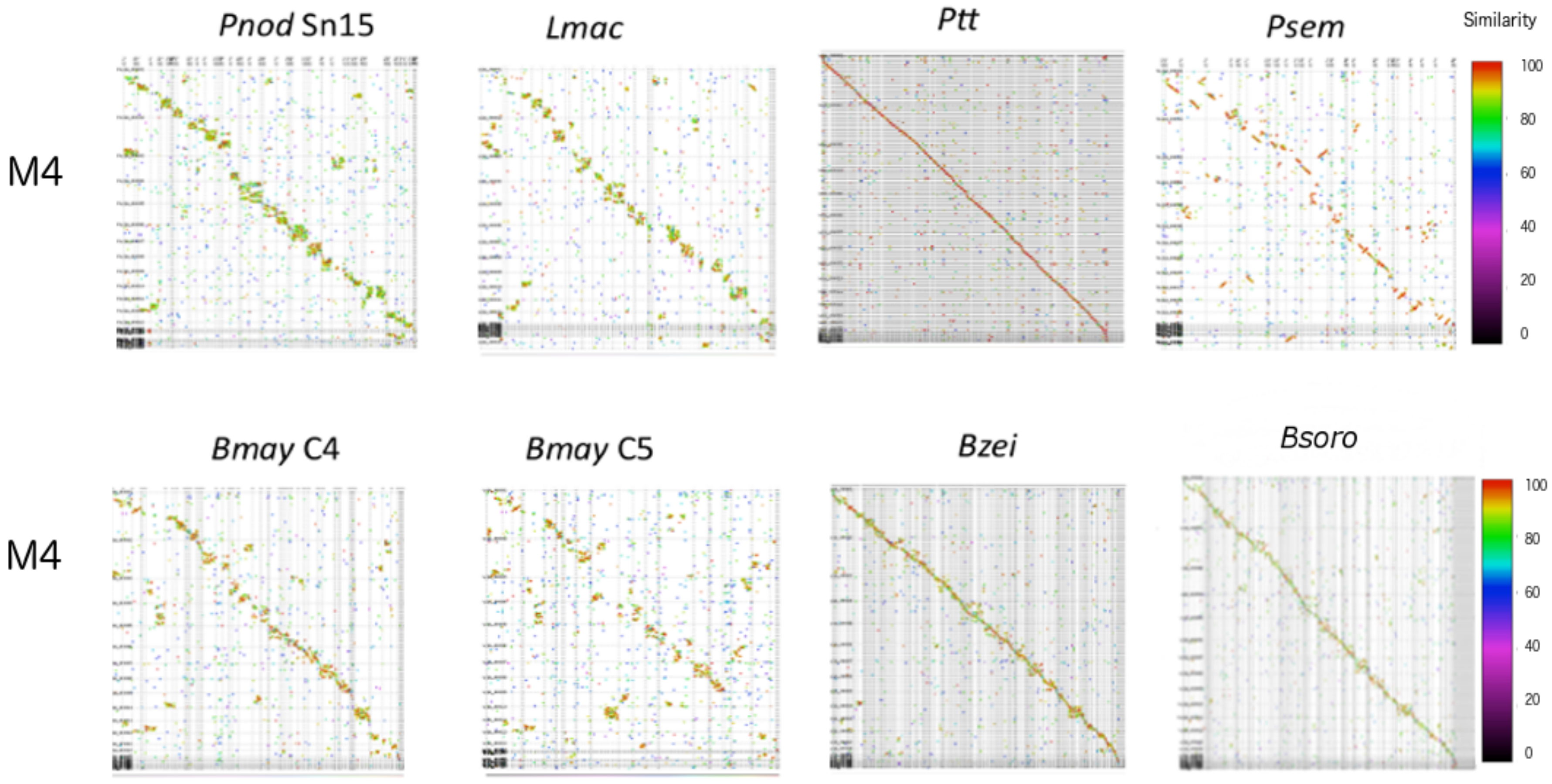

S11 Fig. Genome sequence plots of M4 compared to other necrotrophic fungi (page 1 of 3)

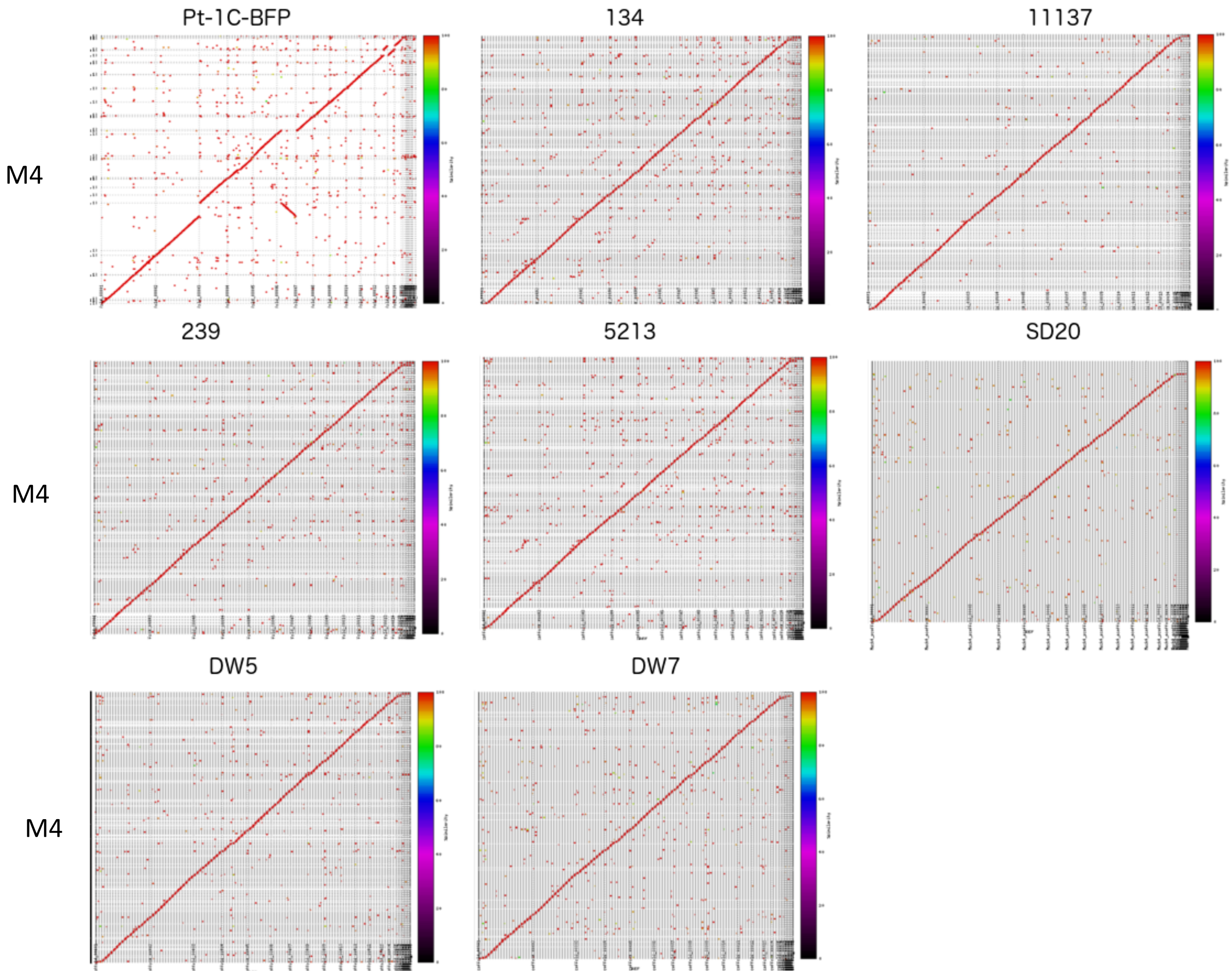

S Figure. Sequence plots show M4 assembly (all horizontal axes) genome nucleotide alignments to race 1 isolates BFP, 134, 1137, 239, 5213, race 4 isolate SD20, and race 5 isolates DW5 and DW7 on the vertical axis. All isolates have good coverage of the M4 genome for comparative analysis. Sequence identity legends are displays to the right of each plot and indicate all isolate alignments are appear better than 95% similarity (red).

S11 Fig. Genome sequence plots of M4 compared to other Ptr isolates (page 2 of 3)

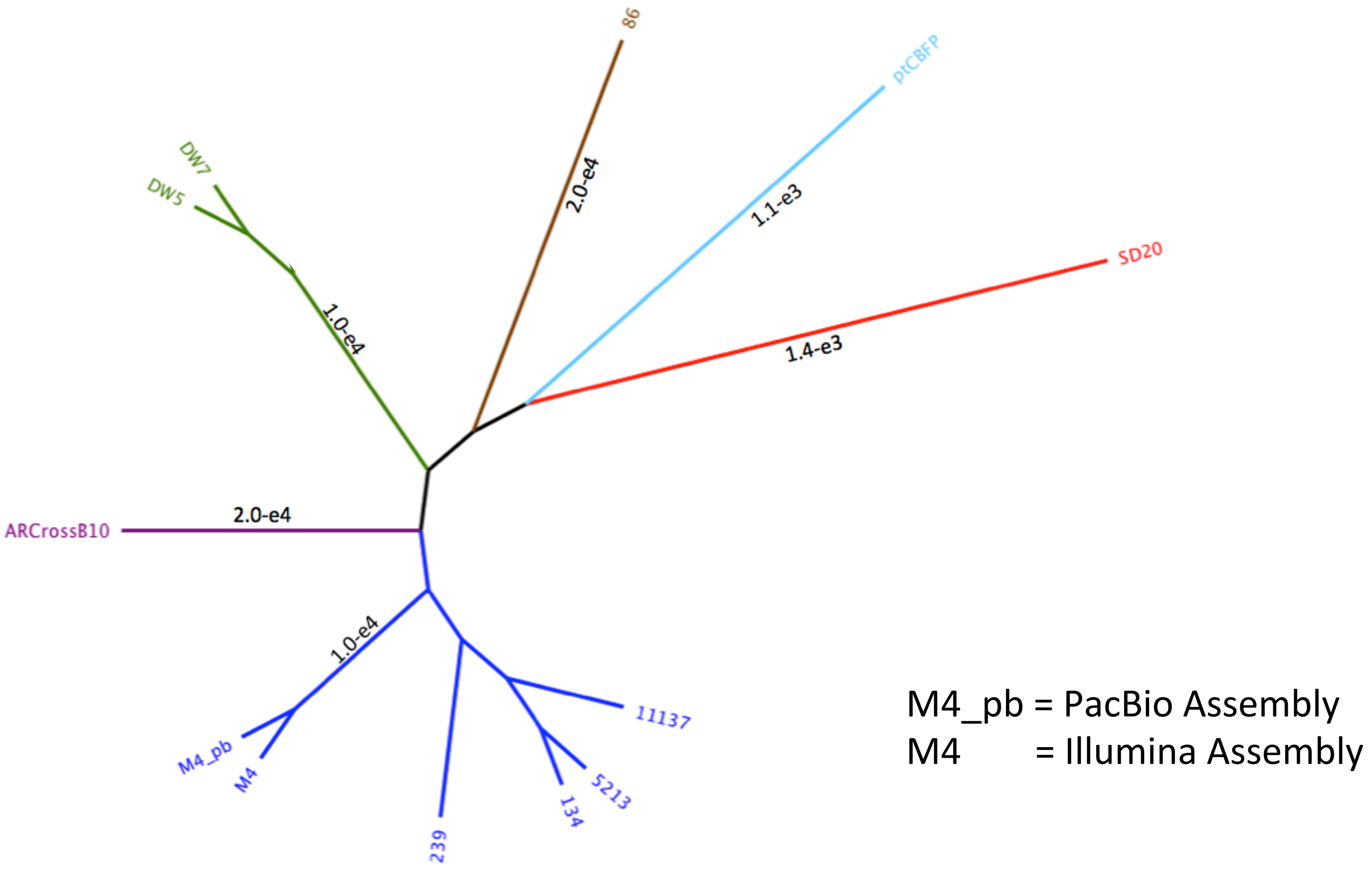

Ptr whole genome phylogenetic tree shows raw branch lengths on a transformed radial cladogram for SD20 (race 4 red), 86-124 (race 2 brown), DW5 and DW7 (race 5 green), AR CrossB10 (new race purple), and M4 PacBio (M4_pb) and Illumina (M4), 134, 239, 5213, 11137 and BFP (race 1 dark blue).

S11 Fig. Whole genome phylogeny of Ptr isolates (page 3 of 3)
