# Supplementary material for: Comparative genomics of the wheat fungal pathogen Pyrenophora tritici-repentis reveals chromosomal variations and genome plasticity
Source: BMC Genomics. 2018 Apr 23;19:279. doi: 10.1186/s12864-018-4680-3 (PMC5913888; doi:10.1186/s12864-018-4680-3)
Supplement: Supplementary file 19 — M4 and BFP alignment of the ToxA region. A) Sequence plot for M4 contig1 ToxA 170Kb region (5.65-58 Mb) on the horizontal axis and BFP DS231618 170Kb region (1.36–1.55 Mb) on the vertical axis show a number of sequence variations and features in common. B) Sequence plot shows three major deletion sites in M4 and the AT-rich region upstream of the ToxA gene. C) Alignment between M4 and Pt-1C-BFP isolates show an alternate view of the three large insertions/deletions in the ToxA race 1 specific region (blue arrowed region above and below Fig) and distal flanking homologous sections (blue boxes connected by blue dotted line). The deletion positions in M4 contig1 are 5,685,572 bp, 5,724,456 bp, 5,799,713 bp which correspond to Pt-1C-BFP DS231618 4, 6.9 and 5.4 kb insertions respectively. The total length of M4 ToxA race 1 region is ~129Kb and Pt-1C-BFP is ~145bpkb (regions in common are shaded in pink). M4 genes are plotted between the two sequence similarity plots (light pink). (PDF 647 kb) [file 12864_2018_4680_MOESM19_ESM.pdf]

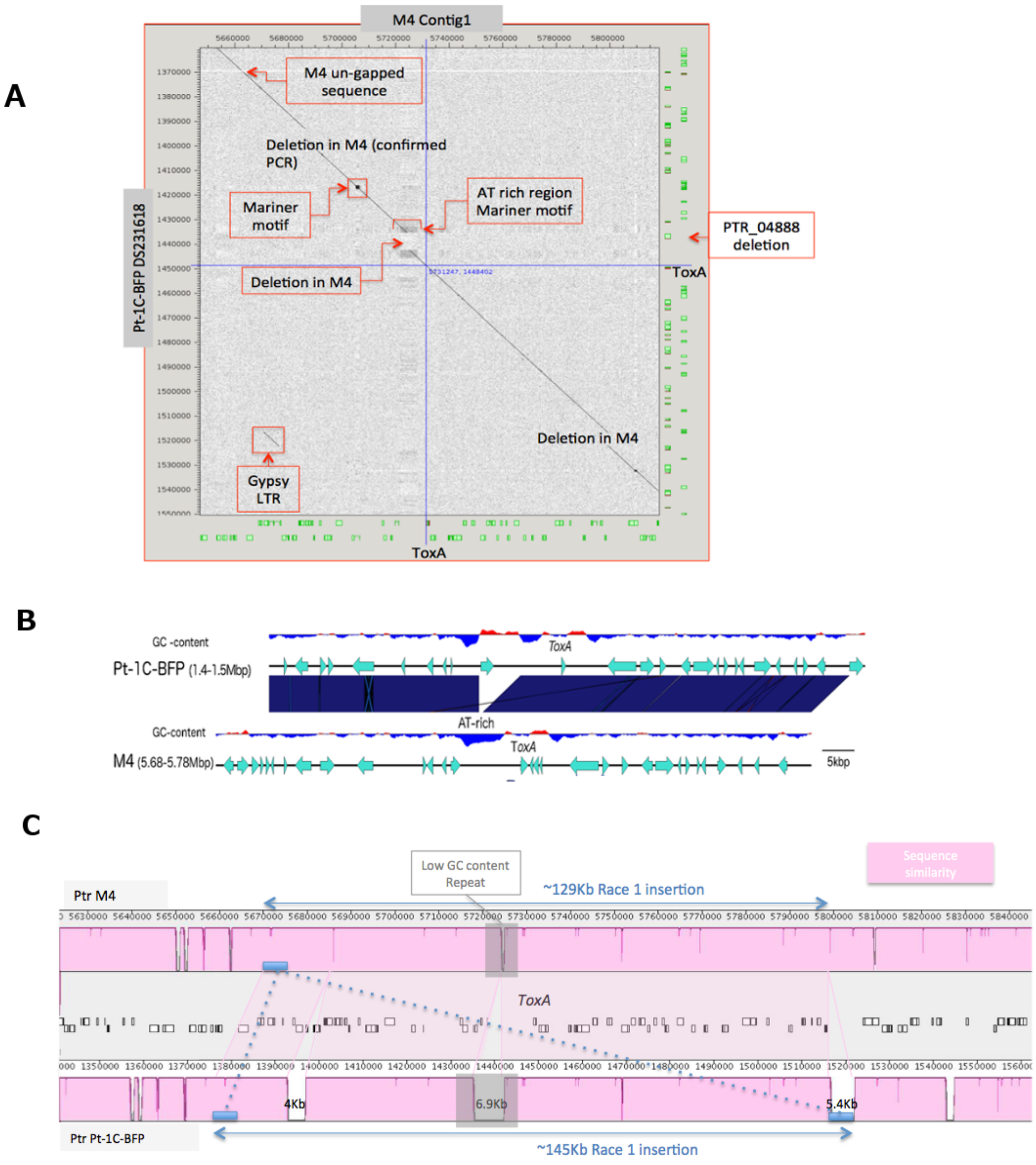

The largest M4 deleted region (6.9 kb) has occurred in AT-rich region upstream from the *ToxA* gene, this region in BFP carried a hypothetical gene *PTRG_04888*. The deleted gene had significant matches to two protein domain families, glycosyl transferase family group 2 (pfam13632) and cellulose synthase catalytic CESA family (cd06423). This gene was searched in the remainder of the M4 genome and a M4 homolog (*1.1417*) was found upstream in M4 (contig1B: 160,434-163,312bp) with 99.88% amino acid identity to *PTRG_04888*.

S19 Fig. M4 and BFP alignment of the ToxA region
