# Supplementary material for: Comparative genomics of the wheat fungal pathogen Pyrenophora tritici-repentis reveals chromosomal variations and genome plasticity
Source: BMC Genomics. 2018 Apr 23;19:279. doi: 10.1186/s12864-018-4680-3 (PMC5913888; doi:10.1186/s12864-018-4680-3)
Supplement: Supplementary file 20 — Whole-genome overview of the gene and repeat features of the Australian M4 reference isolate, and comparisons to alternate Ptr isolate genomes. Whole-genome overview of the gene and repeat features of the Australian M4 reference isolate, and comparisons to alternate Ptr isolate genomes. A) The six outer labelled rings illustrate: M4 genome contigs 1–15; a heat map of M4 local GC content within 10 kb windows (low (AT-rich) = red); M4 repeat density within 100 kb windows (red); M4 gene density within 100 kb windows (blue); LTR content (red); and genome RIP indexed regions (purple). The eight inner rings show coverage of M4 genome contigs 1–15 by alignments of alternate Ptr isolates in windows of 100 kb. Australian race 1 isolates 134, 239, 111,137, and 5213 and North American race 1 BFP are indicated in blue. Race 2 86-124 and the new race AR CrossB10 are shown in brown and purple respectively. Race 5 DW5, DW7 are red and orange. Race 4 SD20 is green. The ToxA position is marked in contig 1. B) A higher resolution view of the ToxA region on M4 contig 1, labelled as per part A, showing presence of this region in race 1 isolates and absence of this region in race 4 and 5 isolates. (PDF 770 kb) [file 12864_2018_4680_MOESM20_ESM.pdf]

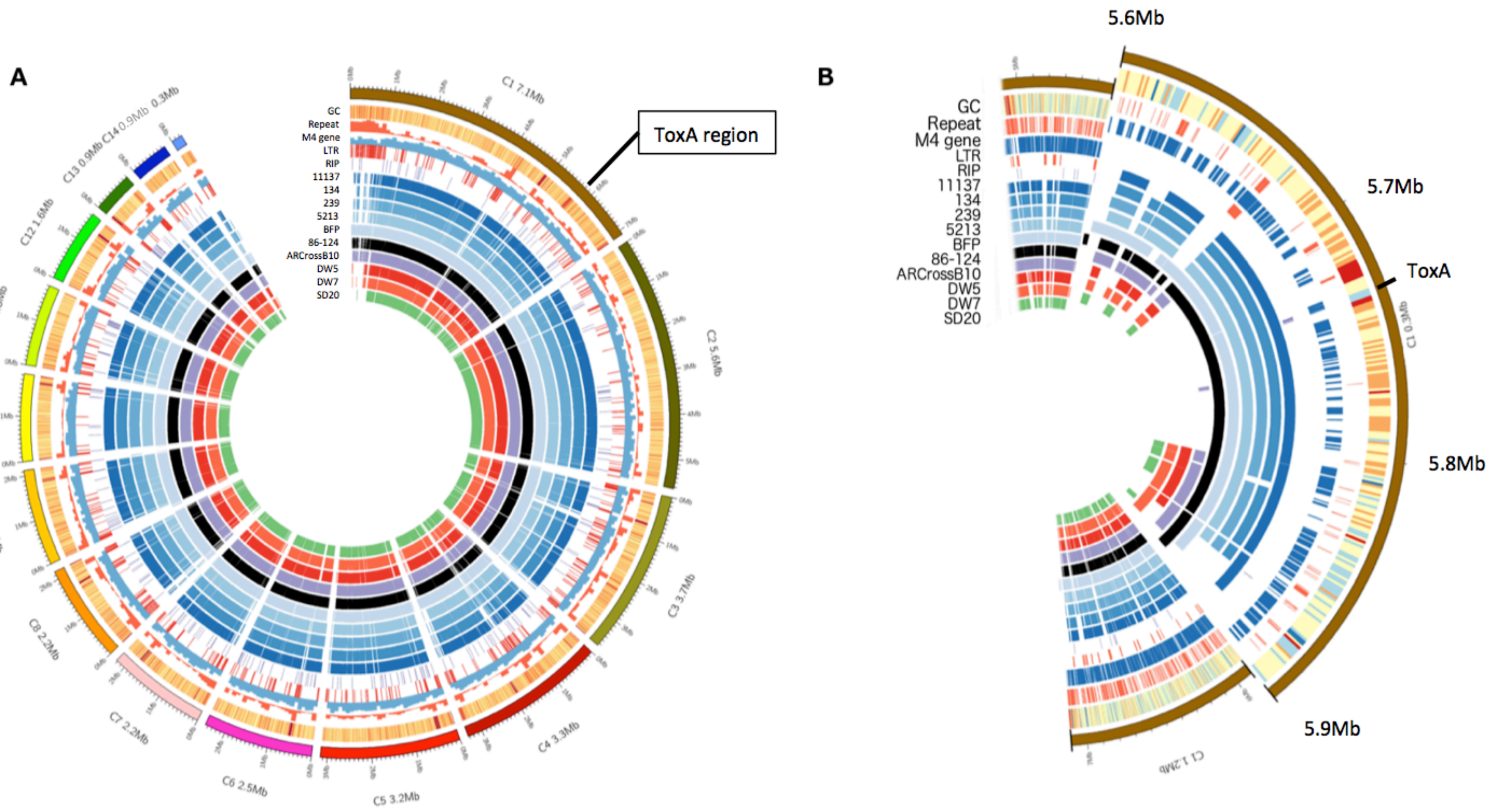

S20 Fig. Whole-genome overview of the gene and repeat features of the Australian M4 reference isolate, and comparisons to alternate Ptr isolate genomes.
