# Supplementary material for: Comparative genomics of the wheat fungal pathogen Pyrenophora tritici-repentis reveals chromosomal variations and genome plasticity
Source: BMC Genomics. 2018 Apr 23;19:279. doi: 10.1186/s12864-018-4680-3 (PMC5913888; doi:10.1186/s12864-018-4680-3)
Supplement: Supplementary file 22 — PacBio assembly methods and data. A) PacBio SMRT cell genome assembly flow chart overview showing input data (disc shapes) and tasks implemented (rectangles) from long read error correction and assembly through to final genome polishing (base error correction). B) PaBio SMRT cell M4 sample and sequencing statistics. (PDF 269 kb) [file 12864_2018_4680_MOESM22_ESM.pdf]

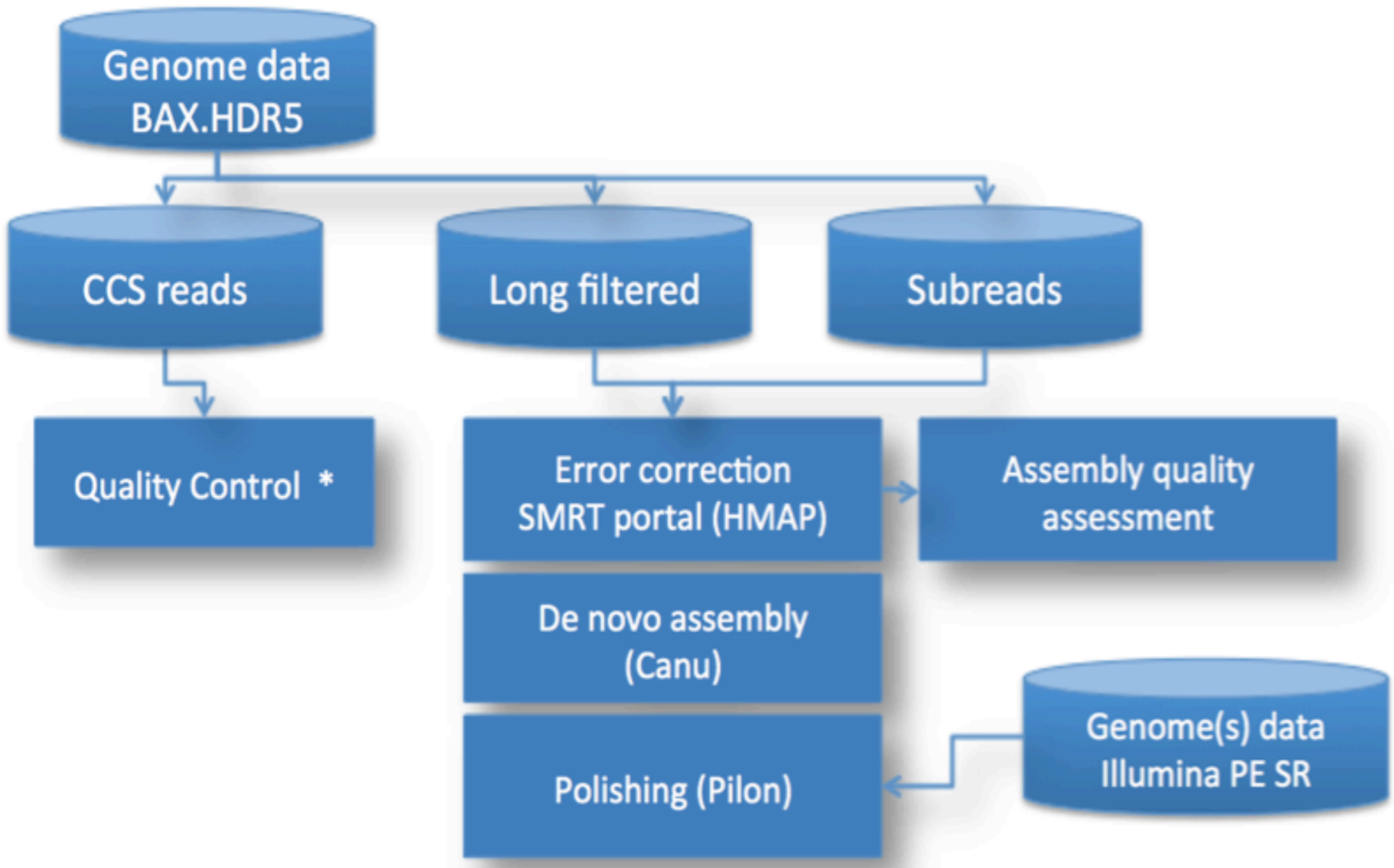

S22 Fig. PacBio assembly methods and data (1 of 2 slides)

Table. PaBio SMRT cell M4 sample and sequencing statistics

Run	Well	Cell Index	ZMW=0	ZMW=1	ZMW=2	Raw Avg Read length	Raw Avg Polymerase Speed	Raw Avg Read Quality
**Run226**	E01_1	4	42.20%	41.51%	16.29%	13,237	3.26	85
**Run226**	G01_1	6	35.05%	37.00%	27.95%	12,282	3.14	83
**Run226**	H01_1	7	46.14%	39.00%	14.86%	12,670	3.05	85
**Run226**	F01_1	5	42.24%	38.29%	19.47%	13,001	3.27	85
**Run228**	C01_1	2	34.84%	56.04%	9.12%	10,529	3.4	85
**Run228**	D01_1	3	46.82%	46.34%	6.84%	10,564	3.39	86

Sample Run	Subread Count	Subread Mean	Subread >3Kb Count	Subread >3Kb Mean	Subread >7Kb Count	Subread >7Kb Mean	Subread >12Kb Count	Subread >12Kb Mean
266	102,299	8,055	77,309	10,111	50,411	12,924	26,834	16,034
266	93,042	8,024	69,127	10,216	45,389	13,035	24,702	16,086
266	82,008	8,314	64,891	10,069	42,971	12,692	21,664	15,945
266	89,789	8,253	70,196	10,109	46,358	12,788	24,228	15,881
228	125,509	7,049	88,736	9,261	51,012	12,581	24,664	16,103
228	102,230	7,180	74,221	9,254	42,633	12,562	20,598	16,066

S14 Fig. PacBio assembly methods and data (2 of 2 slides)
