# Supplementary material for: Comparative genomics of the wheat fungal pathogen Pyrenophora tritici-repentis reveals chromosomal variations and genome plasticity
Source: BMC Genomics. 2018 Apr 23;19:279. doi: 10.1186/s12864-018-4680-3 (PMC5913888; doi:10.1186/s12864-018-4680-3)
Supplement: Supplementary file 23 — M4 Optical map compared to BFP in silico maps. (PDF 5715 kb) [file 12864_2018_4680_MOESM23_ESM.pdf]

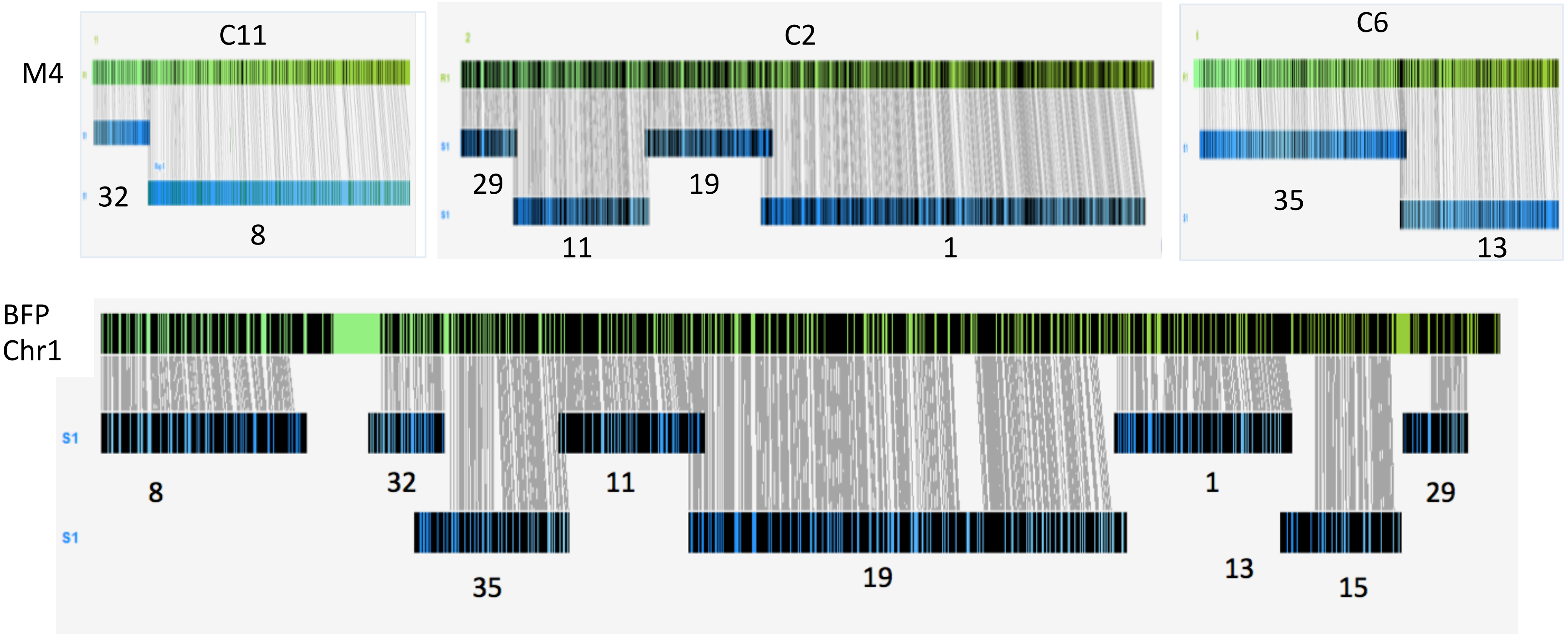

S Figure shows Nt.BbvC1 *in silico* maps (green) for M4 contigs 11, 2 & 6 (top) and BFP Chr1 (bottom) aligned to M4 Nt.BbvC1 restriction enzyme optical maps (blue)

S23 Fig. Optical mapping supporting data (page 1 of 10)

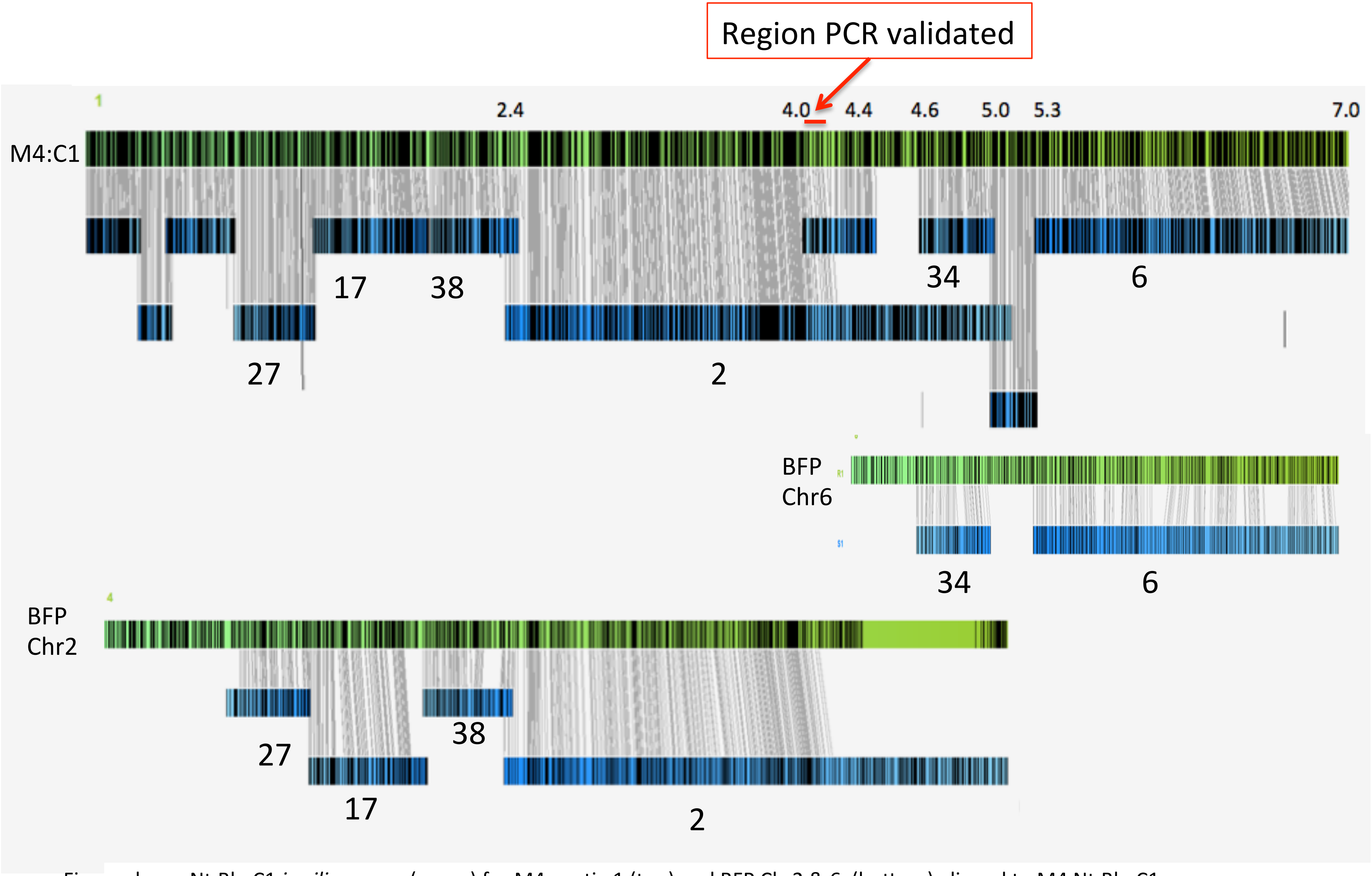

Figure shows Nt.BbvC1 *in silico* maps (green) for M4 contig 1 (top) and BFP Chr2 & 6 (bottom) aligned to M4 Nt.BbvC1 restriction enzyme optical maps (blue)

S23 Fig. Optical mapping supporting data (page 2 of 10)

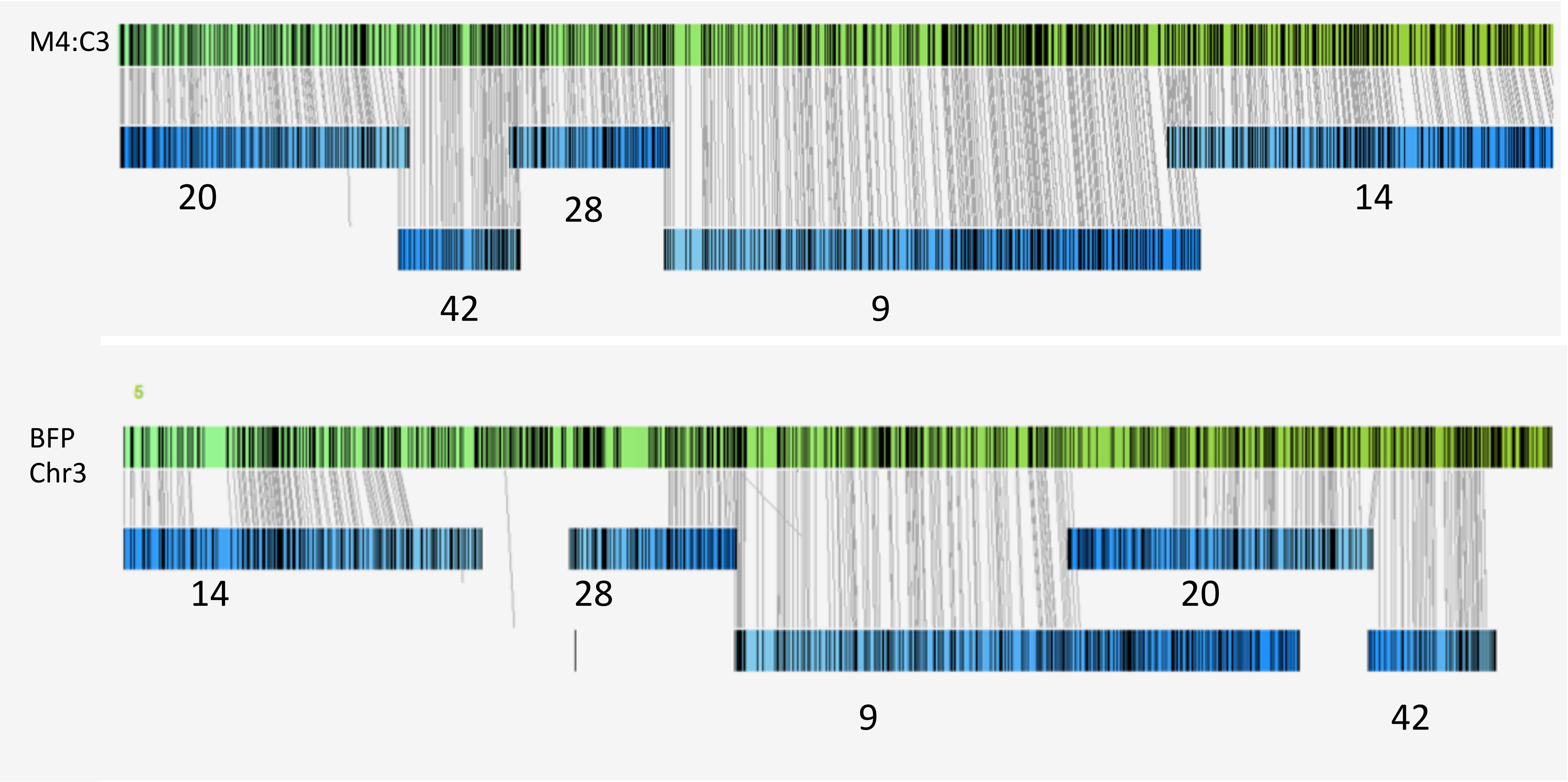

Figure shows Nt.BbvC1 *in silico* maps (green) for M4 contig3 (top) and BFP Chr3 (bottom) aligned to M4 Nt.BbvC1 restriction enzyme optical maps (blue)

S24 Fig. Optical mapping supporting data (page 3 of 10)

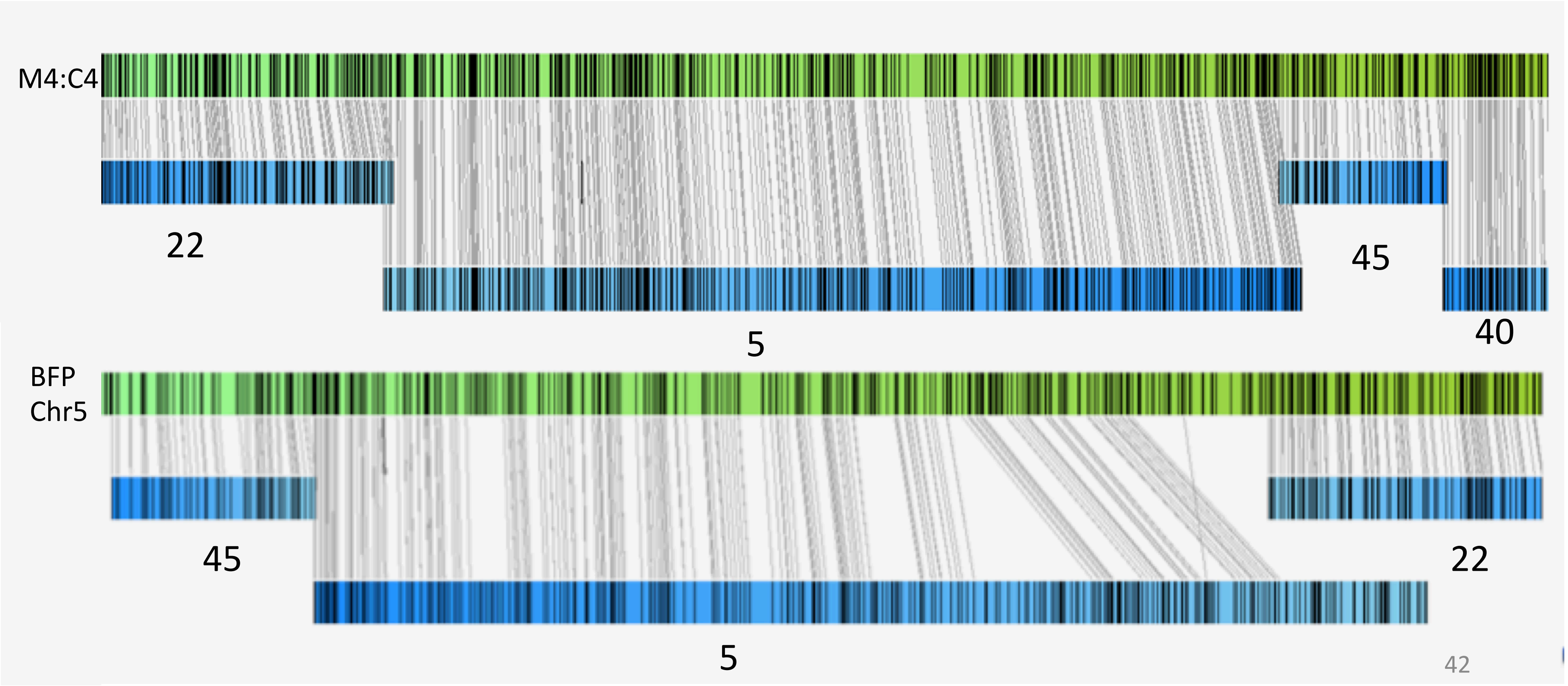

Figure shows Nt.BbvC1 *in silico* maps (green) for M4 contig4 (top) and BFP Chr5 (bottom) aligned to M4 Nt.BbvC1 restriction enzyme optical maps (blue)

S23 Fig. Optical mapping supporting data (page 4 of 10)

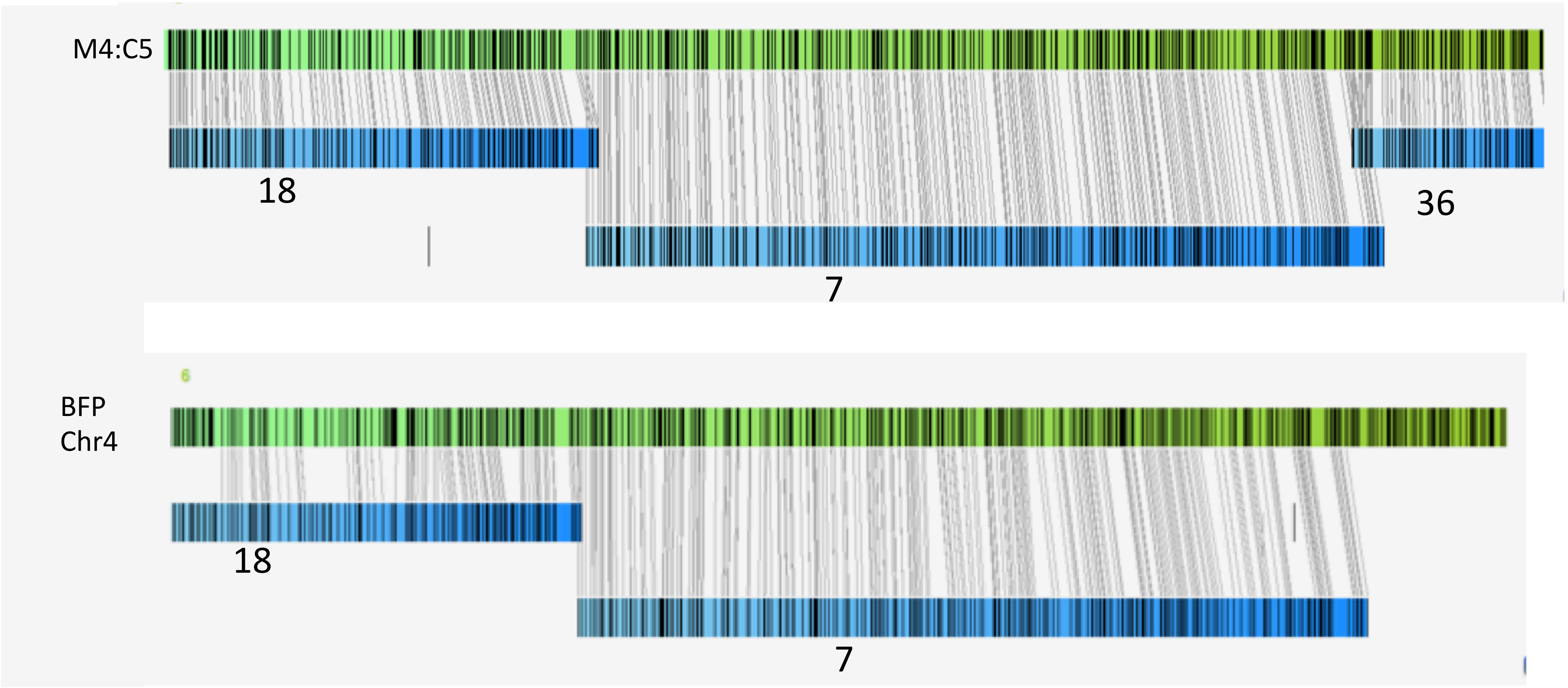

Figure shows Nt.BbvC1 *in silico* maps (green) for M4 contig5 (top) and BFP Chr4 (bottom) aligned to M4 Nt.BbvC1 restriction enzyme optical maps (blue)

S23 Fig. Optical mapping supporting data (page 5 of 10)

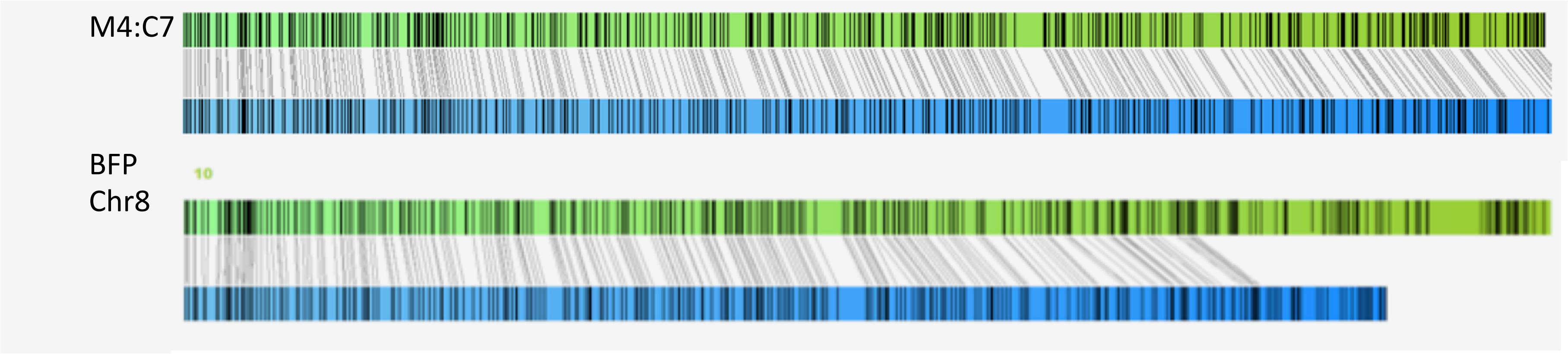

Figure shows Nt.BbvC1 *in silico* maps (green) for M4 contig7 (top) and BFP Chr8 (bottom) aligned to M4 Nt.BbvC1 restriction enzyme optical maps (blue)

S23 Fig. Optical mapping supporting data (page 6 of 10)

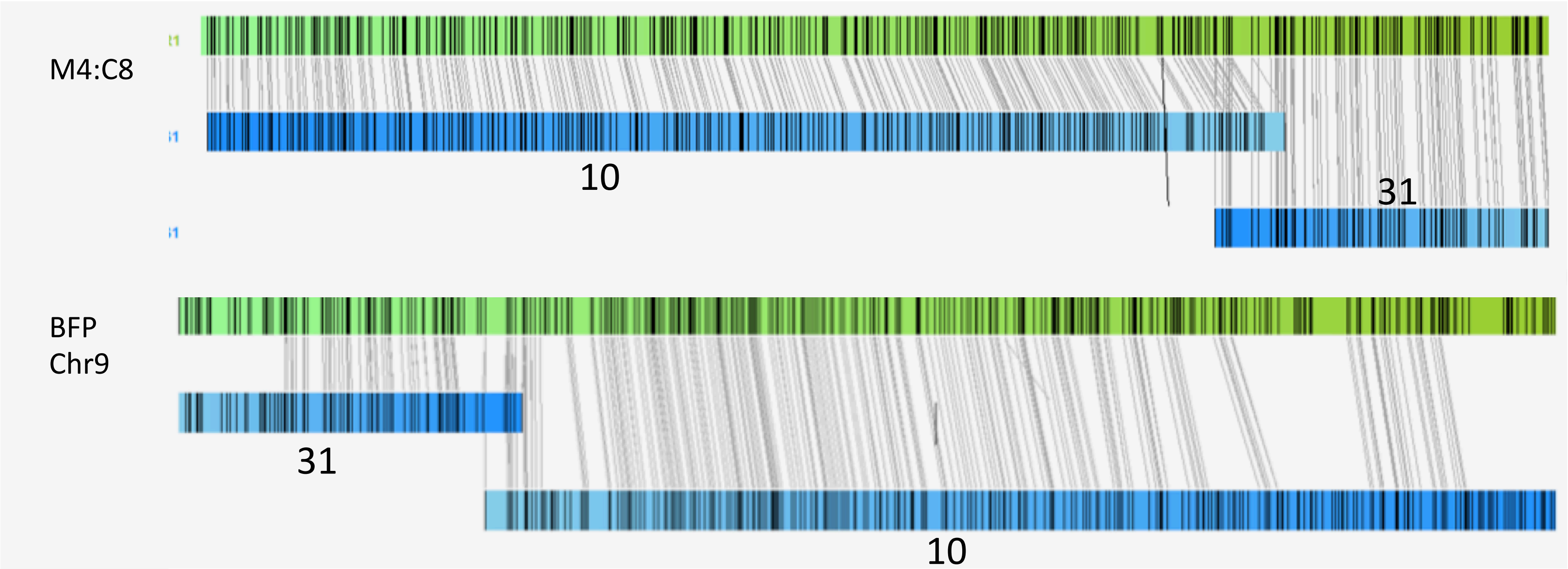

Figure shows Nt.BbvC1 *in silico* maps (green) for M4 contig8 (top) and BFP Chr9 (bottom) aligned to M4 Nt.BbvC1 restriction enzyme optical maps (blue)

S23 Fig. Optical mapping supporting data (page 7 of 10)

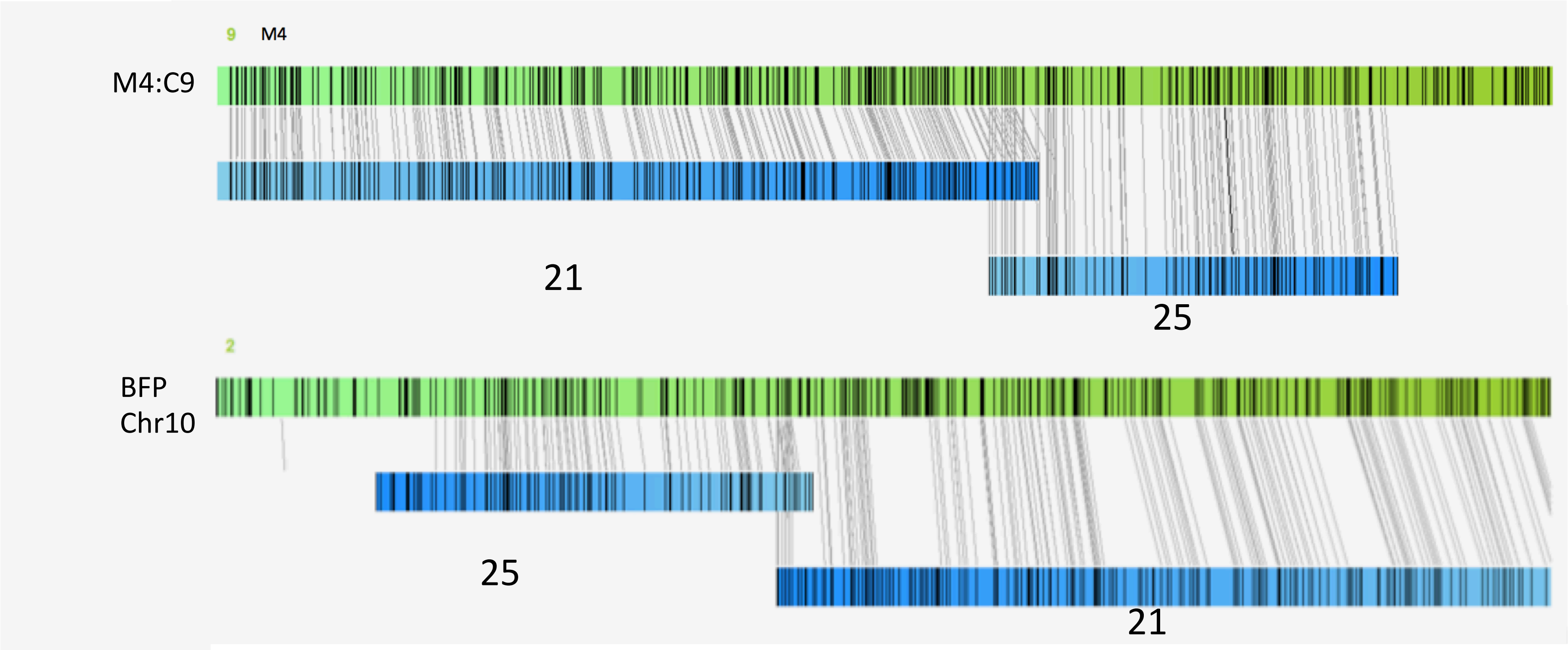

Figure shows Nt.BbvC1 *in silico* maps (green) for M4 contig9 (top) and BFP Chr10 (bottom) aligned to M4 Nt.BbvC1 restriction enzyme optical maps (blue)

S23 Fig. Optical mapping supporting data (page 8 of 10)

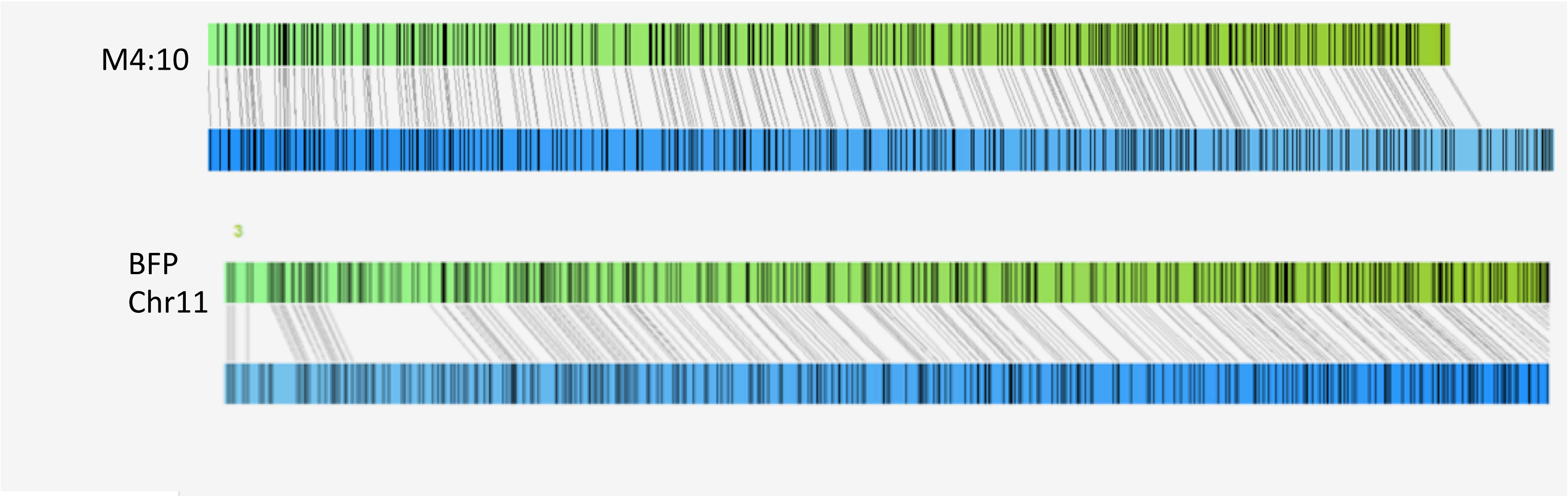

Figure shows Nt.BbvC1 *in silico* maps (green) for M4 contig10 (top) and BFP Chr11 (bottom) aligned to M4 Nt.BbvC1 restriction enzyme optical maps (blue)

S23 Fig. Optical mapping supporting data (page 9 of 10)

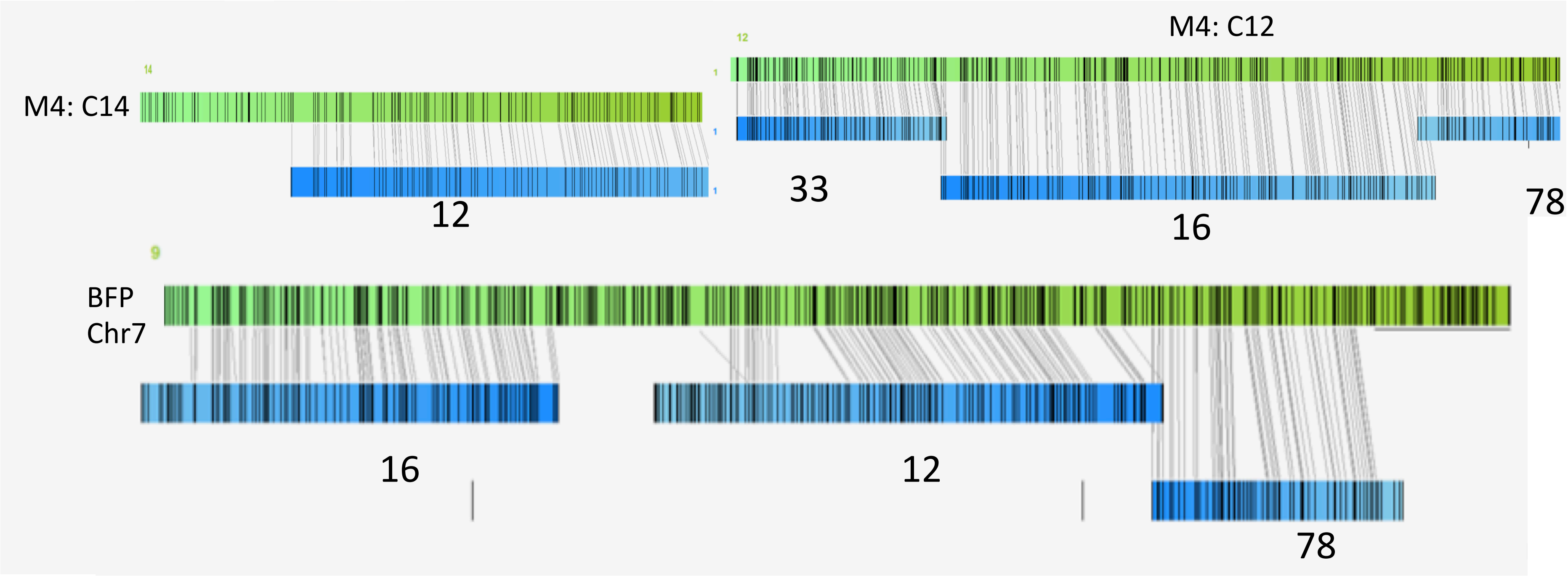

Figure shows Nt.BbvC1 *in silico* maps (green) for M4 contig 12 & 14 (top) and BFP Chr7 (bottom) aligned to M4 Nt.BbvC1 restriction enzyme optical maps (blue)

S23 Fig. Optical mapping supporting data (page 10 of 10)
